# A RUNX2 stabilization pathway mediates physiologic and pathologic bone formation

**DOI:** 10.1038/s41467-020-16038-6

**Published:** 2020-05-08

**Authors:** Jung-Min Kim, Yeon-Suk Yang, Kwang Hwan Park, Xianpeng Ge, Ren Xu, Na Li, Minkyung Song, Hyunho Chun, Seoyeon Bok, Julia F. Charles, Odile Filhol-Cochet, Brigitte Boldyreff, Teresa Dinter, Paul B. Yu, Ning Kon, Wei Gu, Takeshi Takarada, Matthew B. Greenblatt, Jae-Hyuck Shim

**Affiliations:** 1 Department of Medicine, University of Massachusetts Medical School, Worcester, MA USA; 2 Department of Orthopaedic Surgery, Yonsei University College of Medicine, Seoul, South Korea; 3 State Key Laboratory of Cellular Stress Biology, Xiamen University, Fujian, China; 4 Department of integrative biotechnology, Sungkyunkwan University, Suwon, South Korea; 5 Department of Mathematical Sciences, Korea Advanced Institute of Science and Technology, Daejeon, South Korea; 6 Department of Pathology and Laboratory Medicine, Weill Cornell Medical College, New York, NY USA; 7 Department of Orthopedics and Medicine, Brigham and Women’s Hospital, Harvard Medical School, Boston, MA USA; 8 INSERM U1036, pour le Vivant/Biologie du Cancer et de l’Infection, Commissariat à l’Énergie Atomique et aux Énerigies Alternatives Grenoble, Grenoble, France; 9 KinaseDetect ApS, 6340 Krusaa, Denmark; 10 Division of Cardiovascular Medicine, Department of Medicine, Brigham and Women’s Hospital, Harvard Medical School, Boston, MA USA; 11 Institute of Cancer Genetics, College of Physicians and Surgeons of Columbia University, New York, NY USA; 12 Department of Pathology and Cell Biology, College of Physicians and Surgeons of Columbia University, New York, NY USA; 13 Department of Regenerative Science, Okayama University Graduate School of Medicine, Okayama, Japan; 14 Li Weibo Institute for Rare Diseases Research, University of Massachusetts Medical School, Worcester, MA USA

**Keywords:** Bone development, Bone, Trauma

## Abstract

The osteoblast differentiation capacity of skeletal stem cells (SSCs) must be tightly regulated, as inadequate bone formation results in low bone mass and skeletal fragility, and over-exuberant osteogenesis results in heterotopic ossification (HO) of soft tissues. RUNX2 is essential for tuning this balance, but the mechanisms of posttranslational control of RUNX2 remain to be fully elucidated. Here, we identify that a CK2/HAUSP pathway is a key regulator of RUNX2 stability, as Casein kinase 2 (CK2) phosphorylates RUNX2, recruiting the deubiquitinase herpesvirus-associated ubiquitin-specific protease (HAUSP), which stabilizes RUNX2 by diverting it away from ubiquitin-dependent proteasomal degradation. This pathway is important for both the commitment of SSCs to osteoprogenitors and their subsequent maturation. This CK2/HAUSP/RUNX2 pathway is also necessary for HO, as its inhibition blocked HO in multiple models. Collectively, active deubiquitination of RUNX2 is required for bone formation and this CK2/HAUSP deubiquitination pathway offers therapeutic opportunities for disorders of inappropriate mineralization.

## Abstract (web summary)

Runx2 is essential for tuning the generation of bone from skeletal stem cells (SSCs). Here, the authors demonstrate that the CK2/HAUSP pathway stabilizes RUNX2 protein thereby regulating the commitment of SSCs to osteoprogenitors as well as their subsequent maturation, and that inhibition of this pathway can block heterotopic ossification.

## Introduction

As shown by both disorders of inappropriate bone formation, such as heterotopic ossification (HO) and disorders of deficient bone formation, such as osteoporosis, the capacity of skeletal stem cells (SSCs) to differentiate into bone-forming osteoblasts must be tuned in a context and tissue dependent manner. One of the best established molecular pathways regulating osteogenesis is the transcription factor RUNX2, which is required for both the commitment of skeletal progenitors to osteoprogenitors and the subsequent differentiation of osteoprogenitors^1,2^. As a master transcriptional regulator of skeletogenesis, RUNX2 is expressed in both mouse and human skeletal progenitors^3,4^. Mice with germline deletion of *Runx2* (*Runx2*^*−/−*^) show a complete absence of mineralized bone in both calvaria and long bones, suggesting that RUNX2 is required for both intramembranous and endochondral bone formation^5^. In addition, haploinsufficiency for *RUNX2* causes cleidocranial dysplasia (CCD), as characterized by open fontanels, hypoplastic clavicles, supernumerary teeth, and short stature, in both humans and mice^6,7^. On the other hand, excessive osteoblast differentiation can lead to disorders of ectopic mineralization, and RUNX2 is necessary for the pathogenesis of ectopic mineralization as shown in human HO patients and mouse HO models^8–11^. Thus, fine-tuning of RUNX2 expression and transcriptional activity is crucial for both physiologic and pathologic bone formation. While there are examples of regulating RUNX2 activity and stability via phosphorylation^12,13^, acetylation^14^, or ubiquitination^15–17^, how posttranslational mechanisms control RUNX2 in initial commitment to the osteoblast lineage and subsequently sustained to drive osteoblast differentiation remain to be fully elucidated.

Here we identify a key pathway stabilizing RUNX2 via Casein Kinase 2 (CK2 encoded by *Csnk2*)-mediated phosphorylation of RUNX2 leading to recruitment of the deubiquitinase herpesvirus-associated ubiquitin-specific protease (HAUSP, also known as USP7). HAUSP was identified as a regulator of the MDM2 and p53 pathway^18,19^. HAUSP also controls a wide array of substrates involved in immune responses, virus replication and infection, mitosis, DNA replication, and DNA damage repair^18–20^. CK2 is a constitutively active serine/threonine kinase that controls a multitude of signaling proteins linked to cell cycle progression, cell growth and differentiation^21,22^. CK2 is typically composed of a tetrameric complex including two catalytic α (*Csnk2a1*)- or α’ (*Csnk2a2*)-subunits and two regulatory β subunits (*Csnk2b*)^23–25^. The two catalytic subunits have high similarity in the catalytic domain, although there is tissue-specific functional specialization during embryonic development and as well as functional compensation dependent on their expression levels^26^. The C-terminal regions of the β-subunits bind the α-subunits and enhance the catalytic activity and stability of CK2 complex. Previous in vitro studies demonstrated a suppressive role of CK2 in osteoblast differentiation and bone morphogenetic protein (BMP) signaling^27,28^. However, its function in the skeleton has not been studied. Here, we demonstrate that CK2-induced phosphorylation of RUNX2 recruits the deubiquitinase HAUSP for the stabilization of RUNX2 and that this pathway is required for physiologic and pathologic bone formation.

## Results

### CK2 is required for osteoblast differentiation

**Fig. 1:**
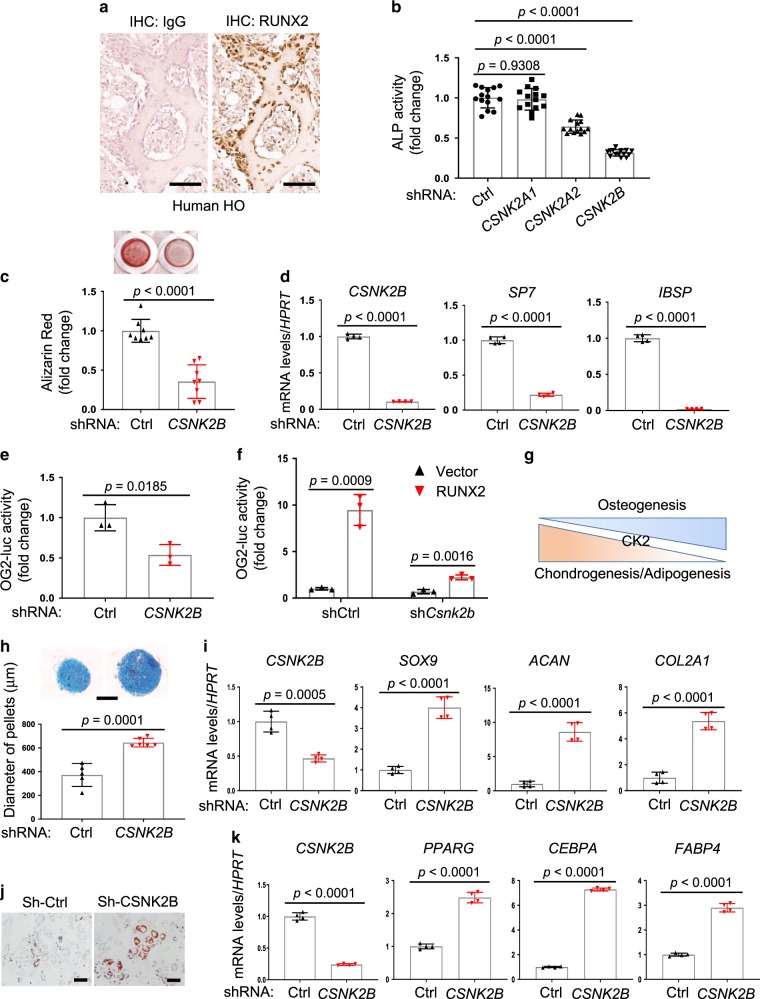
CK2 is required for BMSC osteoblast commitment. **a** Immunohistochemistry (IHC) for RUNX2 in human HO tissue. IgG was used for negative staining. Scale bar, 100 μm. **b**–**d** Human BMSCs expressing control shRNA (shCtrl) or shRNAs targeting *CSNK2A1*, -*2A2*, or -*2B* (sh*CSNK2A1*, sh*CSNK2A2*, sh*CSNK2B*) were cultured under osteogenic conditions. ALP activity was examined at day 7 (**b**), mineralization activity was assessed by alizarin red staining at day 14 (**c**), and expression of osteogenic genes was assessed by RT-PCR at day 12 (**d**) after osteogenic culture. **b** *n* = 14; **c**, *n* = 8; **d**, *n* = 4 biologically independent samples. shCtrl or sh*CSNK2B-*expressing human BMSCs (**e**) and shCtrl or sh*Csnk2b*-expressing C3H10T1/2 cells (**f**) were transfected with the RUNX2-responsive reporter gene (*OG2*-luc) and *Renilla* in the absence (**e**) or in the presence (**f**) of *RUNX2* overexpression. Three days after osteogenic culture (**e**) or 2 days after transfection (**f**), OG2-luc activity was measured and normalized to a *Renilla*. (*n* = 3 biologically independent samples). **g** Diagram depicting kinetics of CK2 expression over the differentiation of human BMSCs. **h**, **i** shCtrl or sh*CSNK2B*-epxressing human BMSCs were cultured under chondrogenic conditions for 21 days. After alcian blue staining, the size of chondrocyte pellets was measured (**h**). mRNA levels of chondrogenic genes were assessed by RT-PCR (**i**). Scale bar, 300 μm (**h**). **h** *n* = 5 (shCtrl) or 6 (sh*CSNK2B*); **i** *n* = 4 biologically independent samples. **j**, **k** shCtrl or sh*CSNK2B*-epxressing human BMSCs were cultured under adipogenic conditions for 12 days. Cells were stained with oil red O (**j**) and mRNA levels of adipogenic genes were assessed by RT-PCR (k). Scale bar, 100 μm (**j**). **k** *n* = 4 biologically independent samples. Data are representative of two (**a**) or three (**b**–**f**, **h**–**k**) independent experiments. Ordinary one-way ANOVA with Dunnett’s multiple comparisons test (**a**) and a two-tailed unpaired Student’s *t* test for comparing two groups (**c**–**f**, **h**, **i**, **k**; error bars, SD of biological replicates).

HO, abnormal bone formation in soft tissues, occurs sporadically after burns, traumatic brain injury, fractures and dislocations, and operative procedures, resulting in restricted joint mobility, severe pain, and nerve entrapment^29–32^. HO is postulated to reflect the aberrant differentiation of soft tissue-resident stem cells to osteoblasts^33^. Our data and others demonstrated elevated mRNA levels of *Runx2*, a key regulator of osteogenesis, in mouse HO tissues (Supplementary Fig. 14a), accompanied with a high expression of RUNX2 protein in human and mouse HO tissues (Fig. 1a and Supplementary Figs. 1b, 14c, f). Since RNAi-mediated knockdown of RUNX2 prevented HO in mice^8,9^, we aimed to identify the pathways controlling RUNX2. Previously, a high-throughput short hairpin RNA (shRNA) screen was performed in human bone marrow-derived mesenchymal stromal cells (BMSCs) using an alkaline phosphatase (ALP) assay, a marker of early osteoblast differentiation in order to identify kinases and/or phosphatases required for osteoblast differentiation^34,35^. In this study, shRNAs that suppress ALP activity in the primary screen were further examined in human BMSCs expressing a RUNX2-responsive luciferase reporter gene (OG2-luc) to identify kinases and/or phosphatases that specifically control RUNX2 activity/stability. This identified Casein Kinase 2 (CK2, CSNK2) as a putative regulator of BMSC differentiation into osteoblasts and RUNX2 activation. ALP activity was markedly reduced by knockdown of *CSNK2A2* or *CSNK2B*, but not *CSNK2A1* (Fig. 1b). In particular, *CSNK2B*-deficiency showed the strongest effect on ALP activity, extracellular matrix mineralization, and osteogenic gene expression (Fig. 1b–d and Supplementary Fig. 2a). Moreover, these BMSCs showed a significant reduction in transcriptional activity of RUNX2 (Fig. 1e, f). We next sought to identity the relevance of these findings in primary human osteogenic progenitors. While the human SSCs serving as a source of osteoblasts after growth plate closure are currently unclear, we noted the presence of cells bearing the same surface immunophenotype as previously described murine SSCs (Lin-Thy-CD200 + CD105- cells)^3,36^ present in bone marrow aspirates (BMA). The osteogenic potential of this population was substantially decreased when treated with the CK2 inhibitor (i-CK2, Casein Kinase II inhibitor IV) (Supplementary Fig. 3). Thus, CK2 is important for differentiation of SSCs into osteoblasts and RUNX2 activation.

As CK2 is a constitutively active kinase, it is primarily transcriptionally regulated^37,38^. Accordingly, both mRNA and protein levels of CK2 subunits increased during osteoblast differentiation, whereas their expression was downregulated in mature chondrocytes and adipocytes (Fig. 1g and Supplementary Fig. 2b–e). When cultured under chondrogenic conditions, *CSNK2B*-deficient BMSCs showed an increase in chondrogenic potential evidenced by larger chondrocyte pellet size and upregulated expression of chondrogenic genes (Fig. 1h, i). Likewise, under adipogenic conditions, formation of lipid droplets and expression of adipogenic genes were markedly upregulated by *CSNK2B*-deficiency (Fig. 1j, k). These results suggest that CK2 promotes osteoblast differentiation while it suppresses differentiation into chondrocytes or adipocytes.

### CK2 is essential for skeletal development

**Fig. 2:**
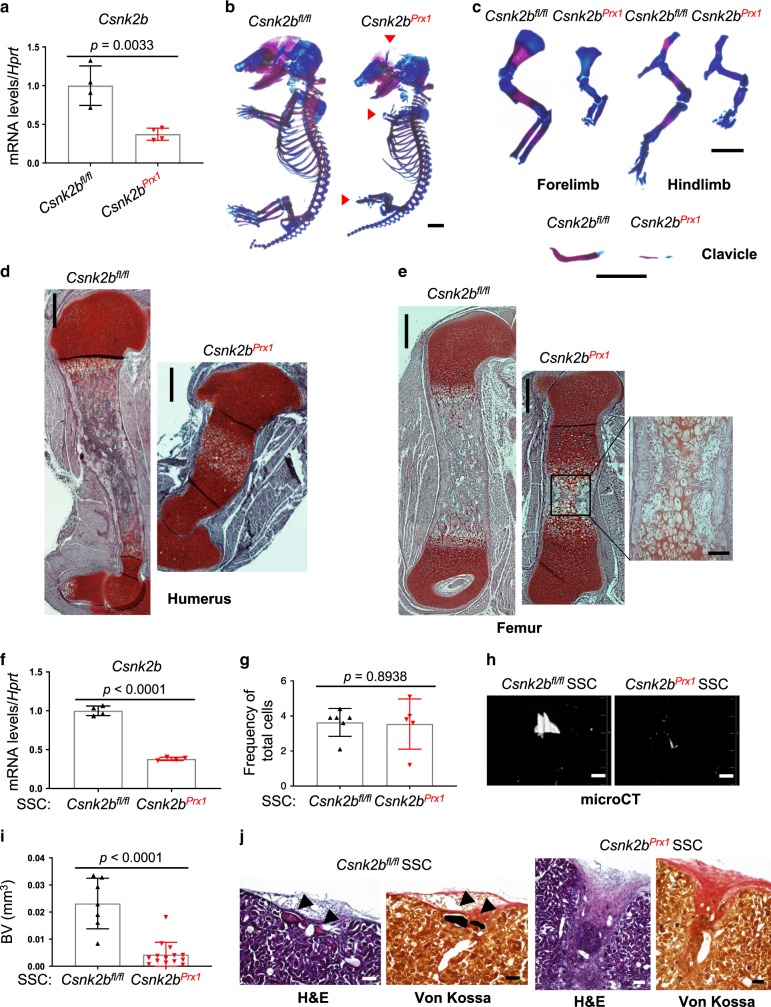
CK2 is required for bone formation during skeletal development. **a** *Csnk2b* mRNA levels in the hindlimbs (femur and tibia) of E17.5 *Csnk2b*^*fl/fl*^ and *Csnk2b*^*Prx1*^ embryos. (*n* = 4). **b**, **c** Alizarin red/alcian blue staining of skeletal preparations of E17.5 *Csnk2b*^*fl/fl*^ and *Csnk2b*^*Prx1*^ embryos. Scale bar, 1 mm. Safranin O staining of humeri (**d**) and femurs (**e**) of P0 *Csnk2b*^*fl/fl*^ and *Csnk2b*^*Prx1*^ pups. Scale bars, 250 μm (left) and 50 μm (right, enlarged one). **f**, **g** *Csnk2b* mRNA levels in SSCs (CD45^−^Ter119^−^Tie2^−^αV-Int^+^Thy1^−^6C3^−^CD105^−^CD200^+^) isolated from E17.5 *Csnk2b*^*fl/fl*^ and *Csnk2b*^*Prx1*^ embryos (**f**). Frequency of SSCs within the population of total skeletal cells (CD45^−^Ter119^−^Tie2^−^αV-Int^+^) (**g**). **f** *n* = 4; g, *n* = 6 (*Csnk2b*^*fl/fl*^) or 5 (*Csnk2b*^*Prx1*^)). **h**–**j** SSCs (CD45^−^Ter119^−^Tie2^−^αV-Int^+^Thy1^−^6C3^−^CD105^−^CD200^+^) isolated from E17.5 *Csnk2b*^*fl/fl*^ and *Csnk2b*^*Prx1*^ embryonic limbs were transplanted beneath the kidney capsule. MicroCT analysis shows 3D-reconstruction (**h**) and quantification (**i**) of bone mass in the kidney capsule. BV bone volume. Histologic sections of kidney capsule were stained with H&E (**j**, left) or Von Kossa (**j**, right). The arrow highlights the ectopic bone. Scale bars, 200 μm (**h**); 100 μm (**j**). **i** *n* = 7 or 14. Data are representative of three (**a**–**e**, **h**, **j**) independent experiments or are pooled from two experiments (**f**, **g**, **i**). A two-tailed unpaired Student’s *t* test for comparing two groups (**a**, **f**, **g**, **i**; error bars, SD of biological replicates).

To explore a role CK2 in skeletal development, expression of CSNK2B was examined at the stages of early limb and postnatal skeletal development (Supplementary Fig. 4). Immunohistochemistry (IHC) demonstrates the expression of CSNK2B in precursor cells residing in the perichondrium, osteoblasts in the trabecular bone, and chondrocytes in the growth plate at postnatal day 10 (P10). Its expression was also detected at the tip of the limb bud of E11.5 mouse embryos. Thus, CSNK2B is expressed throughout skeletal development from early embryonic stage to postnatal stage. Next, we conditionally deleted *Csnk2b* in the mesenchyme by crossing *Csnk2b* (*Csnk2b*^*fl/fl*^)^39,40^ floxed allele with a Prx1-Cre^41^ (*Csnk2b*^*Prx1*^). *Csnk2b* deletion was validated in the limbs dissected from P0 *Csnk2b*^*Prx1*^ neonates (Fig. 2a). Severe limb shortening was observed in E16.5 and P0 *Csnk2b*^*Prx1*^ pups and P0 *Csnk2b*^*Prx1*^ pups died after the birth due to respiratory distress (Supplementary Fig. 5a). Alizarin red and alcian blue staining of skeletal preparations revealed that ossification (red) was markedly reduced in the calvaria, scapula, humerus, radius, ulna, femur, tibia, fibula, digit, and sternum of *Csnk2b*^*Prxl*^ pups while cartilage (blue) is normally formed in skeleton (Fig. 2b, c and Supplementary Fig. 5b–f). Moreover, the clavicles of *Csnk2b*^*Prxl*^ pups were hypoplastic (Fig. 2c, bottom, and Supplementary Fig. 5c, top). Likewise, endochondral ossification of long bones was arrested at the earliest stages of primary ossification center formation (Fig. 2d, e and Supplementary Fig. 5g, h). These skeletal phenotypes are similar to those seen in *Runx2*^*Prx1*^ pups^5^, suggesting that CK2 is required for RUNX2 regulation.

Skeletal development occurs through a hierarchy of bone lineage-specific progenitors, and these progenitors can be isolated from mouse limbs based on the expression of cell surface markers^3^. Among these progenitors, we isolated homogenous populations of SSCs using recently described FACS strategies^3^ from the limbs of E17.5 *Csnk2b*^*fl/fl*^ and *Csnk2b*^*Prx1*^ embryos (Supplementary Fig. 6). *Csnk2b* was efficiently deleted in *Csnk2b*^*Prx1*^ SSCs (Fig. 2f). Absolute numbers of SSCs were comparable between *Csnk2b*^*fl/fl*^ and *Csnk2b*^*Prx1*^ embryonic limbs (Fig. 2g). However, while little cell death of these cells was observed, cell proliferation rate in *Csnk2b*^*Prx1*^ SSCs was slightly reduced in the culture (Supplementary Fig. 7a, b), implicating a potential role of CK2 in SSCs during embryonic development. To determine their osteogenic potential in vivo*, Csnk2b*^*fl/fl*^ and *Csnk2b*^*Prx1*^ SSCs were transplanted into the kidney capsule of secondary hosts and bone formation was assessed by microCT and histology. The transplanted *Csnk2b*^*Prx1*^ SSCs displayed a significant reduction in osteogenic potential despite engrafting and expanding within the kidney capsule (Fig. 2h–j). These findings suggest that CK2 is critical for SSC differentiation into osteoblasts but is dispensable for SSC survival.

### CK2 deletion causes CCD

**Fig. 3:**
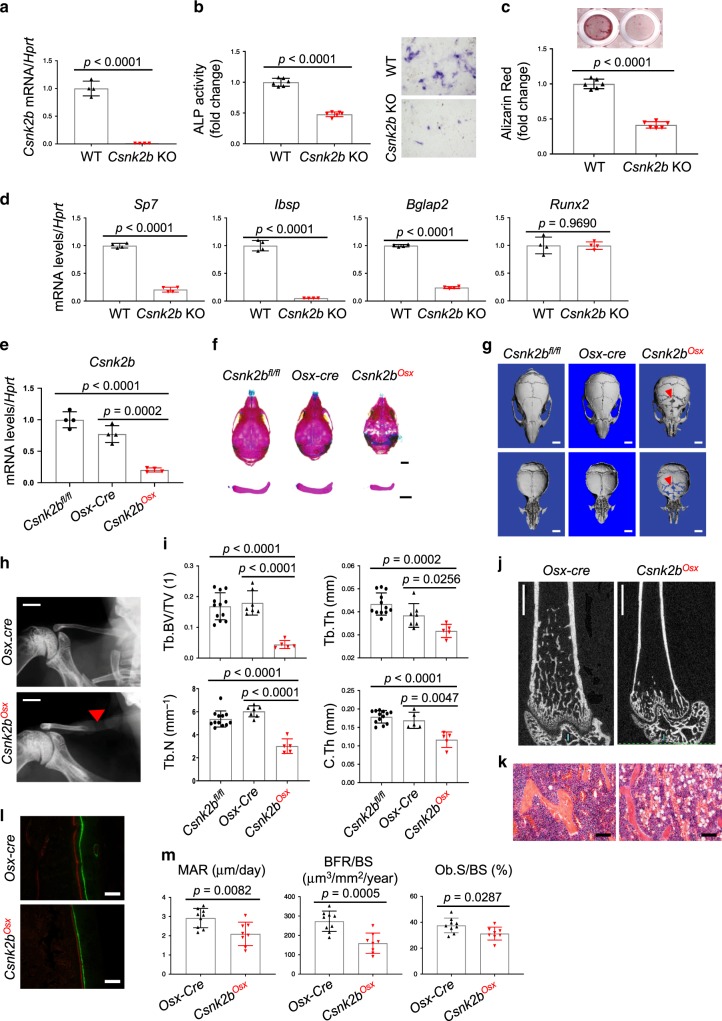
CK2 is required for maturation of osteoprogenitors in vitro and in vivo. **a**–**d** COBs isolated from P5 *Csnk2b*^*fl/fl*^ pups were infected with lentiviruses expressing vector (WT) or Cre recombinase (*Csnk2b* KO) and cultured under osteogenic conditions. mRNA levels of *Csnk2b* (**a**) and osteogenic genes (**d**) were measured by RT-PCR at day 12. ALP activity (**b**, left) and staining (**b**, right) or alizarin red staining (**c**) were performed at day 7 or 18, respectively. Scale bar, 250 μm (**b**). **a**, **d**, *n* = 4; **b**, **c**, *n* = 6. **e** *Csnk2b* mRNA levels in P0 calvaria of *Csnk2b*^*fl/fl*^, *Osx-Cre*, and *Csnk2b*^*Osx*^ pups. (*n* = 4). **f** Alizarin red/alcian blue staining of skeletal preparations of calvaria (top) and clavicles (bottom) obtained from P10 *Csnk2b*^*fl/fl*^, *Osx-Cre*, and *Csnk2b*^*Osx*^ pups. Scale bar, 2 mm. **g** MicroCT analysis shows 3D-reconstruction of calvaria from 2-month-old *Csnk2b*^*fl/fl*^, *Osx-Cre*, and *Csnk2b*^*Osx*^ male mice. The arrows indicate hypomineralization areas. Scale bar, 2 mm. **h** Radiographic images of clavicles from 2-month-old *Osx-Cre* and *Csnk2b*^*Osx*^ male mice. The arrow indicates defective sternoclavicular ossification. Scale bar, 2 mm. **i**, **j** MicroCT analysis of femurs from 2-month-old *Csnk2b*^*fl/fl*^, *Osx-Cre*, and *Csnk2b*^*Osx*^ male mice. Quantification (**i**) and 3D-reconstruction (**j**) are displayed. Trabecular bone volume/total volume Tb. BV/TV trabecular thickness, Tb.Th trabecular number per cubic millimeter, Tb.N, and C.Th cortical thickness. Scale bar, 1 mm (**j**). **i** Tb. BV/TV, Tb.Th, Tb.N; *n* = 12 (*Csnk2b*^*fl/fl*^), 7 (*Osx-Cre*) or 5 (*Csnk2b*^*Osx*^), C.Th; *n* = 13 (*Csnk2b*^*fl/fl*^), 5 (*Osx-Cre*) or 5 (*Csnk2b*^*Osx*^)). **k** H&E-stained longitudinal sections of femurs from 2-month-old *Osx-Cre* and *Csnk2b*^*Osx*^ male mice. Scale bar, 50 μm. **l**, **m** Histomorphometric analysis of femurs from 2-month-old *Osx-Cre* and *Csnk2b*^*Osx*^ female mice. Images of calcein/alizarin red-labeled sections of the femur (**l**) and quantification (**m**) are displayed. Scale bar, 50 μm (**l**). BFR/BS bone formation rate per bone surface, MAR mineral reposition rate, Ob.S/BS osteoblast surface per bone surface. (*n* = 9 (*Osx-Cre*) or 8 (*Csnk2b*^*Osx*^)). Similar skeletal phenotypes were observed in both male and female mice (**f**–**m**). Data are representative of three (**a**–**h**, **j**–**l**) independent experiments or are pooled from two experiments (**i**, **m**). A two-tailed unpaired Student’s *t* test for comparing two groups (**a**–**e**, **i**, **m**; error bars, SD of biological replicates).

To investigate the role of CK2 in osteoblast differentiation in vitro, primary calvarial osteoblasts (COBs) were isolated from P5 *Csnk2b*^*fl/fl*^ pups and infected with lentiviruses expressing vector or cre recombinase. This inducible deletion by cre induction can offer better control of the timing of induction, and more uniformly synchronous induction of cre activity than relying on the endogenous cre lines^13,42^. As expected, Cre expression mediated efficient *Csnk2b* deletion in *Csnk2b*^*fl/fl*^ COBs (Fig. 3a). In these COBs, osteoblast differentiation was substantially decreased as determined by ALP activity, mineralization, and expression of osteogenic genes, while levels of *Runx2* mRNA were not altered in the absence of *Csnk2b* (Fig. 3b–d). These phenotypes were also seen in COBs isolated from mice with deletion of *Csnk2b* in osteoprogenitors (*Csnk2b*^*Osx*^)^43,44^ (Supplementary Fig. 8). Likewise, *Csnk2b*^*Osx*^ mice displayed osteopenia and specific phenotypes characteristic of RUNX2 partial loss-of-function. P10 and 2-month-old *Csnk2b*^*Osx*^ mice displayed open calvarial fontanels, hypoplastic clavicles, and reduced body length (Fig. 3e–h and Supplementary Fig. 9a, b), similar to CCD seen in human patients with loss-of-function mutations in *RUNX2* or mice with *Runx2* haploinsufficiency^1,6,7^. In addition, *Csnk2b*^*Osx*^ mice displayed severe osteopenia in long bones as both the relative amount of trabecular bone and midshaft cortical bone thickness were both substantially reduced along with an increase in bone marrow adipocytes (Fig. 3i–k). Consistent with this, bone formation rate (BFR), mineralization apposition rate (MAR), and osteoblast surface per bone surface (Ob.S/BS) were decreased in *Csnk2b*^*Osx*^ femurs (Fig. 3l, m), demonstrating reduced differentiation of *Csnk2b*-deficient osteoblasts. Numbers of tartrate-resistant acid phosphatase (TRAP)-positive osteoclasts and serum levels of the bone resorption marker C-terminal telopeptide type I collagen (CTX) were unchanged in 2-month-old *Csnk2b*^*Osx*^ mice (Supplementary Fig. 9c–e). Taken together, CK2 is critical for osteoblast differentiation in vitro and in vivo, and loss of CK2 produces skeletal phenotypes similar to those seen with loss of RUNX2 activity.

### CK2 stabilizes RUNX2 in osteoblasts

**Fig. 4:**
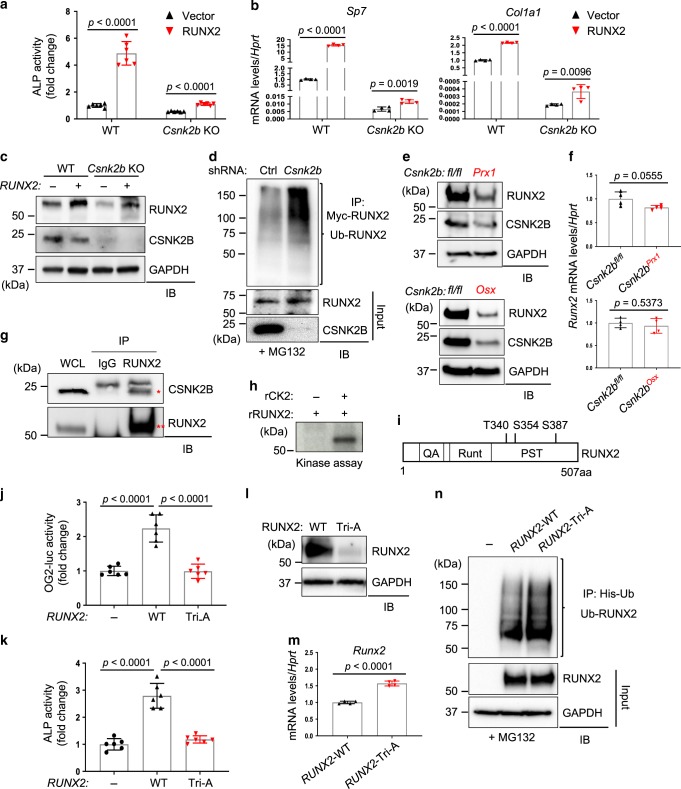
CK2-induced phosphorylation is required for RUNX2 stabilization in osteoblasts. **a**–**c** *Csnk2b*-sufficient or -deficient COBs were infected with lentiviruses expressing vector- or RUNX2 and cultured under osteogenic conditions for 6 days. ALP activity (**a**), mRNA levels of osteogenic gene (**b**), and protein levels of RUNX2 (**c**) were assessed. **a** *n* = 6; **b**, *n* = 4 biologically independent samples. **d** shCtrl or sh*Csnk2b*-expressing C3H10T1/2 cells were transfected with Myc-*RUNX2*. 2 days after transfection, cells were treated with 10 μM MG132 for 6 h, lysed, immunoprecipitated with anti-Myc conjugated agarose, and immunoblotted with anti-ubiquitin antibody. Protein (**e**) and mRNA (**f**) levels of Runx2 in P0 *Csnk2b*^*fl/fl*^ and *Csnk2b*^*Prx1*^ limbs (top) and P0 *Csnk2b*^*fl/fl*^ and *Csnk2b*^*Osx*^ calvaria (bottom). **f** *n* = 4 biologically independent samples). **g** Wild-type COBs were lysed, immunoprecipitated with anti-IgG control or anti-RUNX2 antibody and protein G-conjugated agarose, and immunoblotted with the indicated antibodies. Asterisk indicates CSNK2B; double asterisks indicate RUNX2; WCL whole cell lysate. **h** The kinase activity of CK2 was assessed by a cell-free kinase assay using recombinant CK2 (rCK2) and RUNX2 (rRUNX2). **i** A diagram depicting CK2-induced phosphorylation sites on RUNX2 as determined by phospho-mass spectrometry. **j** C3H10T1/2 cells were transfected with vector, *RUNX2*-WT or *RUNX2*-Tri-A mutant along with OG2-luc and *Renilla*. 2 days after transfection, OG2-luc activity was measured and normalized to *Renilla*. (*n* = 6). **k** Wild-type COBs were infected with lentiviruses expressing vector, *RUNX2*-WT or *RUNX2*-Tri-A mutant, cultured under osteogenic conditions for 6 days, and ALP activity was measured. (*n* = 6). *RUNX2*-WT or Tri-A mutant was transfected into C3H10T1/2 cells and 2 days later, protein (**l**) and mRNA (**m**) levels of RUNX2 were assessed. **m** *n* = 4. **n** HEK293T cells were transfected with vector, *RUNX2*-WT or *RUNX2*-Tri-A mutant along with His-ubiquitin. 2 days after transfection, cells were treated with 10 μM MG132 for 6 h, lysed, immunoprecipitated with Ni-NTA agarose, and immunoblotted with anti-RUNX2 antibody. Data are representative of three (**a**–**h**, **j**–**n**) independent experiments. A two-tailed unpaired Student’s *t* test for comparing two groups (**a**, **b**, **f**, **m**) and ordinary one-way ANOVA with Sidak’s multiple comparisons test (**j**, **k**; error bars, SD of biological replicates).

Next, the ability of RUNX2 to induce osteogenesis was assessed in the absence of *Csnk2b* following overexpression of RUNX2. Accompanied with its reduced transcriptional activity (Fig. 1f), RUNX2-induced ALP activity and expression of osteogenic genes were markedly decreased in *Csnk2b*-deficient COBs, demonstrating that CK2 is required for RUNX2-mediated osteoblast differentiation (Fig. 4a, b and Supplementary Fig. 10a). Intriguingly, *Csnk2b*-deficient COBs showed a significant reduction in protein levels of RUNX2 (Fig. 4c) while ubiquitination levels of RUNX2 were substantially increased in these cells after treatment with the proteasomal inhibitor MG132 (Fig. 4d). Similarly, a significant reduction in RUNX2 protein levels was seen in *Csnk2b*^*Prx1*^ SSCs (Supplementary Fig. 7d, e) and in P0 *Csnk2b*^*Prx1*^ limbs and P0 *Csnk2b*^*Osx*^ calvaria (Fig. 4e). However, *Runx2* mRNA levels were unaltered in these cells and tissues (Supplementary Fig. 7c and Fig. 4f), suggesting that CK2 controls RUNX2 expression at a posttranslational level.

To investigate CK2-mediated RUNX2 regulation, co-immunoprecipitation analysis was performed in wild-type COBs to test the physical interaction between CK2 and RUNX2 (Fig. 4g). In addition, an in vitro kinase assay using recombinant CK2 (rCK2) and RUNX2 (rRUNX2) was performed, demonstrating that CK2 directly phosphorylates RUNX2 (Fig. 4h). Mass spectrometry using recombinant RUNX2 (rRUNX2) and CK2 (rCK2) showed that rCK2 phosphorylated rRUNX2 at three sites clustered in the PST (proline/serine/threonine rich) domain at the C-terminal region of RUNX2, Thr340, Ser354, and Ser387 (human NM_004348 pThr340, pSer354, pSer387) (Fig. 4i). T340 was previously identified as a Protein Kinase A-mediated phosphorylation site^45^, whereas S354 and S387 have not been identified. To determine the functional contribution of these three phosphorylation sites to RUNX2 activity, these three sites were substituted to alanine in every combination of single, double, and triple mutants and their ability to induce RUNX2 activation was analyzed by a luciferase assay. Overall, the single or double mutants displayed only a modest effect on RUNX2 activity, while triple mutants (Tri-A) nearly ablated RUNX2 activity (Fig. 4j). Likewise, RUNX2-induced ALP activity and expression of osteogenic genes were substantially reduced in Tri-A-RUNX2 mutant-expressing COBs (Fig. 4k and Supplementary Fig. 10b), demonstrating that CK2-induced phosphorylation is crucial for RUNX2-mediated osteoblast differentiation. As observed in *Csnk2b*-deficient SSCs and osteoblasts, protein levels of Tri-A-RUNX2 were markedly reduced relative to WT-RUNX2 despite showing a slight elevation in the corresponding mRNA levels (Fig. 4l, m). This reduction in protein levels was accompanied by an increase in ubiquitination of the RUNX2-Tri-A mutant (Fig. 4n), suggesting that an increased susceptibility of the Tri-A-RUNX2 mutant to proteasomal degradation is responsible for its decreased function. Thus, CK2-induced phosphorylation mediates RUNX2 stabilization in osteoblasts by counteracting ubiquitin-dependent proteasomal degradation of RUNX2^15–17^.

### HAUSP controls osteogenesis via RUNX2 stabilization

**Fig. 5:**
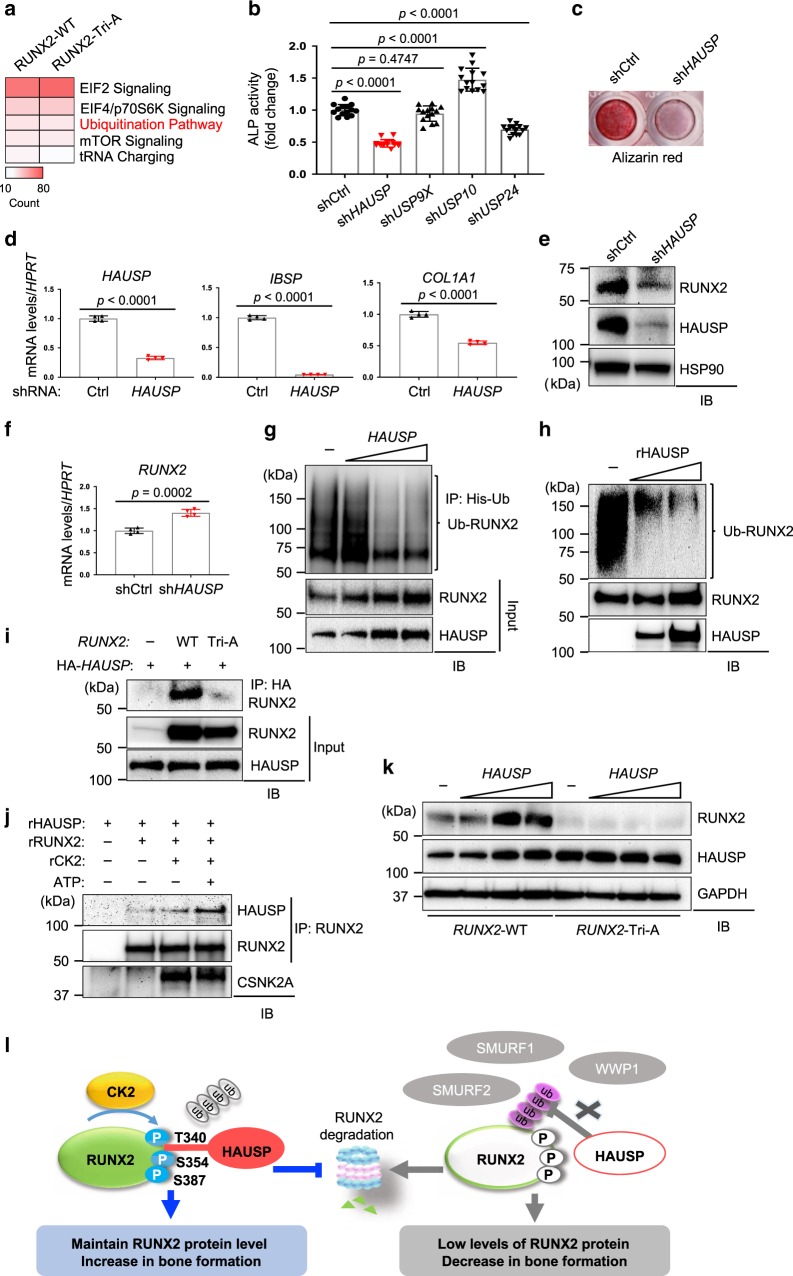
HAUSP regulates osteoblast differentiation by controlling RUNX2 stability. **a** Ingenuity pathway analysis of proteins that interact with *RUNX2*-WT and *RUNX2*-Tri-A mutant. **b**–**d** Human BMSCs expressing control shRNAs or shRNAs targeting the indicated DUBs were cultured under osteogenic conditions for 7 days and ALP activity was assessed (**b**). Mineralization (**c**) and mRNA levels of osteogenic genes (**d**) in human BMSCs expressing control (shCtrl) or HAUSP-targeting shRNAs (sh*HAUSP*) were assessed by alizarin red staining at day 18 and by RT-PCR at day 12, respectively. **b** *n* = 14; **d**, *n* = 4 biologically independent samples. Protein (**e**) and mRNA (**f**) levels of RUNX2 in shCtrl or sh*HAUSP*-expressing human BMSCs. **f** *n* = 4 biologically independent samples). **g** *RUNX2* and His-ubiquitin were transfected into HEK293T cells along with different concentrations of *HAUSP*. 2 days after transfection, cells were treated with 10 μM MG132 for 6 h, lysed, immunoprecipitated with Ni-NTA agarose, and immunoblotted with anti-RUNX2 antibody. **h** Cell-free deubiquitination analysis using recombinant HAUSP (rHAUSP) and ubiquitinated Flag-RUNX2 proteins. Flag-*RUNX2* and HA-ubiquitin were co-transfected into HEK293T cells and ubiquitinated Flag-RUNX2 proteins were obtained by immunoprecipitation with anti-Flag-conjugated agarose. **i** HEK293T cells were transfected with *RUNX2*-WT or *RUNX2*-Tri-A mutant along with HA-*HAUSP* and treated with 10 μM MG132 for 6 h prior to lysis. HA-HAUSP immunoprecipitates were immunoblotted with the indicated antibodies. **j** Cell-free interaction analysis using recombinant RUNX2 (rRUNX2), CK2 (rCK2), and HAUSP (rHAUSP). Recombinant proteins were incubated in the absence or presence of ATP for 30 min, immunoprecipitated with anti-RUNX2 antibody and protein G-conjugated agarose, and immunoblotted with the indicated antibodies. **k** *RUNX2*-WT or *RUNX2*-Tri-A mutant was transfected into HEK293T cells along with different concentrations of *HAUSP* and immunoblotted with the indicated antibodies. **l** Schematic diagram depicting a posttranslational regulation of RUNX2 by the CK2/HAUSP pathway in osteoblasts. Data are representative of three (**b**–**k**) independent experiments. Ordinary one-way ANOVA with Dunnett’s multiple comparisons test (**b**) and a two-tailed unpaired Student’s *t* test for comparing two groups (**d**, **f**; error bars, SD of biological replicates).

To identify potential regulators of RUNX2 ubiquitination in osteoblasts, affinity purification using Flag-RUNX2 (WT or Tri-A) followed by mass spectrometry was performed after balancing the expression of these two constructs (Supplementary Fig. 11a). Ingenuity pathway analysis (IPA) of RUNX2-binding proteins identified enrichment of several signaling pathways including the protein ubiquitination pathway (red, Fig. 5a). Specifically, this identified SMURF1, a known ubiquitin E3 ligase of RUNX2^15^, and the ubiquitin-specific protease (USP) family including HAUSP, USP9x, USP10, and USP47, as potential RUNX2-binding partners. Expression intensity of these USPs was markedly reduced in the eluate of Flag-RUNX2 (Tri-A) relative to Flag-RUNX2 (WT) (Supplementary Fig. 11b), suggesting that CK2-induced phosphorylation recruits these USPs to RUNX2. Knockdown studies in human BMSCs were used to determine which of these CK2-dependent RUNX2 interaction partners are important for osteoblast differentiation (Fig. 5b). Knockdown of HAUSP or USP24 but not USP9x or USP10 resulted in a significant reduction in ALP activity, mineralization, and expression of osteogenic genes (Fig. 5b–d and Supplementary Fig. 11c). Notably, similar to CK2, knockdown of HAUSP decreased protein but not mRNA levels of RUNX2 (Fig. 5e, f). HAUSP is an evolutionarily conserved deubiquitinating enzyme (DUB) and plays role in multiple cellular process^46^. However, the function of HAUSP in osteoblast biology remains largely unknown.

In vitro deubiquitination assays demonstrate that ubiquitination levels of RUNX2 were substantially reduced by either enforced expression of HAUSP or the addition of recombinant HAUSP (rHAUSP) (Fig. 5g, h). Furthermore, co-immunoprecipitation confirmed an interaction between HAUSP and RUNX2 (Fig. 5i). However, unlike HAUSP, USP24 was unable to interact with RUNX2 and reduce ubiquitination levels of RUNX2 (Supplementary Fig. 12), suggesting that HAUSP, not USP24, functions as a bona-fide RUNX2-DUB. This process requires CK2-induced phosphorylation, as the interaction between RUNX2 and HAUSP was markedly reduced in the presence of the RUNX2-Tri-A mutation (Fig. 5i). Likewise, the interaction between rRUNX2 and rHAUSP was enhanced in a cell-free system by the addition of rCK2 and ATP but not rCK2 alone (Fig. 5j). In addition, while enforced expression of HAUSP increased protein levels of RUNX2-WT, the RUNX2-Tri-A mutant was largely refractory to HAUSP-induced stabilization (Fig. 5k). Taken together, CK2-induced phosphorylation of RUNX2 recruits HAUSP, stabilizing RUNX2 through deubiquitination (Fig. 5l).

### *Hausp* haploinsufficiency causes CCD

**Fig. 6:**
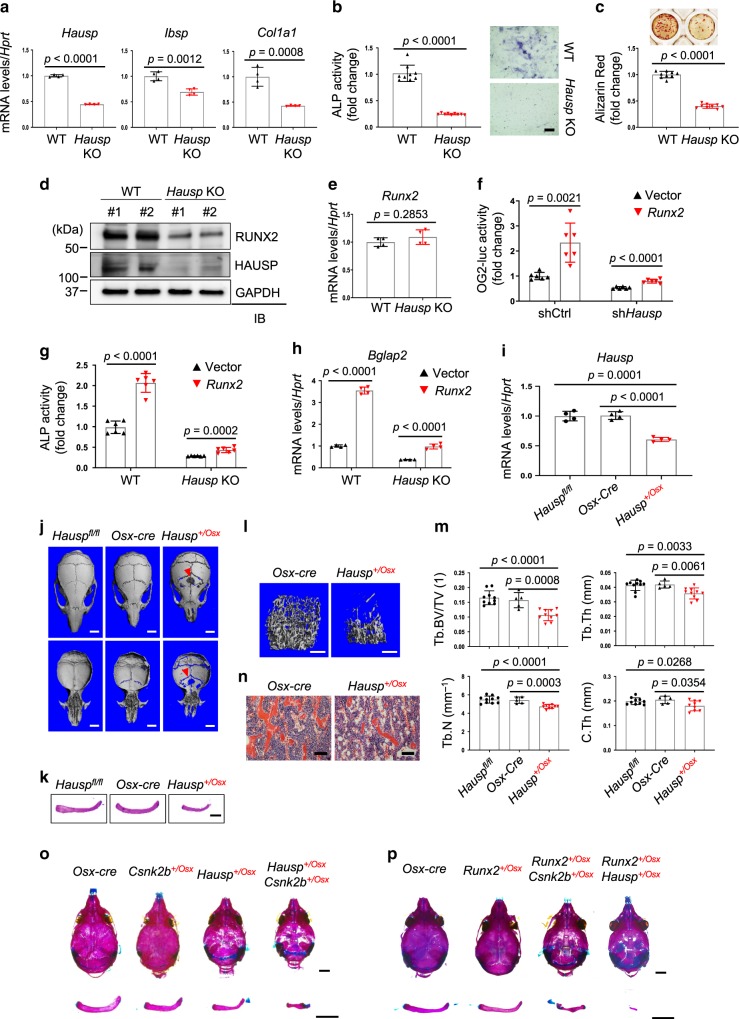
HAUSP is required for maturation of osteoprogenitors in vitro and in vivo. **a**–**e** COBs isolated from P5 *Hausp*^*fl/fl*^ pups were infected with lentiviruses expressing vector (WT) or Cre recombinase (*Hausp* KO) and cultured under osteogenic conditions. RT-PCR analysis to measure mRNA levels of *Hausp* and osteogenic genes (**a**) and ALP activity (**b**, left) and staining (b, right) were performed 7 days after the culture. Mineralization was assessed by alizarin red staining 16 days after the culture (**c**). Protein (**d**) and mRNA (**e**) levels of RUNX2 were assessed in these COBs. Scale bar, 100 μm (**b**). **a**, **e** *n* = 4; **b**, **c** *n* = 9. **f** shCtrl or sh*Hausp*-expressing C3H10T1/2 cells were transfected with OG2-luc and *Renilla* in the presence or absence of *RUNX2* overexpression. Two days after transfection, OG2-luc activity was measured and normalized to *Renilla*. (*n* = 6 biologically independent samples). *Hausp*-sufficient or -deficient COBs were infected with lentiviruses expressing vector or *RUNX2*, cultured under osteogenic conditions for 7 days, and ALP activity (**g**) and mRNA levels of *Bglap2* (**h**) were assessed. **g** *n* = 6; **h** *n* = 4 biologically independent samples). **i** *Hausp* mRNA levels in P0 *Hausp*^*fl/fl*^, *Osx-Cre*, and *Hausp*^*+/Osx*^ calvaria. (*n* = 4). **j**, **l**, **m** MicroCT analysis shows 3D-reconstruction of calvaria (**j**) and femurs (**l**) from 2-month-old *Hausp*^*fl/fl*^, *Osx-Cre* and *Hausp*^*+/Osx*^ male mice. Quantification of femoral bone mass is displayed (m). The arrows indicate hypomineralization areas. Scale bars, 2 mm (**j**); 500 μm (**l**). **m** *n* = 10 (*Hausp*^*fl/fl*^), 5 (*Osx-Cre*) or 10 (*Hausp*^*+/Osx*^). **k** Alizarin red/alcian blue staining of skeletal preparations of clavicles obtained from 2-month-old *Hausp*^*fl/fl*^, *Osx-Cre* and *Hausp*^*+/Osx*^ male mice. Scale bar, 2 mm. **n** H&E-stained longitudinal sections of femurs from 2-month-old *Osx-Cre* and *Hausp*^*+/Osx*^ male mice. Scale bar, 50 μm. Alizarin red/alcian blue staining of skeletal preparations of calvaria (top) and clavicles (bottom) from P10 *Osx-Cre*, *Csnk2b*^*+/Osx*^, *Hausp*^*+/Osx*^, and *Csnk2b*^*+/Osx*^;*Hausp*^*+/Osx*^ mice (**o**) and *Osx-Cre*, *Runx2*^*+/Osx*^, *Runx2*^*+/Osx*^;*Csnk2b*^*+/Osx*^, *and Runx2*^*+/Osx*^;*Hausp*^*+/Osx*^ mice (**p**). Scale bars, 2 mm (**o**, **p**). Data are representative of three (**a**–**l**, **n**–**p**) independent experiments or are pooled from two experiments (**m**). A two-tailed unpaired Student’s *t* test for comparing two groups (**a**–**c**, **e**–**i**, **m**; error bars, SD of biological replicates).

Similar to knockdown of *HAUSP* in human BMSCs, *Hausp*-deficient COBs show a markedly decreased ability to induce osteoblast differentiation as determined by a significant reduction in expression of osteogenic genes, ALP activity, and mineralization (Fig. 6a–c). In addition, RUNX2 protein was reduced in *Hausp*-deficient COBs without alteration of the corresponding mRNA levels (Fig. 6d, e), similar to the phenotypes shown in *Csnk2b*-deficient COBs. Accordingly, *Hausp*-deficient osteoblasts were refractory to RUNX2-induced transcriptional activation (Fig. 6f) and osteoblast differentiation (Fig. 6g, h and Supplementary Fig. 13a). Thus, HAUSP is permissive for osteoblast differentiation through stabilization of RUNX2.

Since the function of HAUSP in skeletal development was undefined, expression of HAUSP was examined in limb bud from E11.5 embryo and skeletal components (perichondrium cells, osteoblasts, and chondrocytes) from P10 mouse, and it showed similar expression aspects found in CSNK2B expression (Supplementary Fig. 4). To investigate a role of HAUSP in osteoblasts in vivo, *Hausp*^*fl/fl*^ mice were crossed with *Osx-Cre* mice. Due to the extremely low birth rate of pups with homozygous deletion of *Hausp* (*Hausp*^*Osx*^*)*, skeletal phenotypes were studied in mice with a heterozygous deletion of *Hausp* (*Hausp*^*+/Osx*^) (Fig. 6i). As observed in *Runx2*^*+/−*^ and *Csnk2b*^*osx*^ mice, *Hausp*^*+/Osx*^ mice developed CCD phenotypes, as characterized by open fontanels, hypoplastic clavicles, and short stature (Fig. 6j, k). In addition, substantial osteopenia was observed in both the trabecular and cortical compartments, which was accompanied by an increase in bone marrow adipocytes (Fig. 6l–n). However, numbers of TRAP-positive osteoclasts and resorption activity in the metaphysis of trabecular bone were not altered in *Hausp*^*+/Osx*^ mice relative to *Osx-Cre* or *Csnk2b*^*+/Osx*^ mice (Supplementary Fig. 13c, d), demonstrating that osteoclasts are normal in *Hausp*^*+/Osx*^ mice. Thus, similar to CK2, HAUSP controls osteoblast differentiation via RUNX2 stabilization.

Finally, we investigated whether CK2 and HAUSP cooperate to control the RUNX2 pathway in vivo. The genetic interaction between RUNX2, CK2, and HAUSP was examined by comparing skeletal phenotypes of *Osx-Cre*, *Csnk2b*^*+/Osx*^, *Hausp*^*+/Osx*^, *Csnk2b*^*+/Osx*^;*Hausp*^*+/Osx*^, *Runx2*^*+/Osx*^*, Runx2*^*+/Osx*^;*Csnk2b*^*+/Osx*^, and *Runx2*^*+/Osx*^; *Hausp*^*+/Osx*^ mice (Fig. 6o, p and Supplementary Fig. 13b). The CCD phenotypes seen in *Hausp*^*+/Osx*^ mice became more pronounced by additional heterozygous deletion of *Csnk2b* (*Csnk2b*^*+/Osx*^;*Hausp*^*+/Osx*^ mice) (Fig. 6o). Of note, chondrocyte development in the growth plate is relatively normal in these mice (Supplementary Fig. 13e). Likewise, addition of heterozygous deletion of *Csnk2b* (*Runx2*^*+/Osx*^;*Csnk2b*^*+/Osx*^ mice) or *Hausp* (*Runx2*^*+/Osx*^;*Hausp*^*+/Osx*^ mice) results in more severe CCD phenotypes in *Runx2*^*+/Osx*^ mice (Fig. 6p). Thus, these results suggest that CK2 and HAUSP operate in the same pathway to regulate RUNX2 in osteoprogenitors.

### Inhibition of the CK2-HAUSP pathway suppresses HO

**Fig. 7:**
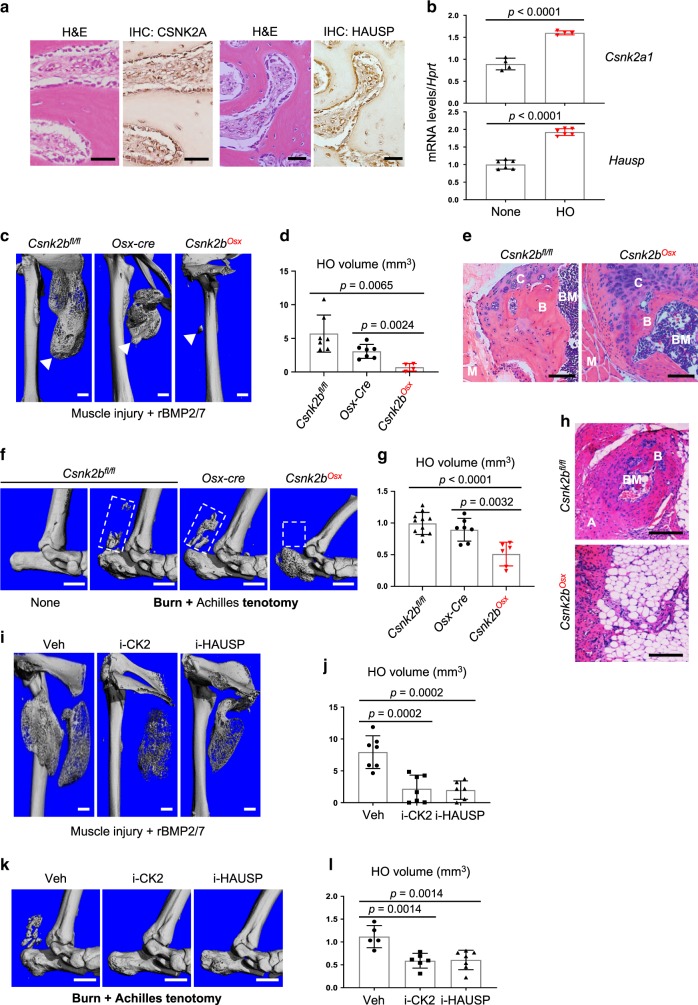
CK2/HAUSP pathway is required for acquired HO development. **a** H&E-stained sections (left) and immunohistochemistry (right) for CSNK2A and HAUSP in human HO tissue. Scale bar, 50 μm. **b** Achilles tenotomy was performed at 3-month-old male mice. Eight hours after the injury, Achilles tendon was dissected and mRNA levels of *Csnk2a1*, and *Hausp* were measured by RT-PCR. None, non-tenotomized Achilles tendon; HO, tenotomized Achilles tendon. (*n* = 4 (*Csnk2a1*) or 6 (*Hausp*)). **c**–**e** Quadriceps muscle injury by an aluminum ball drop was performed in 3-month-old *Csnk2b*^*fl/fl*^, *Osx-Cre*, and *Csnk2b*^*Osx*^ male mice, followed by injection of a mixture of rBMP2/7 and matrigel to the injured muscle. HO was assessed by microCT and histology 3 weeks post injury. 3D-reconstruction (**c**) and quantification (**d**) and H&E-stained sections of HO areas in the muscle (**e**) are displayed. The arrows indicate HO in muscle. B bone, M muscle, C cartilage, BM bone marrow. Scale bars, 1 mm (**c**); 100 μm (**e**). **d** *n* = 7 (*Csnk2b*^*fl/fl*^), 7 (*Osx-Cre*) or 4 (*Csnk2b*^*Osx*^). **f**–**h** Achilles tenotomy was performed in 3-month-old *Csnk2b*^*fl/fl*^, *Osx-Cre*, and *Csnk2b*^*Osx*^ male mice following with a remote burn injury on the back using a heated aluminum block. HO was assessed by microCT and histology 8 weeks post injury. 3D-reconstruction (**f**) and quantification (**g**) and H&E-stained sections of HO areas in the boxes of the microCT images (**h**) are displayed. None non-tenotomized leg, A Achilles tendon, BM bone marrow, B bone. Scale bars, 1 mm (**f**); 100 μm (**h**). **g** *n* = 11 (*Csnk2b*^*fl/fl*^), 7 (*Osx-Cre*) or 6 (*Csnk2b*^*Osx*^). **i**–**l** 3-month-old wild-type male mice were daily treated with DMSO (Veh) or an inhibitor of CK2 (i-CK2, 2.5 mg/kg) or HAUSP (i-HAUSP, 2 mg/kg) via intraperitoneal (i.p.) injection one day after muscle injury and BMP2/7-matrigel injection (**i**, **j**) or burn injury and Achilles tenotomy (**k**, **l**). HO was assessed by microCT analysis 3 weeks (**i**, **j**) or 8 weeks (**k, l**) post injury. 3D-reconstruction (**i**, **k**) and quantification (**j**, **l**) are displayed. Scale bars, 1 mm (**i**, **k**). **j**, *n* = 7 (DMSO), 7 (i-CK2), or 6 (i-HAUSP); **l** *n* = 5 (DMSO), 6 (i-CK2) or 7 (i-HAUSP). Data are representative of three (**a**–**c**, **e**, **f**, **h**, **i**, **k**) independent experiments or are pooled from two experiments (**d**, **g**, **j**, **l**). A two-tailed unpaired Student’s *t* test for comparing two groups (**b**, **d**, **g**) and ordinary one-way ANOVA with Dunnett’s multiple comparisons test (**j**, **l**; error bars, SD of biological replicates).

IHC on human HO tissue identified expression of CSNK2A and HAUSP in osteoblasts on the surface of heterotopic bone (Fig. 7a), which, when taken together with the expression of RUNX2 in the same tissue (Supplementary Fig. 1b), suggests that the CK2-HAUSP-RUNX2 pathway is intact at sites of HO. Expression levels of *Csnk2a1* and *Hausp* were all markedly upregulated at the site of injury in a mouse model of acquired HO (Fig. 7b). Thus, we hypothesized that RUNX2 stabilization by the CK2-HAUSP pathway contributes to the pathogenesis of HO and that inhibition of this pathway may prevent HO. To test this, mouse models representing the highest incidence forms of HO, muscle injury/BMP-induced HO^29,47^ and burn injury/Achilles tenotomy-induced HO^48,49^, were employed in *Csnk2b*^*fl/fl*^, *Osx-Cre*, and *Csnk2b*^*Osx*^ mice. In *Csnk2b*^*fl/fl*^ and *Osx-Cre* control mice, HO occurred 3 weeks after blunt muscle trauma and administration of recombinant BMP2/7 (BMP2/7) (muscle injury/BMP-induced HO; Fig. 7c). Likewise, Achilles tenotomy following a remote burn injury resulted in HO at the tenotomy site 8 weeks post injury (burn/Achilles tenotomy-induced HO; Fig. 7f). Heterotopic bone was formed via an endochondral pathway including formation of cartilage and adipose tissues and recruitment of hematopoietic elements (Fig. 7e, left and h, top). By contrast, *Csnk2b*^*Osx*^ mice displayed a significant reduction in HO while cartilage or adipose tissue was still formed at the injury site (Fig. 7c–h), demonstrating that CK2 is required for heterotopic bone formation in mouse models of acquired HO. Notably, protein levels of RUNX2 were markedly decreased in the HO areas of *Csnk2b*^*Osx*^ mice relative to *Csnk2b*^*fl/fl*^ mice while its mRNA levels were comparable in these HO tissues (Supplementary Fig. 14b–g). Thus, these results suggest that CK2-mediated stabilization of RUNX2 is important for the development of HO.

We next tested whether pharmacological inhibition of the CK2-HAUSP pathway can suppress HO development. Consistent with observations in *Csnk2b*-deficient COBs, treatment with a small molecule inhibitor of CK2 (i-CK2) reduced BMP2/7-induced ALP activity and mineralization and RUNX2 transcription activity (Supplementary Fig. 15a–c). Similarly, BMP2/7-induced ALP and mineralization activity and RUNX2 protein levels were decreased in osteoblasts by treatment with a small molecule inhibitor of HAUSP (i-HAUSP, HBX41108) while levels of RUNX2 mRNA were not altered (Supplementary Fig. 15d–f). Next, we tested whether pharmacological inhibition of the CK2-HAUSP pathway can suppress ectopic bone formation in mouse models of HO. One day after either muscle injury/BMP2/7 injection or burn injury/tenotomy, WT mice were treated daily with a CK2 inhibitor or a HAUSP inhibitor and HO was assessed by microCT (Fig. 7i–l). As expected, vehicle-treated mice developed heterotopic bone at the sites of injured muscle 3 weeks after muscle injury and BMP2/7 injection or 8 weeks after burn injury and Achilles tenotomy. However, when treated with either a CK2 or HAUSP inhibitor, HO was substantially reduced at the sites of injury in both mouse models. These results provide proof-of-principle that inhibition of the CK2-HAUSP-RUNX2 pathway has therapeutic potential to prevent HO. Taken together, the CK2-HAUSP-RUNX2 pathway is a key regulator of osteoblast differentiation and both orthotopic and heterotopic bone formation.

## Discussion

This study identifies that a CK2/HAUSP pathway stabilizing RUNX2 is essential for both physiologic and pathologic bone formation. Mechanistically, CK2 phosphorylates RUNX2 at T340, S354, and S387 and phosphorylated RUNX2 in turn recruits the DUB HAUSP, which stabilizes RUNX2 by counteracting ubiquitin-dependent proteasomal degradation (Fig. 5l). Previous studies have shown that ubiquitin E3 ligases, including SMURF1, SMURF2, or WWP1 induce ubiquitin-dependent proteasomal degradation of RUNX2 in osteoblasts and their deficiency in osteoblasts enhances osteoblast differentiation^15–17^. As CK2-induced phosphorylation of RUNX2 does not alter its interaction with these E3 ligases (Supplementary Fig. 11b), the CK2-HAUSP pathway is likely to operate as an independent, orthogonal mechanism for RUNX2 regulation. Lastly, this study discovers HAUSP as the DUB that stabilizes RUNX2 by suppressing ubiquitin E3 ligase-mediated ubiquitination. We note that, while we identify RUNX2 as a critical substrate for both CK2 and HAUSP in this study, both proteins are likely to have additional substrates that contribute to their regulation of osteogenesis.

HO can result in severely impaired functional recovery after injury as heterotopic bone spans across joints to limit their mobility^29–31^. Existing therapeutic approaches include bisphosphonates, external beam radiation, or nonsteroidal anti-inflammatory drugs, however each of these approaches can prevent healing of concurrent skeletal injuries and each can be complicated by either suboptimal efficacy or rare but potentially severe toxicities^32,50–52^. Given the relatively high incidence of HO, there is an urgent need to develop improved therapeutic agents. The evidence that RUNX2 expression was highly upregulated in both human and mouse HO tissues and that its inhibition prevented HO in mice implies that RUNX2 plays a role in the pathogenesis of HO^8–11^. Here, we identify a CK2-HAUSP pathway as a druggable pathway that controls RUNX2 for the treatment of HO. Development of acquired HO in two established models, muscle injury/BMP-induced HO^29,47^ and burn injury/Achilles tenotomy-induced HO^48,49^ was suppressed by *Csnk2b* deletion in osteoblasts or by treatment with either a CK2 or HAUSP inhibitor, providing proof-of-principle that inhibition of the CK2-HAUSP-RUNX2 pathway has therapeutic potential to prevent HO. Nonetheless, the long-term therapeutic outcomes and adverse effects of these inhibitors in treating HO require further investigation, as CK2 and HAUSP are involved in diverse cellular processes in addition to osteogenesis.

CK2 is a highly conserved serine/threonine kinase that is involved in various cellular processes. However, in vivo roles for CK2 in the skeleton are largely unknown. In contrast to previous in vitro studies showing that a CK2 inhibitor (CX-4945) or peptides that interfere with the interaction between CK2 and BMPRIa enhanced BMP-induced osteogenesis^27,28,53,54^, we here find genetic evidence that orthotopic and heterotopic bone formation were substantially reduced in the absence of *Csnk2b*. Furthermore, in contrast to a previous study showing inhibitory effects of CX-4945 treatment on RANKL-induced osteoclast differentiation in vitro^28^, osteoclast differentiation and bone mass are normal in mice lacking *Csnk2b* in osteoclasts (*Csnk2b*^*fl/fl*^*;Cathepsin K-cre*, Supplementary Fig. 16a), suggesting that CK2 is dispensable for osteoclast activity in vivo. Notably, unlike osteoblasts, CK2 expression was not upregulated during osteoclast differentiation (Supplementary Fig. 16b). Thus, CK2 functions as a positive regulator of osteoblast differentiation with a function specific for osteoblast-lineage cells.

The vascular development in the skeletal system is essential for physiologic bone formation and skeletal fracture repair^55–57^. Osteoblast-derived angiogenic factors, such as vascular endothelial growth factor (VEGF)^55^, erythropoietin (EPO)^58^ and SLIT3^57^, are crucial for this process. Given that CK2 plays a positive role in angiogenesis^59–61^, *Csnk2b*-sufficient and -deficient COBs were cultured under undifferentiation or osteogenic conditions and subjected for transcriptome analysis, demonstrating downregulation of a subset of the genes related to angiogenesis and vasculogenesis in the absence of *Csnk2b* (Supplementary Fig. 17). Of note, expression of key osteoblast-derived angiogenic factors, including *Vegfa, Epo*, and *Slit3* and hypoxia-inducible factor-1 alpha, a key component that mediates reciprocal interaction between angiogenesis and osteogenesis^62^, was not altered in the absence of *Csnk2b. Csnk2b*-deficient osteoblasts show reduced expression of antiangiogenic factor chemokine (C-X-C motif) ligand 9 (*Cxcl9*)^63^ (Supplementary Fig. 18). Accordingly, cell migration and tube formation of endothelial cells were relatively comparable in the culture of conditioned medium (CM) obtained from *Csnk2b*-sufficient or -deficient COBs (Supplementary Fig. 19). These results suggest that osteoblast CK2 activity is dispensable for regulation of angiogenesis and raise the possibility that CK2-mediated regulation of factors traditionally associated with angiogenesis may instead here couple to other functional processes.

## Methods

### Plasmids, antibodies, and cell culture

Plasmid for Flag/HA-*HAUSP* was deposited to Addgene by Bert Vogelstein (#16655). HA-*HAUSP* was generated by sub-cloning from this source. The construct for HA-*UBB* (Ubiquitin) was deposited to Addgene by Edward Yeh (#18712) and plasmid for His-Ubiquitin was generated by sub-cloning. All constructs encoding shRNAs were purchased from Sigma. CK2-phosphorylation sites (human NM_004348 pThr340, pSer354, pSer387) were substituted to alanine in every combination of single, double, and triple mutants on human RUNX2 cDNA^13^ using Quikchange Multi Site-Directed Mutagenesis Kit (Agilent Technologies) according to the manufacturer’s instructions.

Antibodies specific to HAUSP (sc-30164), GAPDH (sc-25778), HSP90 (sc-7947), or ubiquitin (sc-8017) were purchased from Santa Cruz Biotechnology. Antibodies specific to CSNK2A (Cell signaling, 2656), CSNK2B (Abcam, ab133576), RUNX2 (Calbiochem, PC287; Cell signaling, 12556), and USP24 (Proteintech, 13126-1-AP) were used according to manufacturers’ instructions. Recombinant human BMP2/7 (3229-BM) was purchased from R&D systems. Lastly, inhibitors of CK2 (CK2 inhibitor IV and CX-4945) were purchased from Calbiochem (218713) and Selleckchem (S2248), respectively. HAUSP inhibitor (HBX41108, 428510) was purchased Tocris Bioscience.

C3H10T1/2 and HEK293T cells were purchased from ATCC and grown in DMEM supplemented with 10% FBS, 2 mM l-glutamine, 1% penicillin/streptomycin and 1% nonessential amino acids. Human BMSCs (CD29^+^, CD44^+^, CD105^+^, CD34^−^, CD45^−^) were purchased from Cyagen Biosciences and were maintained in the growth medium (HUXMX-90011) and cultured in osteogenic medium (GUXMX-90021), chondrogenic medium (GUXMX-90041), or adipogenic medium (GUXMX-90031) according to the manufacturer’s instructions. To confirm chondrocyte differentiation, the chondrocyte pellets were harvested on days 21 and washed with phosphate-buffered saline (PBS), then stained with 1% of alcian blue solution for (Sigma, A3157). To examine adipocytes differentiation and lipid droplet formation, the differentiated cells were fixed with 4% of paraformaldehyde (PFA) for 2 h on ice, then stained with 0.3% of oil red O solution (Sigma, O0625) for 1 h on ice.

Primary *Csnk2b-* or *Hausp*-deficient osteoprogenitors (COBs) were isolated from calvaria of 5-day-old *Csnk2b*^*fl/fl*^ or *Hausp*^*fl/fl*^ neonates, respectively, using collagenase type II (50 mg/ml, Worthington, LS004176)/dispase II (100 mg/ml, Roche, 10165859001) and transduced with either lentiviruses expressing EGFP (control) or cre recombinase^13^. At 48 h after infection, COBs were treated with puromycin for selection. COBs were maintained in α-MEM medium (Gibco) containing 10% FBS (Gibco), 2 mM l-glutamine (Corning), 1% penicillin/ streptomycin (Corning), and 1% nonessential amino acids (Corning) and differentiated with ascorbic acid (200 uM, Sigma, A8960) and β-glycerophosphate (10 mM, Sigma, G9422).

### Human subjects and HO analysis

De-identified heterotopic bone samples were obtained from human patients in Yonsei University Severance Hospital, Korea under institutional review board approval (IRB No.4-2017-1223). The individuals included three patients (one male and two females, previously healthy, nonsmoking individuals; age ranging from 29 to 67 years) with pain or decreased hip movement. The specimens that show spontaneous HO radiographically and pathologically were used for histology and IHC.

### Isolation of human SSCs

Human BMA purchased from StemExpress (BMEDT010F) was incubated for 10 min at room temperature with BD Pharm Lyse hypotonic lysis buffer (BD Biosciences, 555899) for RBC lysis. Cells were washed with cold FACS buffer twice, incubated with Fc blocking buffer (BD biosciences, 564765) for 15 min at 4 °C, and treated with antibody cocktail including CD31 (1:100, BD biosciences, 560984), CD45 (1:100, BD biosciences, 560976), CD235a (1:100, BD biosciences, 561017), CD90 (1:100, BD biosciences, 566219), CD200 (1:100, BD biosciences, 564114), CD105 (1:100, BioLegend, 323217) in brilliant stain buffer (BD biosciences, 563794). After treatment with DAPI, cells were subjected for FACS analysis using a Becton Dickinson Aria II equipped with five lasers (BD biosciences). The data were analyzed using FlowJo (v.10.1). The strategy to sort skeletal progenitors is diagrammed in Supplementary Fig. 3.

### Mice

*Csnk2b*^*fl/fl*^ mice were previously reported^39^ and maintained on C57BL/6 background. *Hausp*^*fl/fl*^ mice were generated and maintained on a mixed background of 129Sv and C57BL/6J^64,65^. *Runx2* ^*fl/fl*^ mice were previously reported^5^. Transgenic mice expressing Cre recombinase under the control of the prx1 promoter (Prx1-cre)^41^ or the osterix promoter (*Osx-cre*)^43^ were purchased from Jackson laboratory and were crossed with *Csnk2b*^*fl/fl*^ mice or *Hausp*^*fl/fl*^ mice. Transgenic mice expressing Cre recombinase under the control of the Cathepsin K promoter (Ctsk-cre) were a gift from Dr Takashi Nakamura (Tokyo Dental College, Japan). Mouse genotypes were determined by PCR on tail genomic DNA; primer sequences are available upon request. Both male and female mice were used and analyzed in all experiments. Control littermates were analyzed in all experiments. All animals were used in accordance with the NIH Guide for the Care and Use of Laboratory Animals and were handled according to protocols approved by the Weill Cornell Medical College subcommittee and the University of Massachusetts Medical School on animal care (IACUC).

### MicroCT, radiography, and skeletal preparation

MicroCT was used for qualitative and quantitative assessment of trabecular and cortical bone microarchitecture and performed by an investigator blinded to the genotypes of the animals under analysis. Femurs excised from the indicated mice were scanned using a microCT 35 (Scanco Medical) with a spatial resolution of 7 μm. For trabecular bone analysis of the distal femur, an upper 2.1 mm region beginning 280 μm proximal to the growth plate was contoured. For cortical bone analysis of femur and tibia, a midshaft region of 0.6 mm in length was used. MicroCT scans of skulls, kidneys, and HO in muscle/achilles tendon were performed using isotropic voxel sizes of 12 μm. 3D-reconstruction images were obtained from contoured 2D images by methods based on distance transformation of the binarized images. All images presented are representative of the respective genotypes (*n* > 5).

Radiographic images of clavicles were taken by the Faxitron Specimen Radiography System Model Mx-20 at 26 kV for 20 s.

Skeletons were prepared for analysis of gross morphology using the method of McLeod^66^. Briefly, mice were sacrificed, skinned, eviscerated, and fixed in 95% ethanol for a day. Then, skeletons were stained by alizarin red S and alcian blue (Sigma, A3157) solutions and sequentially cleared in 1% potassium hydroxide. All images presented are representative of the respective genotypes (*n* > 5).

### Histology, histomorphometry, and IHC

For histological analysis, hindlimbs were dissected from the mice, fixed in 10% neutral buffered formalin for 2 days, and decalcified by daily changes of 15% tetrasodium EDTA for 1–2 weeks. Tissues were dehydrated by passage through an ethanol series, cleared twice in xylene, embedded in paraffin, and sectioned at 7-μm thickness along the coronal plate from anterior to posterior. Decalcified femoral sections were stained with hematoxylin and eosin (H&E), safranin O, or TRAP.

For histomorphometric analysis, 25 mg/kg calcein (Sigma, C0875) and 50 mg/kg alizarin-3-methyliminodiacetic acid (Sigma, A3882) dissolved in 2% sodium bicarbonate solution were subcutaneously injected into mice at 5 day-interval. After 2 day-fixation in 10% neutral buffered formalin, undecalcified femur samples were embedded in methylmethacrylate. Proximal metaphysis was sectioned longitudinally (5 μm) and stained with McNeal’s trichrome for osteoid assessment, toluidine blue for osteoblasts, and TRAP for osteoclasts^67^. A region of interest is defined and bone formation rate/bone surface (BFR/BS), MAR, bone area (B.Ar), and osteoclast number/bone parameter were measured using a Nikon Optiphot 2 microscope interfaced to a semiautomatic analysis system (Osteometrics). Measurements are taken on two sections/sample (separated by ~25 μm) and summed prior to normalization to obtain a single measure/sample in accordance with ASBMR standards^68^. This methodology has undergone extensive quality control and validation and the results will be assessed by a research specialist in a blinded fashion.

For IHC, paraffin sections were dewaxed and stained as following procedure using the Discovery XT automated IHC stainer (Ventana Medical Systems, Inc., Tucson, AZ, USA). CC1 standard (pH 8.4 buffer contained Tris/Borate/EDTA) and inhibitor D (3% H2O2, Endogenous peroxidase) were used for antigen retrieval and blocking, respectively. Sections were incubated with antibodies specific to HAUSP (1:50, ABclonal, A13564), RUNX2 (1:100, MBL, D130-3), Casein Kinase 2 beta (1:100, Abcam, ab133576), Casein Kinase 2 alpha (1:100, Abcam, ab70774) for 40 min at 37 °C, and a secondary antibody of Universal secondary antibodies for 20 min at 37 °C. Subsequently, they were incubated in SA-HRP D for 16 min at 37 °C and then DAB + H_2_O_2_ substrate for 8 min followed by hematoxylin and bluing reagent counterstain at 37 °C. Reaction buffer (pH 7.6 Tris buffer) was used as washing solution. The stained samples were visualized using an Aperio virtual microscope (Leica Microsystems, USA) and images of the sample were analyzed by the Aperio image scope program (ver. 12.3.2.8013, Leica Microsystems, USA). For immunofluorescence, antibodies for CSNK2B (1:100, Abcam, ab76025), RUNX2 (1:50, Santacruz, sc-390351), HAUSP (1:50, Abclonal, A13564), and COL1A1 (1:50, Abclonal, A1352) were used as primary antibodies and Alexa Fluor 488 (1:400, Thermo, A21206) and Alexa Fluor 594 (1:400, Thermo, A11032) were used as secondary antibodies as manufacturer’s descriptions.

### Kidney transplantation of SSCs

E17.5 *Csnk2b*^*fl/fl*^ and *Csnk2b*^*Prx1*^ embryonic limbs were dissociated by mechanical and enzymatic digestion (1 mg/ml of Collagenase P (Roche, 11213857), 2 mg/ml of Dispase II (Roche, 10165859001), 1 mg/ml of Hyaluronidase (Sigma, H3506) and 10000 unit/ml of DNase I (Roche, 4716728001)) for 1 h at 37 °C under gentle agitation. After digestion, cells were passed through 40 μm cell strainer and washed with FACS buffer (cold PBS (pH 7.2) containing 0.5% BSA (Fraction V) and 1 mM EDTA). The cells were subsequently blocked with purified rat anti-mouse CD16/CD32 (Biolegend, 553141) for 1 h on ice and stained with Biotin-conjugated CD45 (1:200, ebioscience, 13-0451-81), Tie2 (1:200, ebioscience, 13-5987-81) and Ter119 (1:200, ebioscience, 13-5921-81) with BV421-conjuated streptavidin (1:500, Biolegend, 405226), PE-conjugated CD51 (1:200, ebioscience, 12-0512-81), BV605-conjugated Thy1.1–2 (1:200, Biolegend, 202537/140317), APC-conjugated CD200 (1:100, Biolegend, 123809), FITC-conjugated LY51 (1:50, Biolegend, 108305) and PE/Cy7-conjugated CD105 (1:200, Biolegend, 120409) for 45 min on ice. After washing three times, cells were resuspended in cold PBS (pH 7.2) with 1 mM EDTA and 1 µg/ml DAPI and sorted with a FACSAria II SORP cell sorter (Becton Dickinson) with exclusion of DAPI^+^ cells and doublets. The strategy to sort skeletal progenitors is diagrammed in Supplementary Fig. 6. The sorted mouse SSCs (10,000 cells/mouse) were mixed with 20 μl of matrigel (Corning, 354230) and injected underneath the renal capsules of 8-week-old anesthetized male mice (C57BL/6). Six to eight weeks after transplantation, renal samples were fixed in 4% of PFA and their bone mass was assessed by microCT. For histology, they were embedded in methylmethacrylate and stained with H&E and Von Kossa.

### Cell proliferation assay

Cell proliferation was examined using bromodeoxyuridine (BrdU) incorporation assay (Abcam, ab126556) according to manufacturer’s instructions.

### Mouse models of acquired HO

For muscle injury/BMP-induced HO model^29,47^, blunt muscle trauma was induced by dropping an aluminum ball into mouse adductor muscle right next to femur and a mixture of recombinant BMP2/7 (1 μg) and matrigel (20 μl) was injected into the injured area. Subcutaneous injection of buprenorphine was provided for analgesia. Mice were euthanized and HO was assessed by microCT and histology at 3 weeks post injury.

For burn injury/Achilles tenotomy-induce HO model^48,49^, ~30% total body surface area was exposed to 60 °C heated aluminum block for 18 s, a tegaderm dressing was applied to burn site and sterile saline solution, and buprenorphine were subcutaneously administrated. Subsequently, mice received an Achilles tenotomy on the right leg. Mice were euthanized and HO was assessed by microCT and histology at 8 weeks post injury.

To test the therapeutic potential of CK2 or HAUSP inhibitor to prevent HO, 3-month-old wild-type male mice (C57BL/6) were subjected to the HO models above and randomly divided into different experimental groups. One day after HO injury, mice were daily treated with DMSO (vehicle control), CK2 inhibitor (2.5 mg/kg) or HAUSP inhibitor (2 mg/kg) via intraperitoneal (i.p.) injection for 3 weeks (muscle injury/BMP-induced HO) or for 8 weeks (burn injury/Achilles tenotomy-induced HO). HO tissue was harvested, fixed in 4% PFA, and HO was assessed by microCT.

### Osteoblast differentiation analysis

For ALP staining, osteoblasts were fixed with 10% neutral formalin buffer and stained with the solution containing Fast Blue (Sigma, FBS25) and Naphthol AS-MX (Sigma, 855). Alternatively, osteoblasts were incubated with tenfold diluted Alamar Blue solution (Invitrogen, DAL1100) for cell proliferation. Subsequently, cells were washed and incubated with a solution containing 6.5 mM Na_2_CO_3_, 18.5 mM NaHCO_3_, 2 mM MgCl_2_, and phosphatase substrate (Sigma, S0942), and ALP activity was measured by spectrometer (Biorad).

To assess extracellular matrix mineralization in mature osteoblasts, cells were washed twice with PBS and fixed in 70% EtOH for 15 min at room temperature. Fixed cells were washed twice with distilled water and then stained with a 2% alizarin red solution (Sigma, A5533) for 5 min. Cells were then washed three times with distilled water and examined for the presence of calcium deposits. Mineralization was quantified by the acetic acid extraction method^69^.

### Osteoclast differentiation

For preparation of bone marrow monocytes, femur was dissected from 8-week-old mice, and bone marrow cells collected by flushing were plated overnight in α–MEM with 10% FBS. Non-adherent cells were collected and seeded on a 100 mm dish with M-CSF (20 ng/ml, R&D systems, 416-ML-010). After 48 h, adherent cells were plated and cultured in osteoclast differentiation condition up to 6 days. For osteoclastogenesis, M-CSF (20 ng/ml) and RANKL (10 ng/ml, R&D systems, 462-TEC-010) were added in the growth medium.

### RT-PCR, immunoprecipitation, and immunoblotting

To prepare RNA samples from the mouse limbs, the hindlimbs were dissected and skin/muscle tissues were removed. The remaining tibias/femurs were chopped and homogenized. For extraction RNA from mouse HO models, HO-formed muscle, and HO-formed achilles tendon were dissected and homogenized mechanically. Total RNAs were purified using QIAzol (QIAGEN) and cDNA was synthesized using the High-Capacity cDNA Reverse Transcription Kit from Applied Biosystems. Quantitative RT-PCR was performed using SYBR® Green PCR Master Mix (Applied Biosystems) with QuantStudio (TM) 6 Flex System (Applied Biosystems). The primers used for PCR are described in the Supplementary Table 1.

To examine endogenous interaction between RUNX2 and CK2 or USP24, cell lysates were obtained from mouse COBs and prepared in lysis buffer (50 mM Tris-HCl (pH 7.4), 150 mM NaCl, 1% Triton X-100, 1 mM EDTA, 1 mM EGTA, 50 mM NaF, 1 mM Na_3_VO_4_, 1 mM PMSF, and protease inhibitor cocktail (Sigma)). Cell lysates were incubated with IgG control or anti-RUNX2 and immunoprecipitated with protein G-conjugated agarose. For exogenous interaction, HEK293T cells were transfected with the indicated DNA constructs using the Effectene transfection reagent (Qiagen) and 48 h later, cell lysates were prepared in lysis buffer. Proteins from cell lysates were immunoprecipitated with Flag-conjugated (Sigma, A2220) or HA-conjugated (Santa Cruz, sc-7392 AC) were subjected to SDS-PAGE, transferred to Immobilon-P membranes (Millipore), immunoblotted with the indicated antibodies, and developed with ECL (Thermoscientific). Immunoblotting with antibodies specific to GAPDH or HSP90 was used as a loading control.

### Luciferase assay

RUNX2-responsive reporter gene (OG2-Luc) and *Renilla* luciferase vector (Promega) were transfected into C3H10T1/2 cells or human BMSCs using the Effectene transfection reagent (Qiagen). After 48 h, dual luciferase assay was performed according to the manufacturer’s protocol (Promega) and OG2 luciferase activity was normalized to *Renilla*.

### In vitro kinase assay and phospho-mass spectrometry

Two hundred nanograms of recombinant CK2 (NEB, P6010) and 300 ng of recombinant RUNX2 (Origene, TP760214) were incubated in kinase buffer (20 mM HEPES, pH 7.5, 20 mM MgCl_2_, 1 mM EDTA, 2 mM NaF, 2 mM-glycerophosphate, 1 mM DTT, 10 μM ATP) containing 10 μCi of γ32P-ATP (PerkinElmer) for 15 min at 30 °C. The phosphorylated proteins were visualized by autoradiography.

For phospho-mass spectrometry, 1 μg of recombinant CK2 and RUNX2 were incubated in kinase buffer containing cold ATP for 30 min at 30 °C, subjected to SDS-PAGE, and stained with Coomassie blue. Phosphorylated RUNX2 protein at the band size of 55 kDa was excised and subjected to phospho-mass spectrometry.

### Mass spectrometry and IPA

Plasmids expressing Flag-RUNX2-WT (1 μg) or Flag-RUNX2-Tri-A mutant (2 μg) were transfected into HEK293T cells using the Effectene transfection reagent. After 48 h, its binding proteins were co-immunoprecipitated with Flag-conjugated agarose, eluted with excessive amounts of Flag-peptide, and the eluates were subjected to mass spectrometry. Due to low expression levels of Flag-RUNX2 (Tri-A), double amounts of the plasmid encoding Flag-RUNX2 (Tri-A) relative to Flag-RUNX2 (WT) were transfected to HEK293T cells in order to express equivalent protein levels of Flag-RUNX2. Expression intensity of peptides obtained from mass spectrometry was applied to the IPA (Qiagen) to identify proteins that differentially bind to RUNX2-WT vs. RUNX2-Tri-A mutant. Briefly, expression intensity of peptides from mass spectrometry was first uploaded into IPA system for core analysis and then overlaid with the dataset based on the literature and compiled in the ingenuity pathway knowledge base. IPA was performed to identify canonical pathways that are most significant to mass spectrometry outcomes.

### Deubiquitination assay

To test the ability of HAUSP to deubiquitinate ubiquitinated RUNX2, plasmids expressing Flag-RUNX2-WT or Flag-RUNX2-Tri-A mutant were transfected into HEK293T cells along with His-ubiquitin-expressing plasmid in the absence or presence of HAUSP-expressing plasmid. After 48 h, cells were treated with 10 μM MG132 (EMD-Millipore, 474790) for 6 h, lysed and sonicated in denaturation buffer (8 M urea, 50 mM tris pH 8.0, 1.0% triton X-100, 10 mM imidazol, 10 mM β-mercaptoethanol), and immunoprecipitated with Ni-NTA beads. Immunoprecipitates were subjected to SDS-PAGE and immunoblotted with anti-RUNX2 antibody.

A cell-free deubiquitination assay was also performed using the purified ubiquitinated RUNX2 and recombinant HAUSP. Ubiquitinated RUNX2 proteins were purified from HEK293T cells expressing Flag-RUNX2 and HA-ubiquitin by immunoprecipitating with Flag-conjugated agarose. These RUNX2 proteins were subsequently incubated with different amounts of recombinant HAUSP (Boston Biochem, E-519) in deubiquitination buffer (50 mM Tris-HCl (pH 8.0), 50 mM NaCl, 1 mM EDTA, 10 mM DTT, 5% glycerol) at 37 °C for 3 h^70^. Immunoblotting was performed to measure ubiquitination levels.

### RNA sequencing and analysis

Adapters are removed and reads are aligned to mouse genome GRCm38 by using STAR aligner (ver. 2.3.0e)^71^ using default parameters and resulting bam files (mapped reads) were sorted and indexed using SAMTools^72^. Gene counts were obtained by HTSeq-Count^73^ to sorted bam files, and only unique-mapping reads were used. For dendrogram (heat map) generation, 3850 total genes were clustered into four groups and the gene sets in these groups are described in Supplementary Table 2. Genes without any expression counts in any sample were discarded. The DESeq2 (ver. 1.4.5) in R package^74^ was employed to normalize gene count data, and then detect differentially expressed genes (DEG) between each groups with (FDR < 0.005 and absolute log2 fold change >1.5). Functional enrichment analysis was performed on DEG with DAVID (ver. 6.7)^75^ and biological process GO terms with enrichment *p* < 0.05 were selected as overrepresented functions. Summary statistics of the fold change analysis in each of the GO categories is described in Supplementary Table 3.

### Collection of CM from WT and *Csnk2b* KO COBs

CM from primary WT and *Csnk2b*-deficient COBs was collected at day 0 (undifferentiated COBs) and day 7 (differentiated osteoblasts) after osteogenic induction. CM for each group was collected after three washes with PBS to remove serum and incubated with serum free α-MEM for 24 h. After incubation, each CM sample was collected and filtered with 0.45 μm syringe filter to remove cellular debris.

### Endothelial cell culture and functional assays

Mouse bone marrow-derived Endothelial progenitor outgrowth cells (EPOCs) were purchased from BioChain (Z7030031) and maintained in growth medium (BioChain, Z7030035). Cell migration was performed using chemotaxis chamber (Biovision, K906) as manufacturer’s instructions. Briefly, basal medium or CM or FGF-2 (R&D systems, 3139-FB-025) was placed in the bottom wells of the chamber and the upper chamber was loaded with 10,000 cells/well. After incubation for 6 h at 37 °C, the migrated cells were analyzed and quantified.

For tube formation, EPOCs (100,000 cells/well) were plated into 48-well plates coated with growth factor-reduced matrigel (Corning, 354230) and incubated for 24 h at 37 °C in indicated conditions. Total tube length was measured by counting a random field per well with microscopy (5–7 wells per each group).

### Statistics and reproducibility

All experiments were carried out at least two or three times, for IHC, immunofluorescence staining, histological staining, skeletal preparation, flow cytometry, and immunoblotting, representative images are shown. All data are shown as the mean ± Standard Deviation (SD). We first performed the Shapiro–Wilk normality test for checking normal distributions of the groups. If normality tests passed, two-tailed, unpaired Student’s *t* test and if normality tests failed, and Mann–Whitney tests were used for the comparisons between two groups. For the comparisons of three or four groups, we used one-way ANOVA if normality tests passed, followed by Tukey’s multiple comparison test for all pairs of groups. The GraphPad PRISM software (ver.8.3.0, La Jolla, CA) was used for statistical analysis. *P* < 0.05 was considered statistically significant.

### Reporting summary

Further information on research design is available in the Nature Research Reporting Summary linked to this article.

## Supplementary information

Supplementary Information

Reporting Summary

## Data Availability

Data supporting the findings of this manuscript are available from the corresponding author upon reasonable request.

## Source Data

Source Data
