# Supplementary material for: A RUNX2 stabilization pathway mediates physiologic and pathologic bone formation
Source: Nat Commun. 2020 May 8;11:2289. doi: 10.1038/s41467-020-16038-6 (PMC7210266; doi:10.1038/s41467-020-16038-6)
Supplement: Supplementary file 1 — Supplementary Information [file 41467_2020_16038_MOESM1_ESM.pdf]

Supplementary Information

A novel RUNX2 stabilization pathway mediates physiologic and pathologic bone formation

Kim et al.

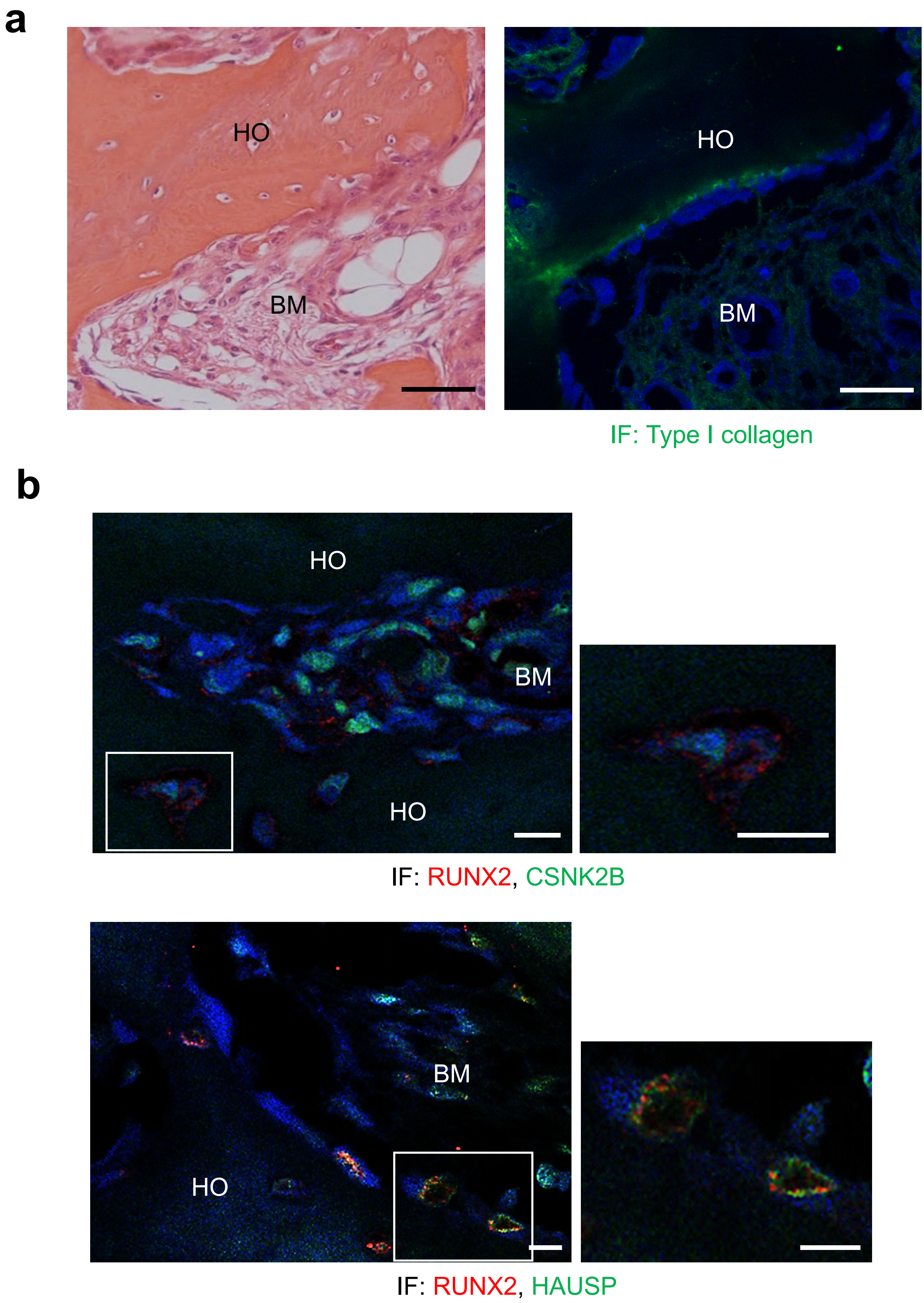

Supplementary Fig. 1: Histological analysis of human HO tissues.

(**a**) Human HO tissues were stained for H&E (left) and immunostained for the type I collagen (right). Scale bar, 25 μm. (**b**) Immunofluorescence analysis demonstrating co-localization of RUNX2 with either CSNK2B or HAUSP in human HO tissues. Scale bar, 10 μm. HO, heterotopic bone; BM, bone marrow. Data are representative of three independent experiments.

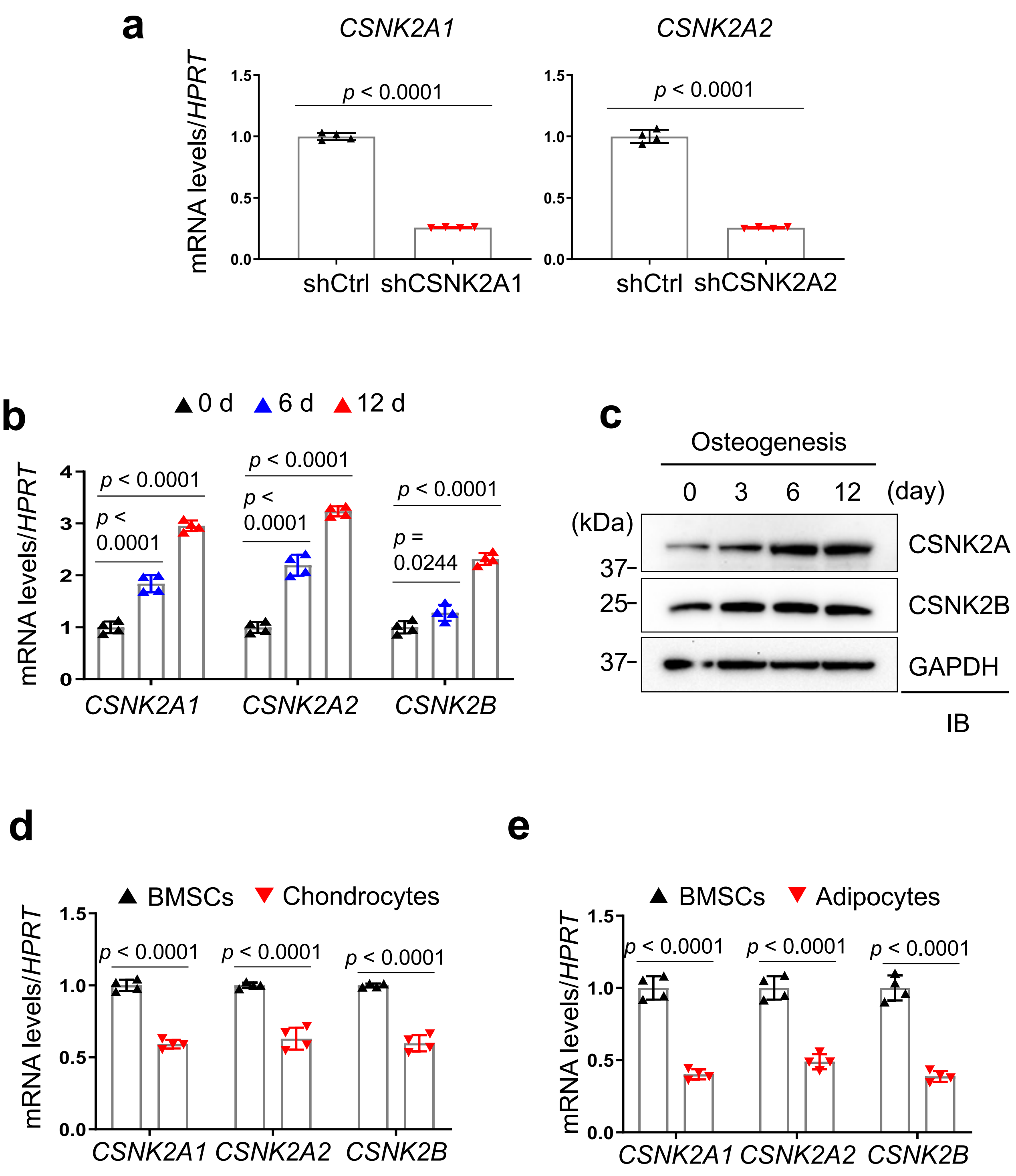

Supplementary Fig. 2: Expression of CK2 subunits in human BMSCs.

(**a**) mRNA levels of CK2 subunits in human BMSCs expressing control shRNA, or shRNAs targeting CSNK2A1 or A2 (n = 4). (**b, c**) mRNA (**b**) and protein levels (**c**) of CK2 subunits in human BMSCs cultured under osteogenic conditions (**b**, n = 4). (**d, e**) mRNA levels of CK2 subunits in human BMSCs cultured under chondrogenic conditions for 21 days (**d**) or adipogenic conditions for 12 days (**e**) (n = 4). Data are representative of three independent experiments. A two-tailed unpaired Student's t-test for comparing two groups (**a, d, e**) and ordinary one-way ANOVA with Dunnett's multiple comparisons test (**b;** error bars, SD of biological replicates). n represents biologically independent samples.

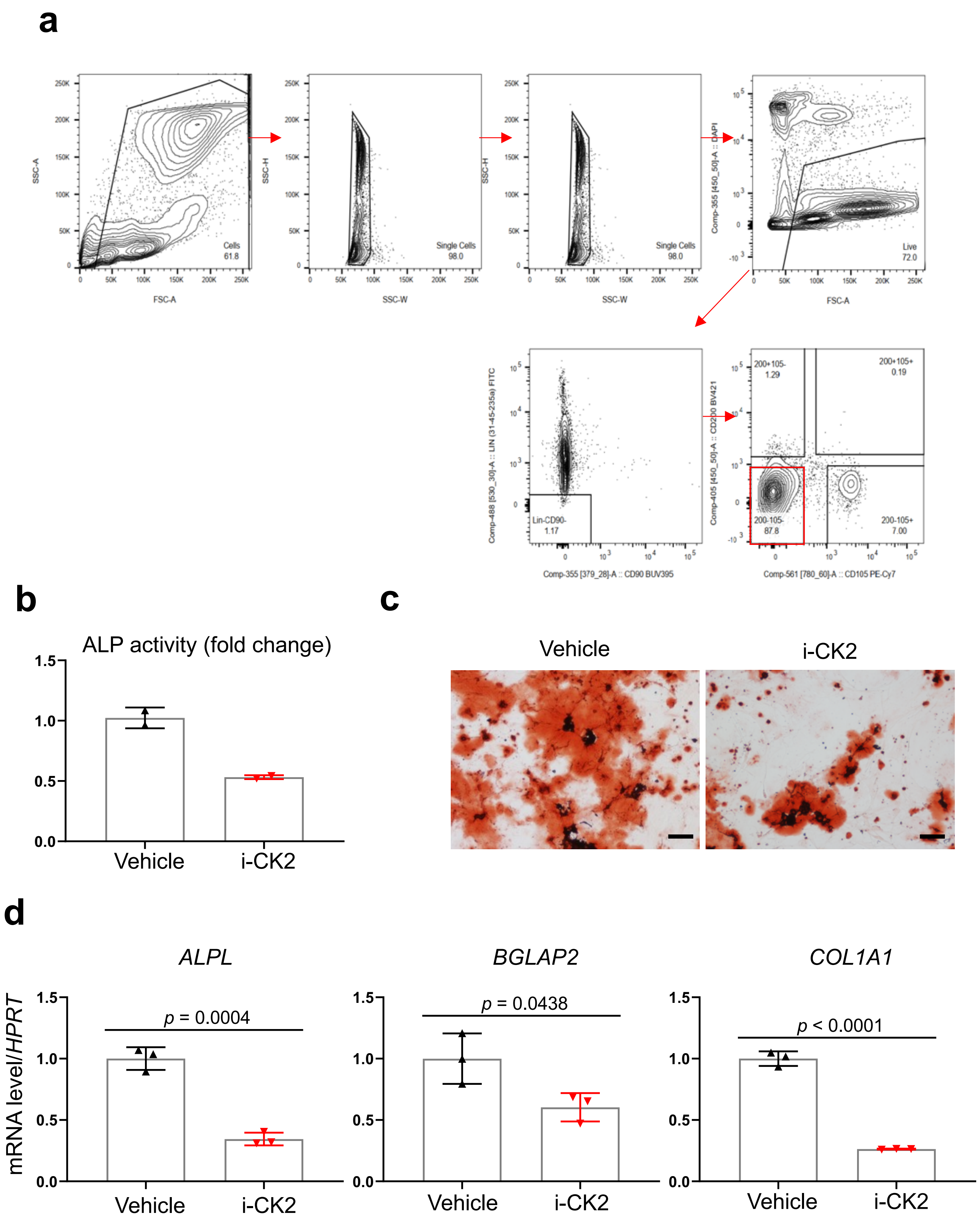

Supplementary Fig. 3: CK2 is required for osteogenesis of human skeletal Lin$^-$CD90$^-$CD200$^+$CD105$^-$cells.

(**a**) Representative flow cytometry dot plot showing frequency of a putative human skeletal stem cell population (CD45$^-$CD31$^-$CD235a$^-$ (Lin$^-$)CD90$^-$CD200$^+$CD105$^-$ cells, SSCs, red box) isolated from human bone marrow aspirates. (**b-d**) Putative human SSCs were cultured under osteogenic conditions in the presence of DMSO (vehicle) or 1 μM CK2 inhibitor (i-CK2). ALP activity (**b**) and mineralization of osteoblasts (**c**) were determined at day 7 and at day 14, respectively. Osteogenic gene expression was examined by RT-PCR at 14 days after osteogenic differentiation (**d**). Scale bar, 200 μm (**c**). (**b**, *n* = 2; **d**, *n* = 3 biologically independent samples). Data are representative of two independent experiments. A two-tailed unpaired Student's t-test for comparing two groups (**d;** error bars, SD of biological replicates).

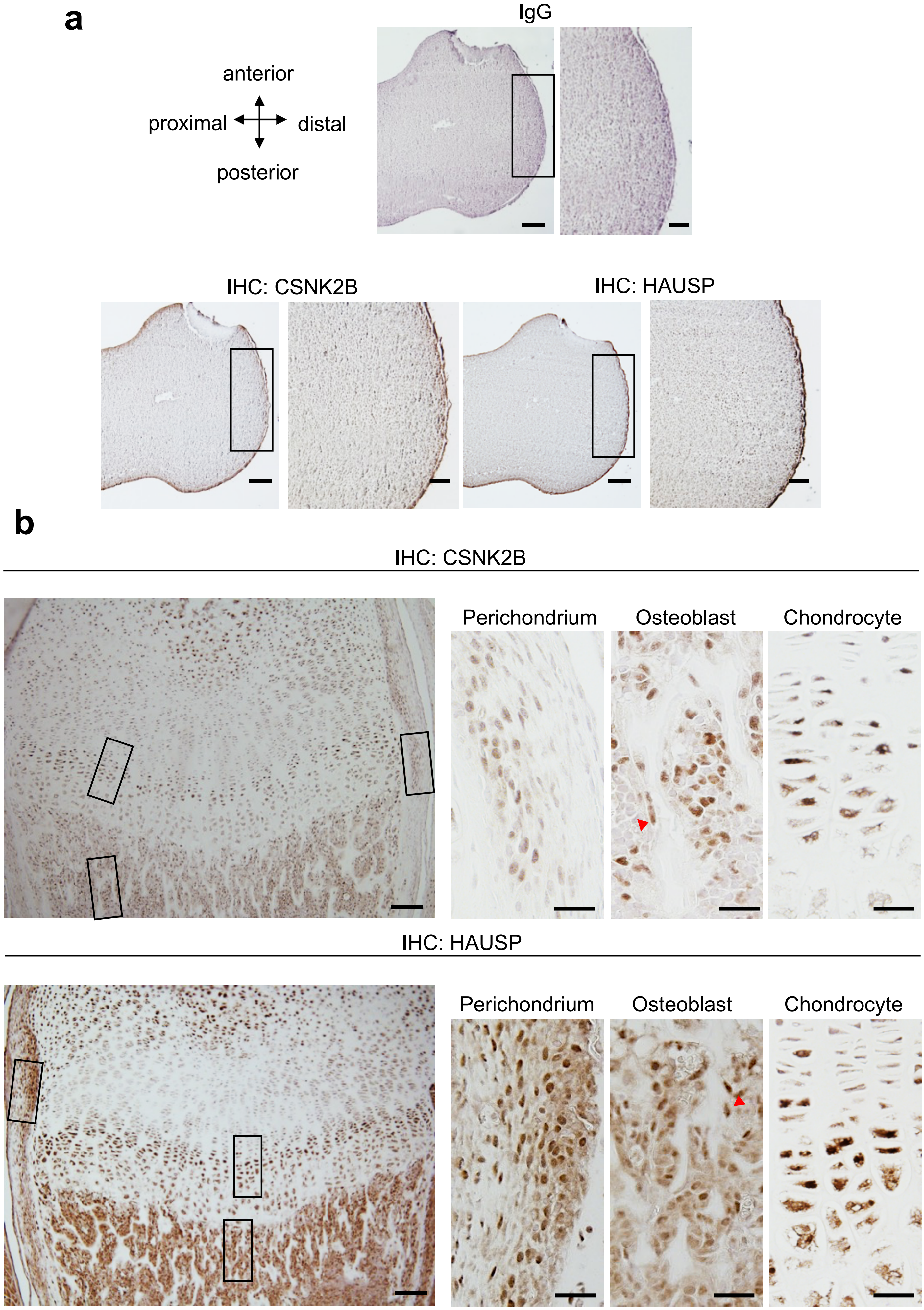

Supplementary Fig. 4: Expression of CSNK2B and HAUSP in the skeleton.

(**a**) Immunohistochemistry for CSNK2B or HAUSP in the limb bud at embryonic day 11.5. IgG was used for negative staining (top). Scale bars, 100 μm (left); 50 μm (right, enlarged one). (**b**) Immunohistochemistry for CSNK2B or HAUSP in the femur at postnatal day 10. Expression of CSNK2B and HAUSP in progenitor cells residing in the perichondrium, osteoblasts in the trabecular bone and chondrocytes in the growth plate was observed. Arrows indicate bone-lining osteoblasts. Scale bars, 100 μm (left); 50 μm (right, enlarged one). Data are representative of three independent experiments.

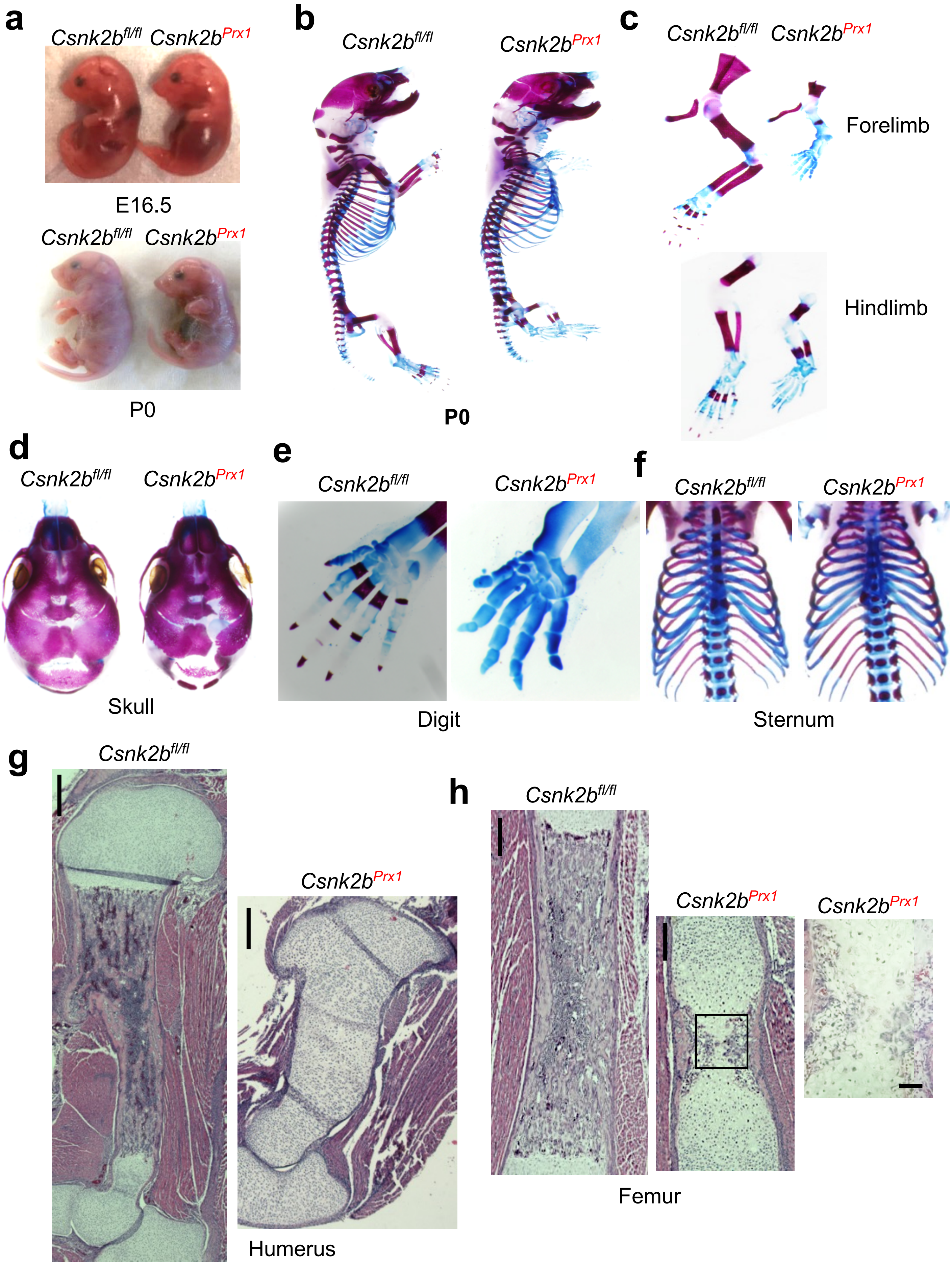

Supplementary Fig. 5: Characterization of skeletal phenotypes in *Csnk2b*^{Prx1} mice.

(**a**) Representative photographs showing E16.5 *Csnk2b*$^{fl/fl}$ and *Csnk2b*^{Prx1}embryos (top) and P0 *Csnk2b*$^{fl/fl}$ and *Csnk2b*^{Prx1} neonates (bottom). (**b-f**) Alizarin red/alcian blue staining of skeletal preparations of P0 *Csnk2b*$^{fl/fl}$ and *Csnk2b*^{Prx1} neonates. Whole bodies (**b**); forelimbs (**c**, top) and hindlimbs (**c**, bottom); calvaria (**d**); digits (**e**); sternums (**f**). (**g, h**) H&E-stained longitudinal sections of humeri (**g**) and femurs (**h**) of P0 *Csnk2b*$^{fl/fl}$ and *Csnk2b*^{Prx1} neonates. Scale bars, 250 μm (left) and 50 μm (right, enlarged one). Data in **b-h** are representative of three independent experiments.

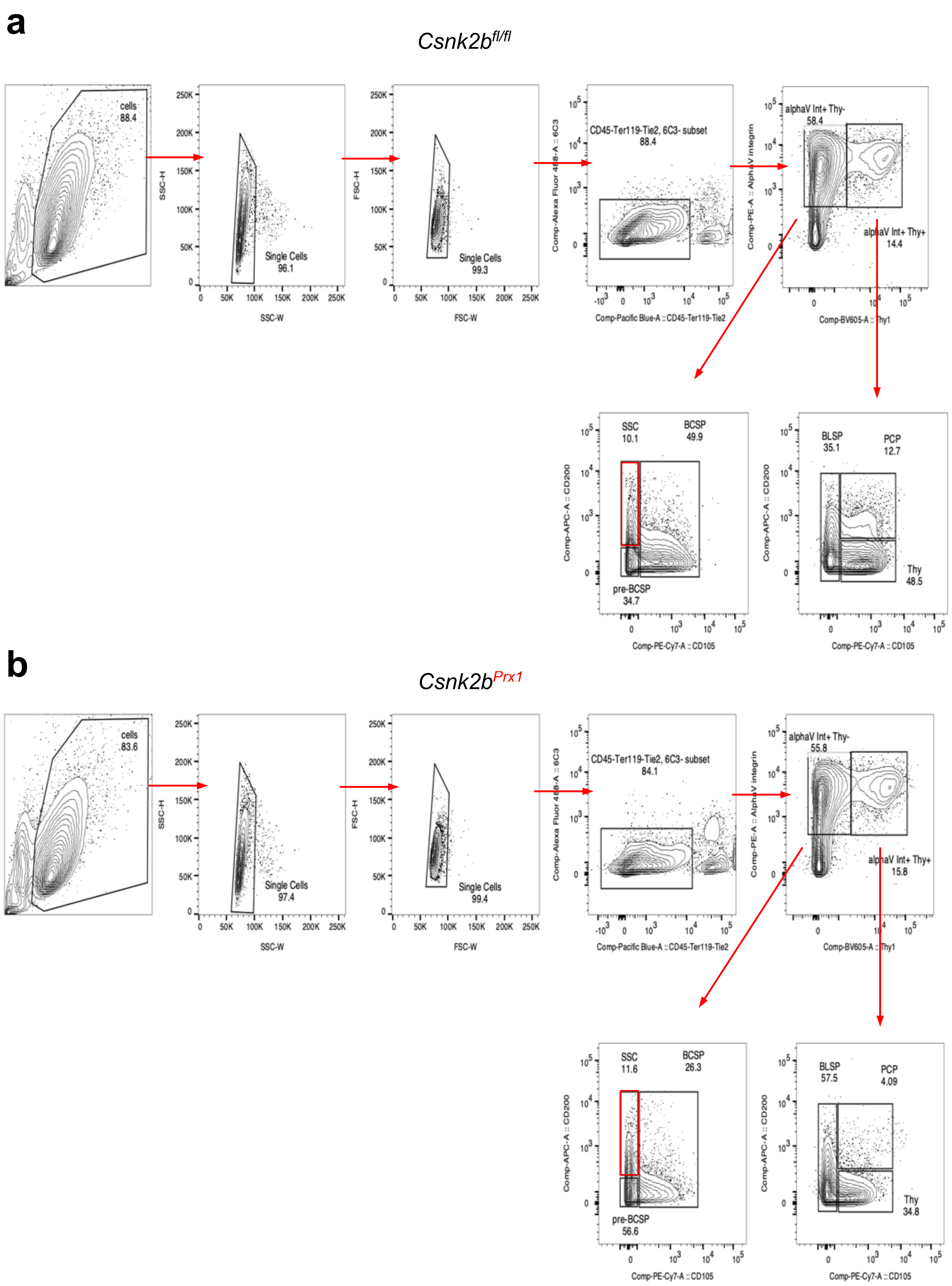

Supplementary Fig. 6: Isolation of mouse embryonic SSCs using flow cytometry.

Representative flow cytometry dot plot showing frequency of mouse skeletal stem cells

(SSCs, red box) isolated from E17.5 *Csnk2b*$^{fl/fl}$ (**a**) and *Csnk2b*^{Prx1} (**b**) embryonic limbs. BCSP (bone, cartilage, and stromal progenitor, CD45$^-$Ter-119$^-$Tie2$^-$AlphaV$^+$Thy$^+$6C3$^-$CD105$^+$); Pre-BCSP (pre-bone cartilage and stromal progenitor, CD45$^-$Ter-119$^-$Tie2$^-$AlphaV$^+$Thy$^-$6C3$^-$CD105$^-$CD200$^-$); PCP (pro-chondrogenic progenitors, CD45$^-$Ter-119$^-$Tie2$^-$AlphaV$^+$Thy$^+$6C3$^-$CD105$^+$CD200$^+$); Thy (the Thy subpopulation, CD45$^-$Ter-119$^-$Tie2$^-$AlphaV$^+$Thy$^+$6C3$^-$CD105$^+$); BLSP (B cell lymphocyte stromal progenitor, CD45$^-$Ter-119$^-$Tie2$^-$AlphaV$^+$Thy$^+$6C3$^-$CD105$^-$); SSC (skeletal stem cell, CD45$^-$Ter-119$^-$ Tie2$^-$AlphaV$^+$Thy$^+$6C3$^-$CD105$^-$ CD200$^+$). Data are representative of three independent experiments.

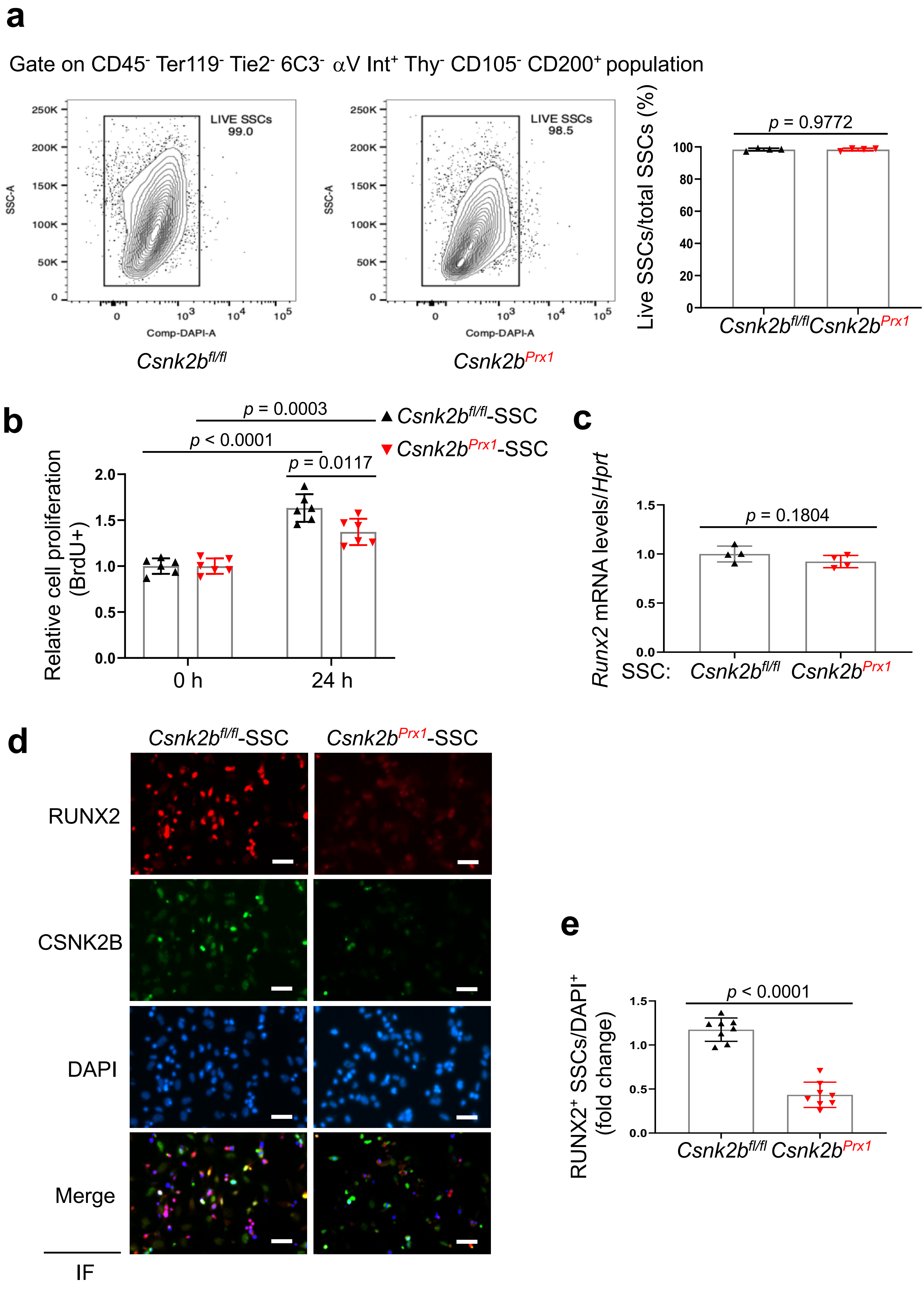

Supplementary Fig. 7: Characterization of *Csnk2b^fl/fl^* and *Csnk2b^Prx1^* embryonic SSCs.

(**a**) Cell death was examined using DAPI staining and DAPI-negative cells represent live SSCs. A representative flow cytometry dot plot is shown (left) and the percentage of live SSCs among total SSCs was analyzed (right). (n = 4). (**b**) Cell proliferation was examined using BrdU incorporation. SSCs isolated from E17.5 *Csnk2b$^{fl/fl}$* and *Csnk2b^{Prx1}* embryos were labeled with BrdU in culture for 24 h, BrdU-positive cells were examined. (n = 6). (**c**) *Runx2* mRNA levels in SSCs isolated from E17.5 *Csnk2b$^{fl/fl}$* and *Csnk2b^{Prx1}* embryos. (n = 4). (**d, e**) SSCs isolated from E17.5 *Csnk2b$^{fl/fl}$* and *Csnk2b^{Prx1}* embryos were immunostained for RUNX2 or CSNK2B. Representative images (left) and relative quantification of RUNX2-postivie cells (right) are displayed. Scale bar, 100 μm (**d**). (n = 8). Data are representative of three independent experiments. A two-tailed unpaired Student's t-test for comparing two groups (**a, b, c, e;** error bars, SD of biological replicates). n represents biologically independent samples.

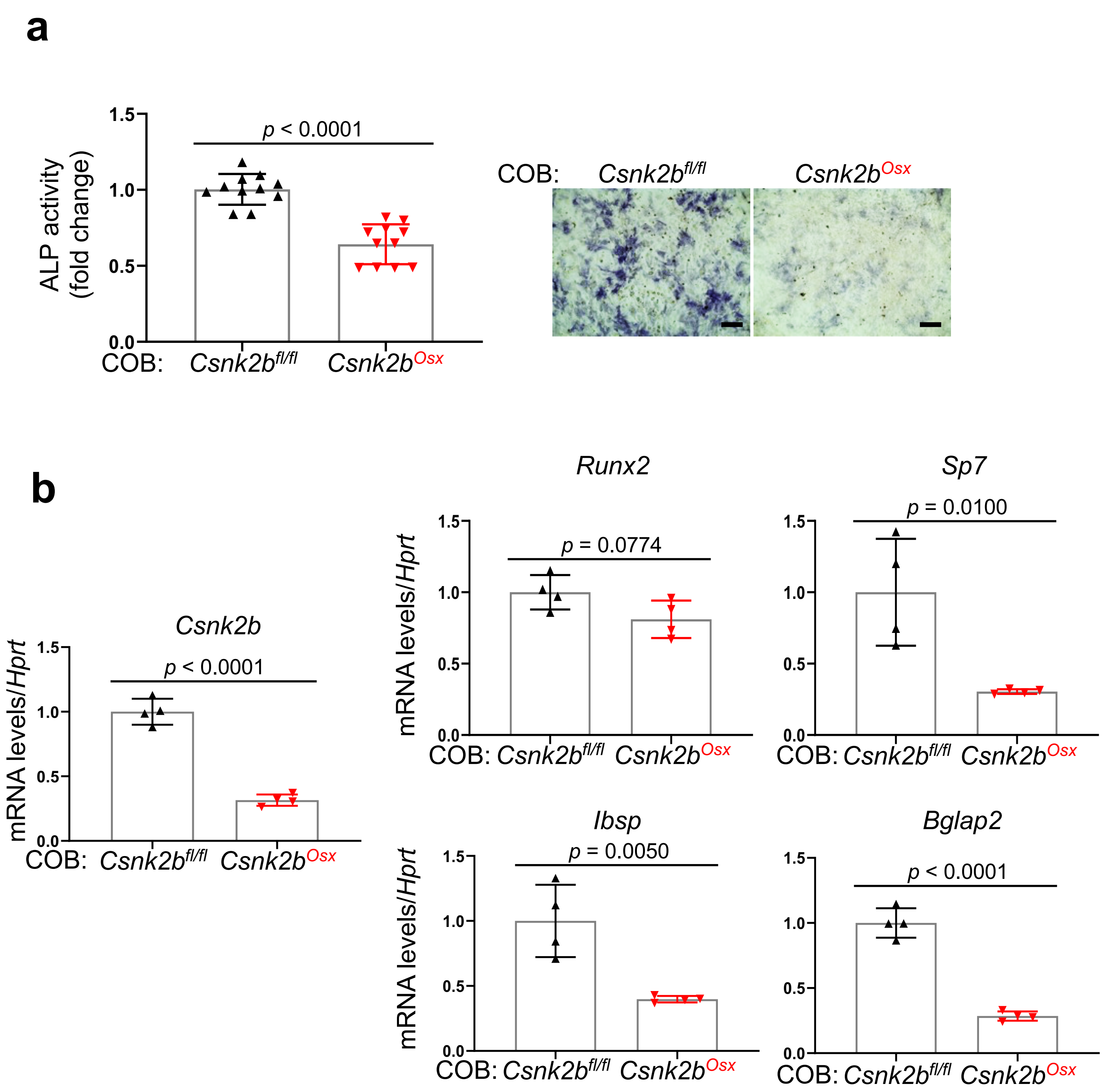

Supplementary Fig. 8: Decreased osteogenic potential of *Csnk2b*^{Osx} COBs.

COBs isolated from P5 *Csnk2b*$^{fl/fl}$ and *Csnk2b*^{Osx} pups were cultured under osteogenic conditions. ALP activity (**a**, left) and staining (**a**, right) were performed at 7 days and mRNA levels of osteogenic genes (**b**) were analyzed by RT-PCR at 12 days after the culture. Scale bar, 200 μm (**a**). (**a**, *n* = 11; **b**, *n* = 4 biologically independent samples.). Data are representative of three independent experiments. A two-tailed unpaired Student's t-test for comparing two groups (**a, b;** error bars, SD of biological replicates).

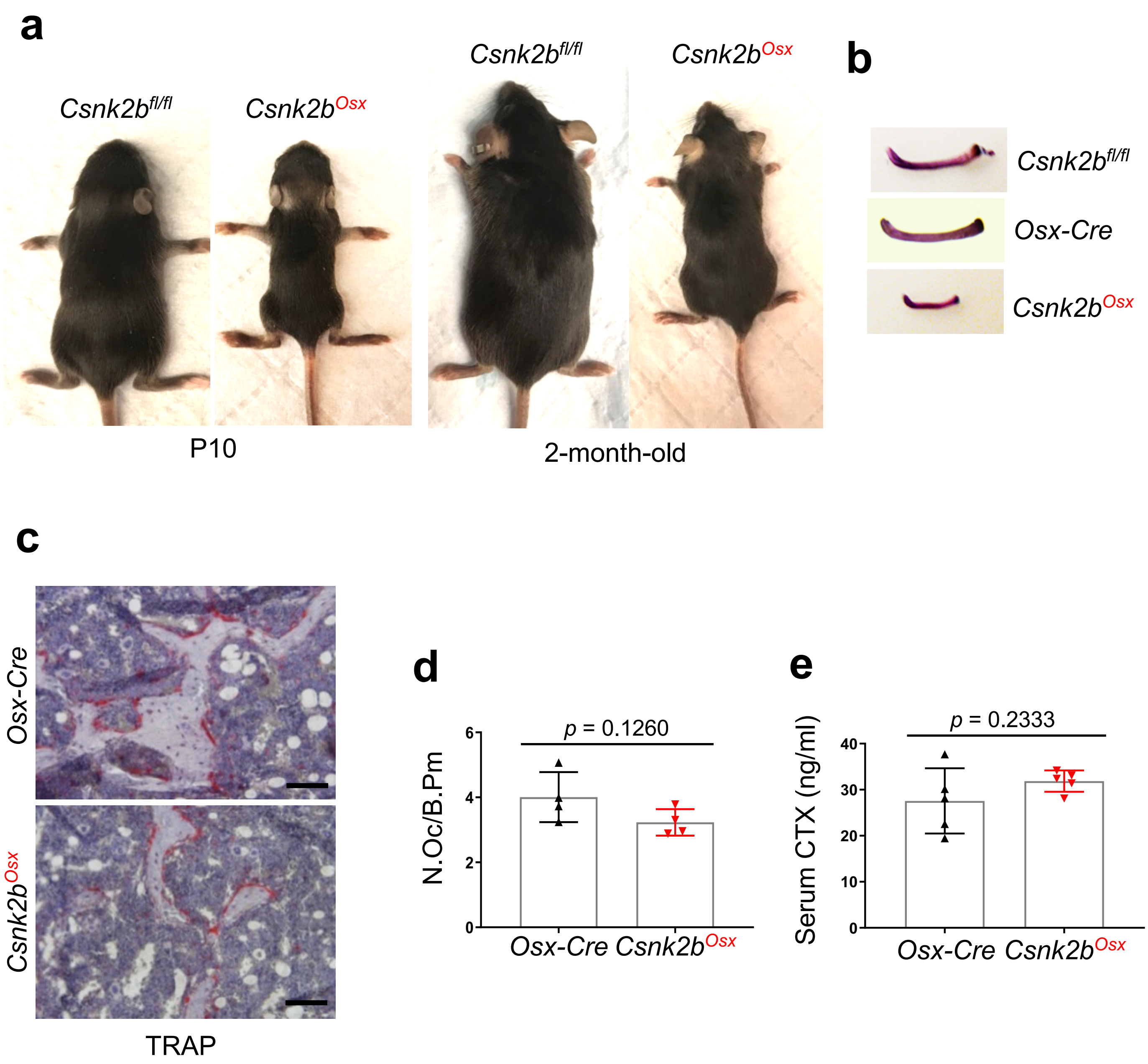

Supplementary Fig. 9: Characterization of *Csnk2b*^{Osx} mice.

(**a**) Representative photographs showing P10 (left) and 2-month-old (right) *Csnk2b*$^{fl/fl}$ and *Csnk2b*^{Osx} male mice. (**b**) Alizarin red/alcian blue staining of skeletal preparations of clavicles of 2-month-old *Csnk2b*$^{fl/fl}$, *Osx-cre* and *Csnk2b*^{Osx} male mice. (**c**) TRAP-stained longitudinal sections of femurs from 2-month-old *Osx-Cre* and *Csnk2b*^{Osx} female mice. Scale bar, 50 μm. (**d**) Histomorphometric analysis of femurs of 2-month-old *Osx-Cre* and *Csnk2b*^{Osx} female mice. N.Oc/B.Pm, number of osteoclasts per bone perimeter. (n = 4). (**e**) Serum CTX (cross-linked C-telopeptide of type 1 collagen) levels of 2-month-old *Osx-Cre* and *Csnk2b*^{Osx} male mice was assessed by ELISA. (n = 5). Data are representative of three independent experiments. A two-tailed unpaired Student's t-test for comparing two groups (**d, e;** error bars, SD of biological replicates).

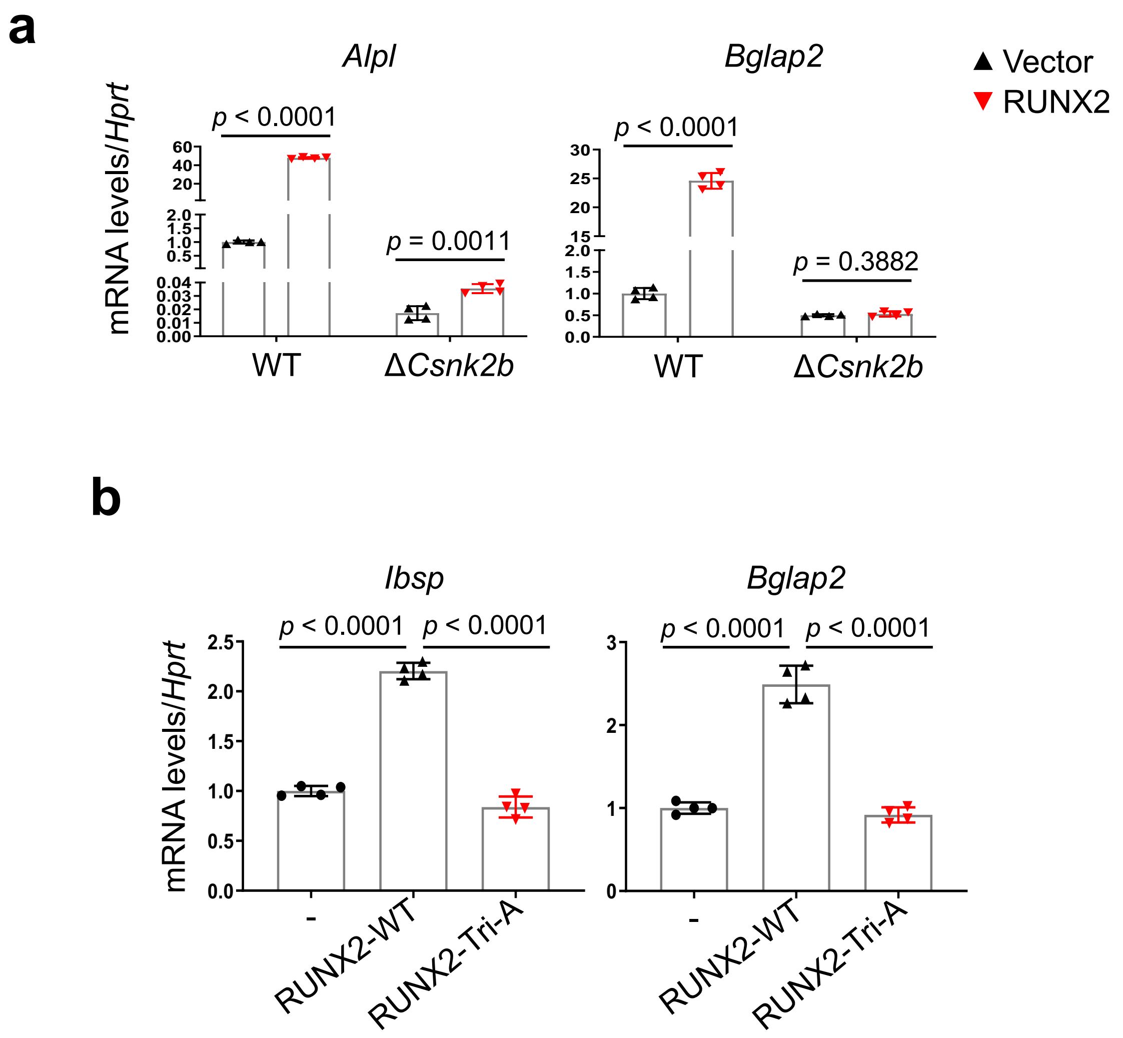

Supplementary Fig. 10: CK2 controls osteogenesis via RUNX2 phosphorylation.

(**a**) *Csnk2b*-sufficient (WT) or -deficient COBs (*Csnk2b* KO) were infected with lentiviruses expressing vector or RUNX2 and cultured under osteogenic conditions for 6 days. mRNA levels of osteogenic genes were measured by RT-PCR. (n = 4). (**b**) Wild type COBs were infected with lentiviruses expressing vector, RUNX2-WT or RUNX2-Tri-A mutant and cultured under osteogenic conditions for 6 days. mRNA levels of osteogenic genes were measured by RT-PCR. (n = 4). Data are representative of three independent experiments. A two-tailed unpaired Student's t-test for comparing two groups (**a**) and ordinary one-way ANOVA with Sidak's multiple comparisons test (**b;** error bars, SD of biological replicates). n represents biologically independent samples.

a

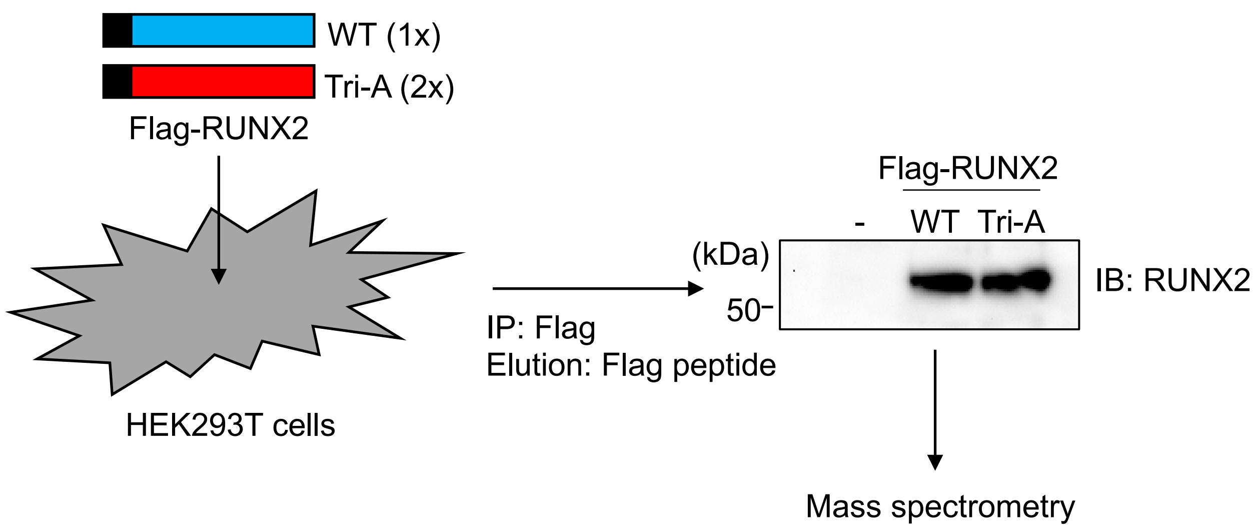

b

Gene	WT	Tri-A
HAUSP (USP7)	379.167	75
USP9X	8.421	2.211
USP10	21.111	8.056
USP24	13.333	1

Expr Intensity/RPKM/FPKM/Counts

c

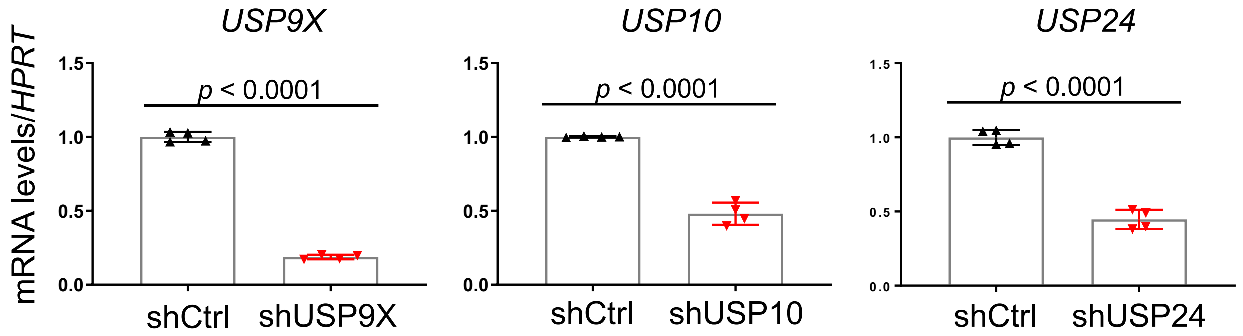

Supplementary Fig. 11: Identification of proteins that differentially bind to RUNX2 in a phosphorylation-dependent manner.

(**a**) A strategy to identify RUNX2-WT vs. RUNX2-Tri-A interacting proteins. (**b**) Intensity of counted peptides from mass spectrometry analysis. (**c**) Human BMSCs were infected with lentiviruses expressing control shRNA (shCtrl) or shRNAs targeting USP9X, USP10 or USP24 (shUSP9X, shUSP10, shUSP24) and cultured under osteogenic conditions for 6 days. mRNA levels were measured by RT-PCR. (n = 4 biologically independent samples.). Data in **c** are representative of three independent experiments. A two-tailed unpaired Student's t-test for comparing two groups (**c;** error bars, SD of biological replicates).

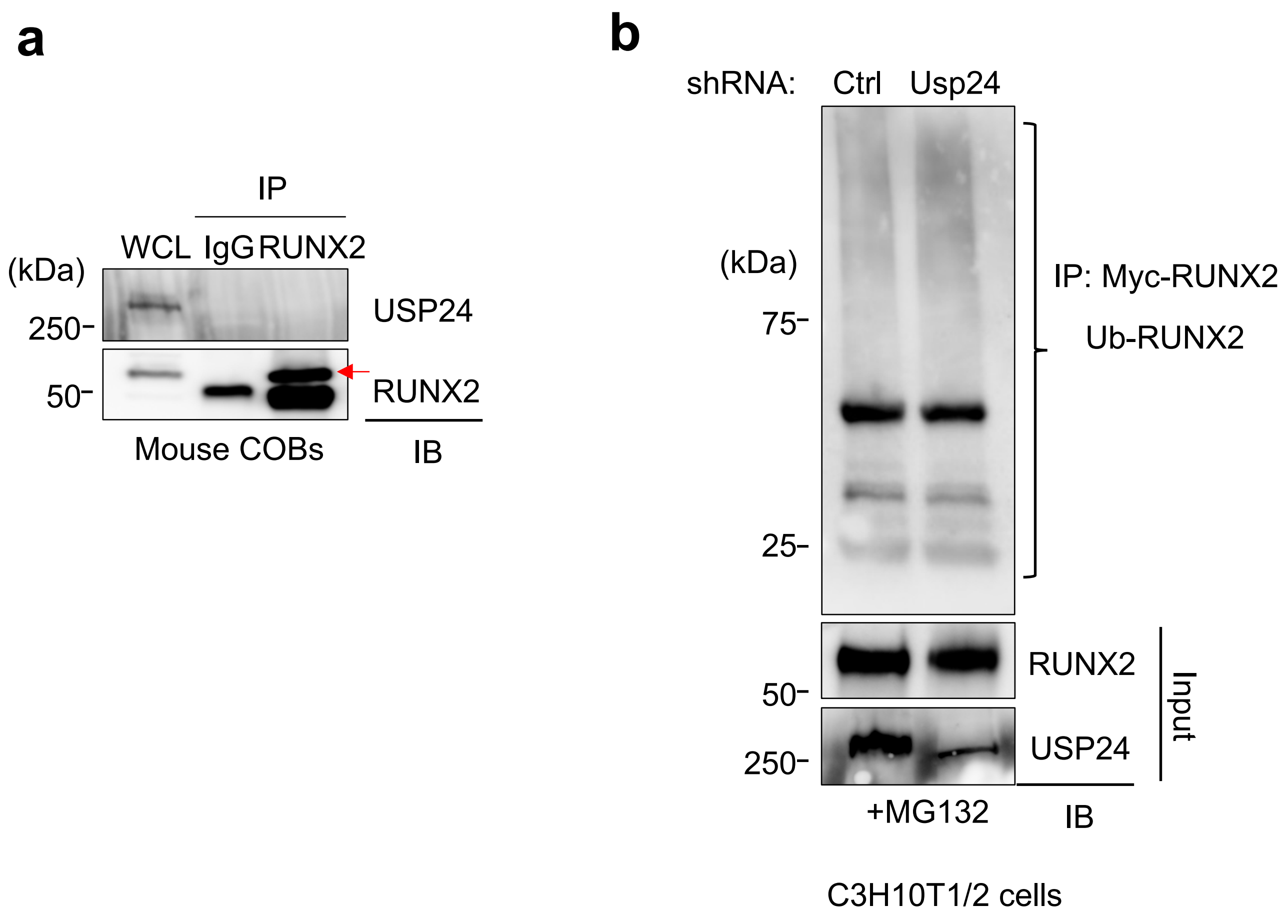

Supplementary Fig. 12: The DUB USP24 fails to bind and deubiquitinate RUNX2.

(**a**) Wild type COBs were lysed, immunoprecipitated with anti-IgG control or anti-RUNX2 antibody and protein G-conjugated agarose, and immunoblotted with the indicated antibodies. red arrow, RUNX2; WCL, whole cell lysate. (**b**) shCtrl or shUsp24-expressing C3H10T1/2 cells were transfected with Myc-RUNX2. 2 days after transfection, cells were treated with 10 μM MG132 for 6 h, lysed, immunoprecipitated with anti-Myc conjugated agarose, and immunoblotted with anti-ubiquitin antibody. Data are representative of two independent experiments.

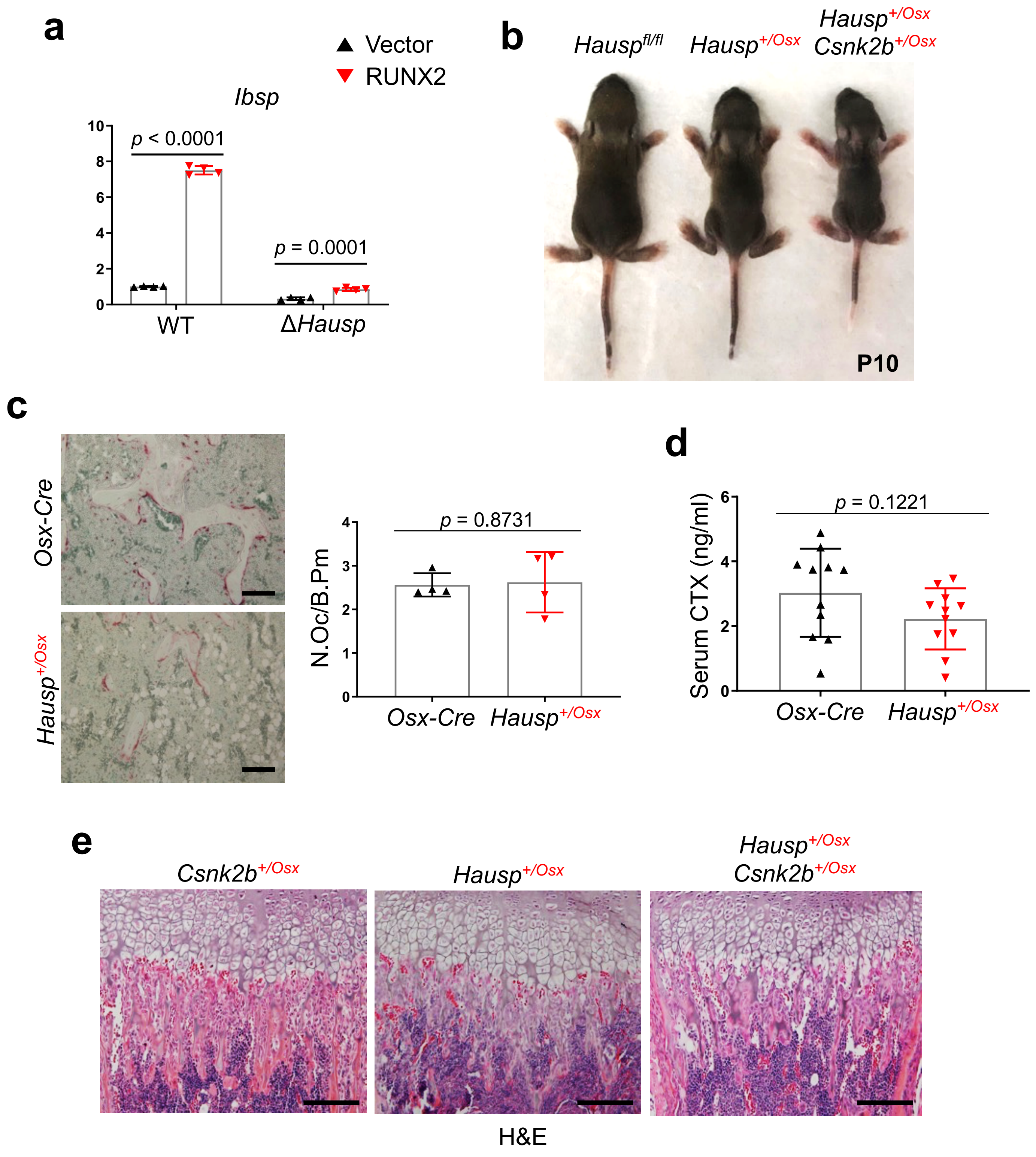

Supplementary Fig. 13: Characterization of skeletal phenotypes of *Hausp*$^{+/Osx}$ mice.

(**a**) *Hausp*-sufficient (WT) or –deficient COBs were infected with lentiviruses expressing vector or RUNX2, cultured under osteogenic conditions for 7 days, and mRNA levels of *Ibsp* were measured by RT-PCR. (n = 4). (**b**) Representative photographs of P10 *Hausp*$^{fl/fl}$, *Hausp*$^{+/Osx}$ and *Hausp*$^{+/Osx}$;*Csnk2b*$^{+/Osx}$ pups. (**c**) TRAP-stained longitudinal sections of femurs from 2-month-old *Osx-Cre* and *Hausp*$^{+/Osx}$ male mice (left) and quantification of osteoclast number (right). Scale bar, 50 μm. N.Oc/B.Pm, number of osteoclasts per bone perimeter. (n = 4). (**d**) Serum CTX (cross-linked C-telopeptide of type 1 collagen) levels of 2-month-old *Osx-Cre* and *Hausp*$^{+/Osx}$ male mice was assessed by ELISA. (n = 11). (**e**) H&E-stained longitudinal sections of P10 *Csnk2b*$^{+/Osx}$, *Hausp*$^{+/Osx}$, and *Hausp*$^{+/Osx}$;*Csnk2b*$^{+/Osx}$ femurs. Scale bar, 100 μm. Data are representative of three independent experiments. A two-tailed unpaired Student's t-test for comparing two groups (**a**, **c**, **d;** error bars, SD of biological replicates). n represents biologically independent samples.

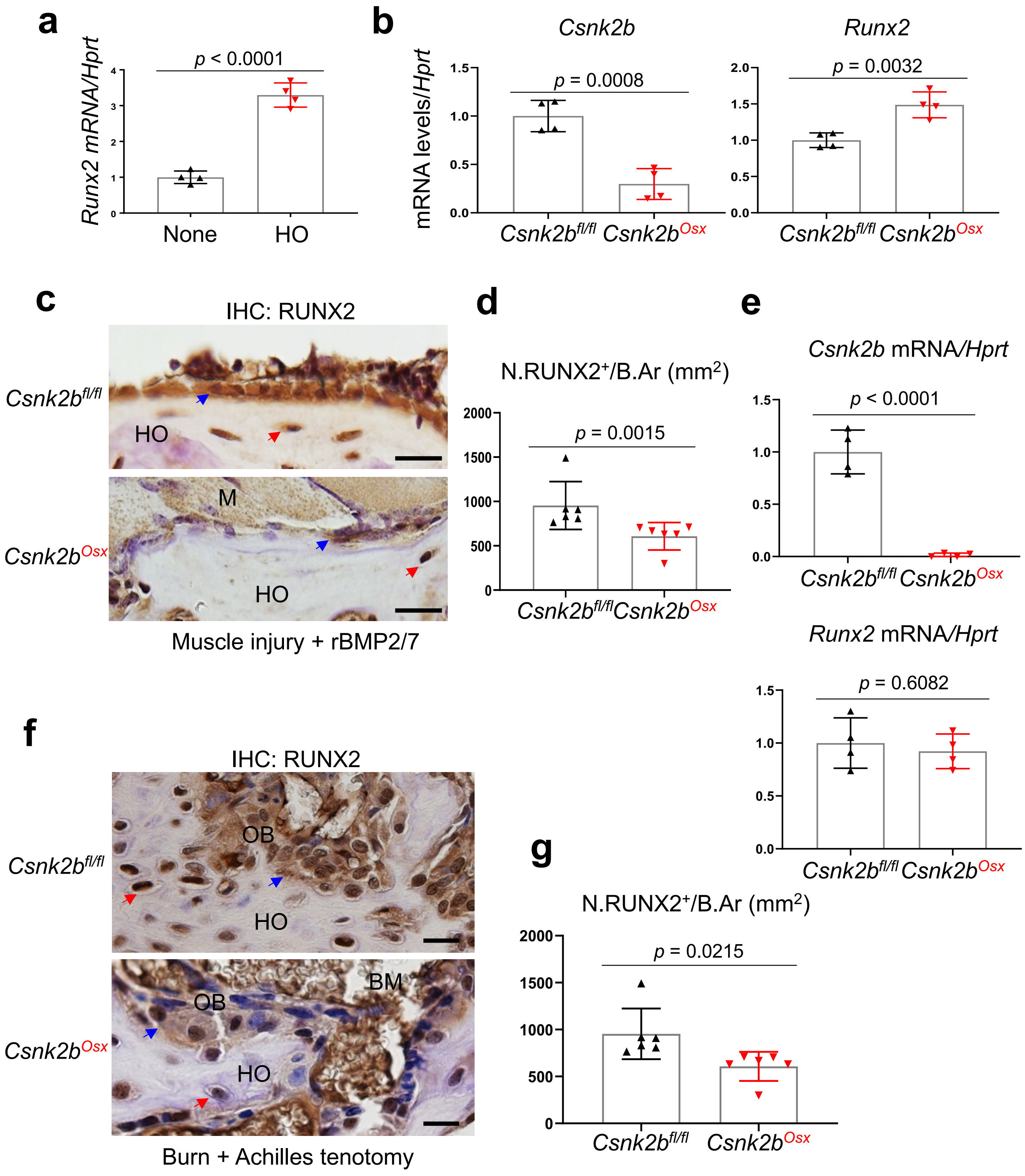

Supplementary Fig. 14: *Csnk2b*-deficiency decreases RUNX2 protein levels in mouse models of acquired HO.

(**a**) Achilles tenotomy was performed at 3-month-old male mice. 8 h after the injury, Achilles tendon was dissected and mRNA levels of *Runx2* were measured. None, non-tenotomized Achilles tendon; HO, tenotomized Achilles tendon. (n = 4). (**b-d**) Muscle injury and BMP2/7-matrigel injection were employed in 3-month-old $Csnk2b^{fl/fl}$ and $Csnk2b^{Osx}$ male mice and 3 weeks later, levels of *Csnk2b* and *Runx2* mRNA and RUNX2 protein in the HO areas were assessed by RT-PCR (**b**) and IHC (**c**, **d**), respectively. Representative images (**c**) and relative quantification of RUNX2-positive osteoblasts/osteocytes are displayed (**d**). Scale bar, 50 μm (**c**). (**b**, n = 4; **d**, n = 6). (**e-g**) Burn injury and Achilles tenotomy were employed in 3-month-old $Csnk2b^{fl/fl}$ and $Csnk2b^{Osx}$ male mice, and 8 weeks later, levels of *Csnk2b* and *Runx2* mRNA and RUNX2 protein in the HO areas were assessed by RT-PCR (**e**) and IHC (**f**, **g**), respectively. Scale bar, 50 μm (**f**). (**e**, n = 4; **g**, n = 6). HO, heterotopic bone; M, muscle; OB, osteoblast; BM, bone marrow; blue arrow, bone-lining osteoblasts in HO; red arrow, osteocytes in HO; N. $RUNX2^+$/B. AR, number of RUNX-positive cells/bone area. Data are representative of three (**a, b, e**) or two (**c, d, f, g**) independent experiments. A two-tailed unpaired Student's t-test for comparing two groups (**a**, **b**, **d**, **e**, **g;** error bars, SD of biological replicates).

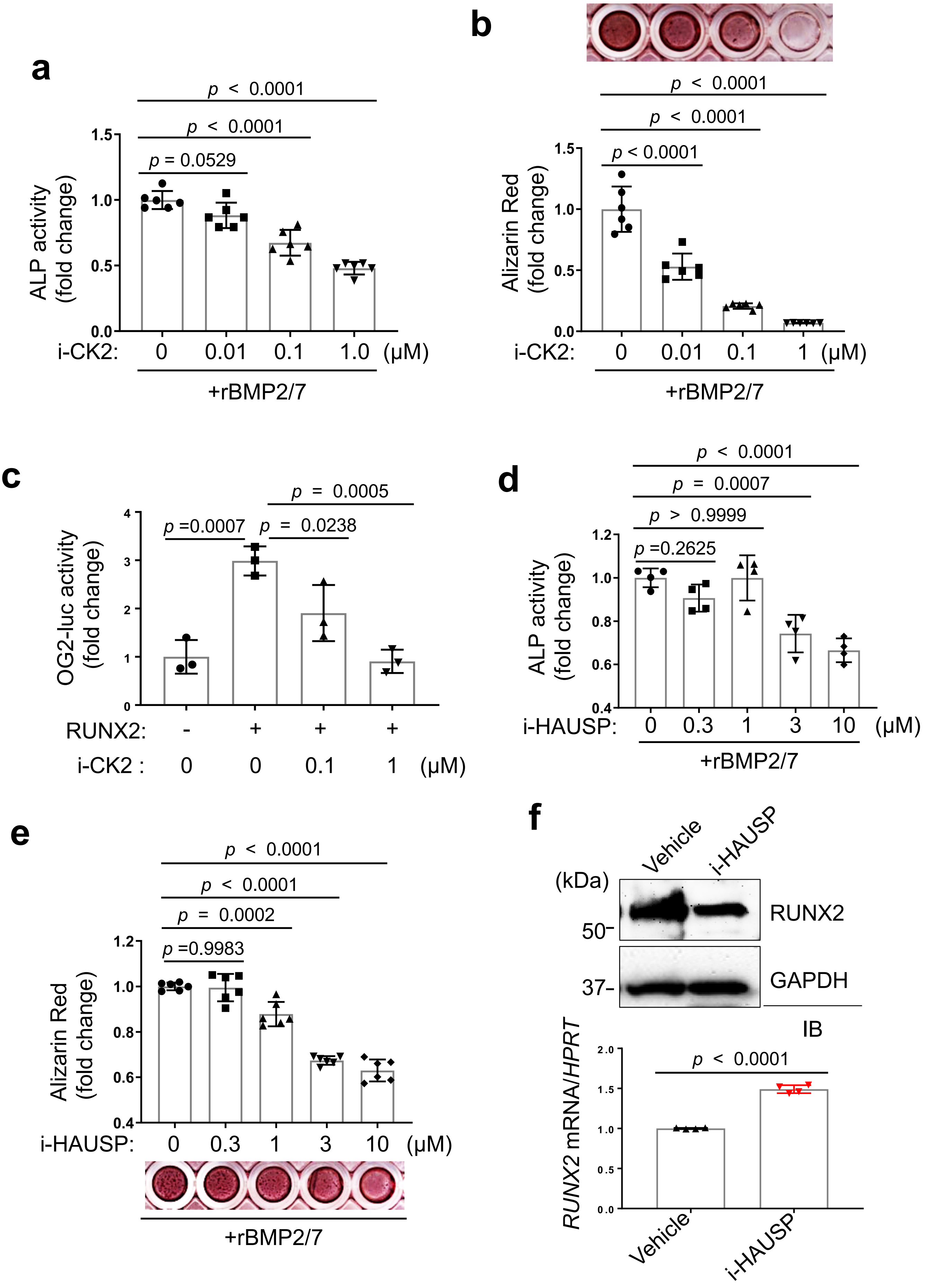

Supplementary Fig. 15: Effects of CK2/HAUSP inhibitors on osteogenesis.

(**a**, **b**, **d**, **e**) Wild type COBs were cultured under osteogenic conditions in the presence of rBMP2/7 (50 ng/ml) along with different concentrations of the inhibitor of CK2 or HAUSP. ALP activity was assessed 7 days after the culture (**a**, **d**). Mineralization was assessed by alizarin red staining at 18 days after the culture (**b**, **e**). (**a**, **b**, **e**, *n* = 6; **d**, *n* = 4). (**c**) C3H10T1/2 cells were transfected with OG2-luc and *Renilla* in the presence or absence of RUNX2 and treated with different concentrations of CK2 inhibitor (i-CK2). OG2-luc activity was measured and normalized to *Renilla*. (*n* = 3). (**f**) Protein (upper) or mRNA levels (lower) of RUNX2 in human BMSCs 24 hours after treatment with DMSO (vehicle) or 10 uM of HAUSP inhibitor (i-HAUSP). (**f**, *n* = 4). Data are representative of three independent experiments. Ordinary one-way ANOVA with Dunnett's multiple comparisons test (**a**-**e**) and a two-tailed unpaired Student's t-test for comparing two groups (**f;** error bars, SD of biological replicates).

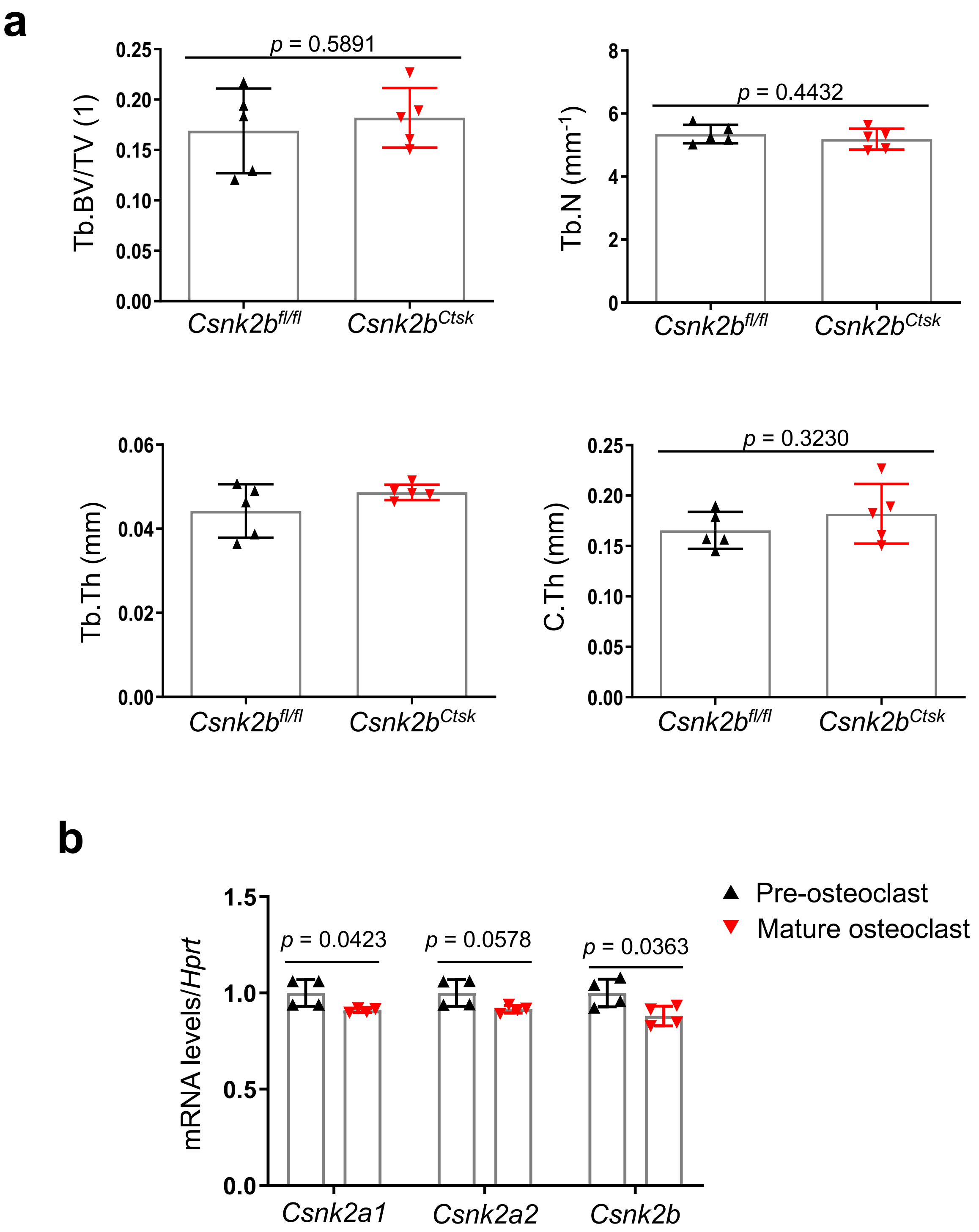

Supplementary Fig. 16: The CK2 pathway is dispensable for osteoclast development *in vivo*.

(**a**) MicroCT analysis of femurs of 2-month-old $Csnk2b^{fl/fl}$ and $Csnk2b^{Ctsk}$ male mice. Quantitative parameters showing trabecular bone volume/total volume (Tb.BV/TV), trabecular thickness (Tb.Th), trabecular number per cubic millimeter (Tb.N), and cortical thickness (C.Th). (n = 5). (**b**) Mouse bone marrow-derived monocytes (BMM) isolated from 2-month-old wild type mice were treated with M-CSF (20 ng/ml) alone for pre-osteoclast culture or with M-CSF (20 ng/ml) and RANKL (10 ng/ml) for mature osteoclast culture. 6 days later, mRNA levels of CK2 subunits were measured by RT-PCR. (n = 4). Data in **b** are representative of three independent experiments. A two-tailed unpaired Student's t-test for comparing two groups (**a**, **b;** error bars, SD of biological replicates).

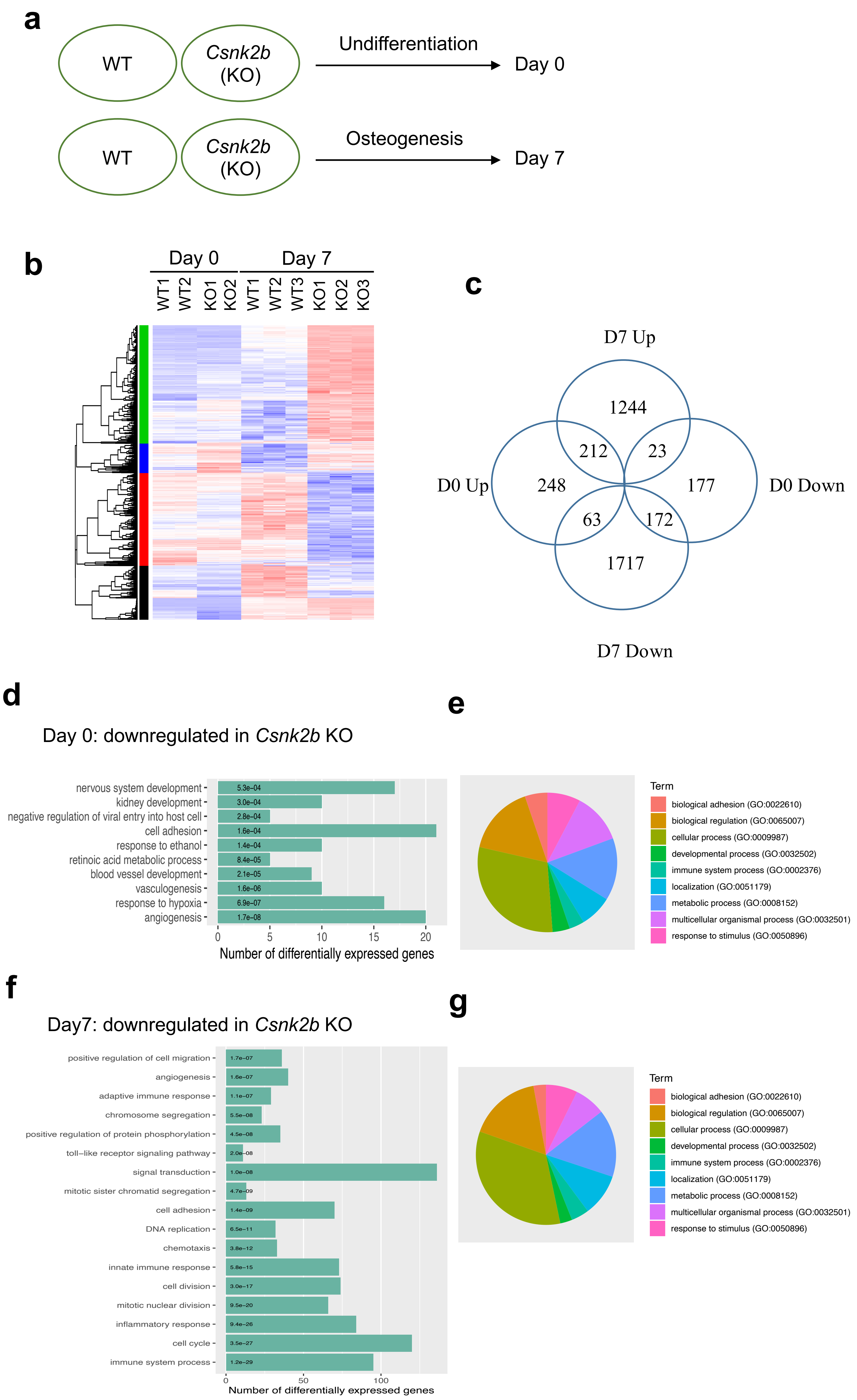

a
WT
Csnk2b (KO)
Undifferentiation
Day 0
WT
Csnk2b (KO)
Osteogenesis
Day 7
b
Day 0
Day 7
WT1 WT2 KO1 KO2 WT1 WT2 WT3 KO1 KO2 KO3
c
D7 Up
1244
212
23
D0 Up
248
177
D0 Down
63
172
1717
D7 Down
d
Day 0: downregulated in Csnk2b KO
nervous system development 5.3e−04
kidney development 3.0e−04
negative regulation of viral entry into host cell 2.8e−04
cell adhesion 1.6e−04
response to ethanol 1.4e−04
retinoic acid metabolic process 8.4e−05
blood vessel development 2.1e−05
vasculogenesis 1.6e−06
response to hypoxia 6.9e−07
angiogenesis 1.7e−08
0 5 10 15 20
Number of differentially expressed genes
e
Term
biological adhesion (GO:0022610)
biological regulation (GO:0065007)
cellular process (GO:0009987)
developmental process (GO:0032502)
immune system process (GO:0002376)
localization (GO:0051179)
metabolic process (GO:0008152)
multicellular organismal process (GO:0032501)
response to stimulus (GO:0050896)
f
Day7: downregulated in Csnk2b KO
positive regulation of cell migration 1.7e−07
angiogenesis 1.6e−07
adaptive immune response 1.1e−07
chromosome segregation 5.5e−08
positive regulation of protein phosphorylation 4.5e−08
toll−like receptor signaling pathway 2.0e−08
signal transduction 1.0e−08
mitotic sister chromatid segregation 4.7e−09
cell adhesion 1.4e−09
DNA replication 6.5e−11
chemotaxis 3.8e−12
innate immune response 5.8e−15
cell division 3.0e−17
mitotic nuclear division 9.5e−20
inflammatory response 9.4e−26
cell cycle 3.5e−27
immune system process 1.2e−29
0 50 100
Number of differentially expressed genes
g
Term
biological adhesion (GO:0022610)
biological regulation (GO:0065007)
cellular process (GO:0009987)
developmental process (GO:0032502)
immune system process (GO:0002376)
localization (GO:0051179)
metabolic process (GO:0008152)
multicellular organismal process (GO:0032501)
response to stimulus (GO:0050896)

Supplementary Fig. 17: Transcriptome analysis of WT and *Csnk2b*-deficient COBs.

(**a**) Strategy for sample preparation for RNA sequencing and analysis. COBs isolated from P5 *Csnk2b*$^{fl/fl}$ pups were infected with lentiviruses expressing vector (WT) or Cre recombinase (*Csnk2b* KO) and cultured under undifferentiated (day 0) or osteogenic conditions for 7 days (day 7). (**b**) Heat map of total gene expression in WT and *Csnk2b* KO COBs. 3850 total genes were clustered into four groups, and with groups consisting of 1544 (green, group 1), 385 (blue, group 2), 1214 (red, group 3), and 707 genes (black, group 4). (**c**) Venn diagram showing numbers of up-regulated or down-regulated genes in *Csnk2b* KO COBs relative to WT COBs. (**d**, **f**) Gene ontology (GO) enrichment analysis showing differentially expressed genes (DEGs) in WT and *Csnk2b* KO COBs at day 0 (**d**) or day 7 (**f**). Numbers in the bar represent the p value of the enrichment analysis (**e**, **g**) CateGOrizer summarizes biological process functions from GO based on DEGs between WT and *Csnk2b* KO COBs at day 0 (**e**) or day 7 (**g**). The fraction of the pie chart presents the proportion of genes involving with given function.

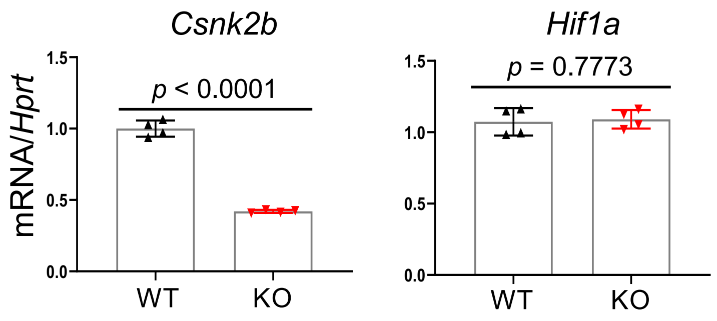

Pro-angiogenic factor

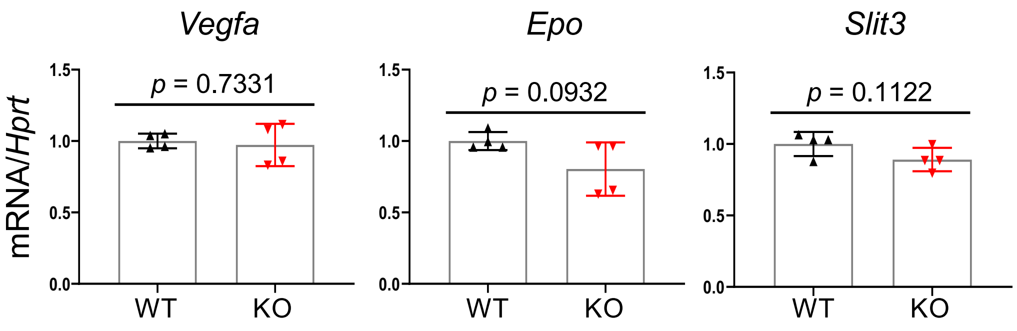

Anti-angiogenic factor

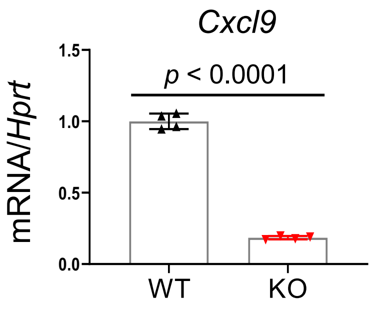

Supplementary Fig. 18: Expression of angiogenic factors in WT and *Csnk2b*-deficient osteoblasts.

WT and *Csnk2b* KO COBs were cultured under osteogenic conditions for 7 days and mRNA levels of *Csnk2b* and osteoblast-derived angiogenic factors were examined by RT-PCR. (n = 4 biologically independent samples.). Data are representative of three independent experiments. A two-tailed unpaired Student's t-test for comparing two groups (error bars, SD of biological replicates).

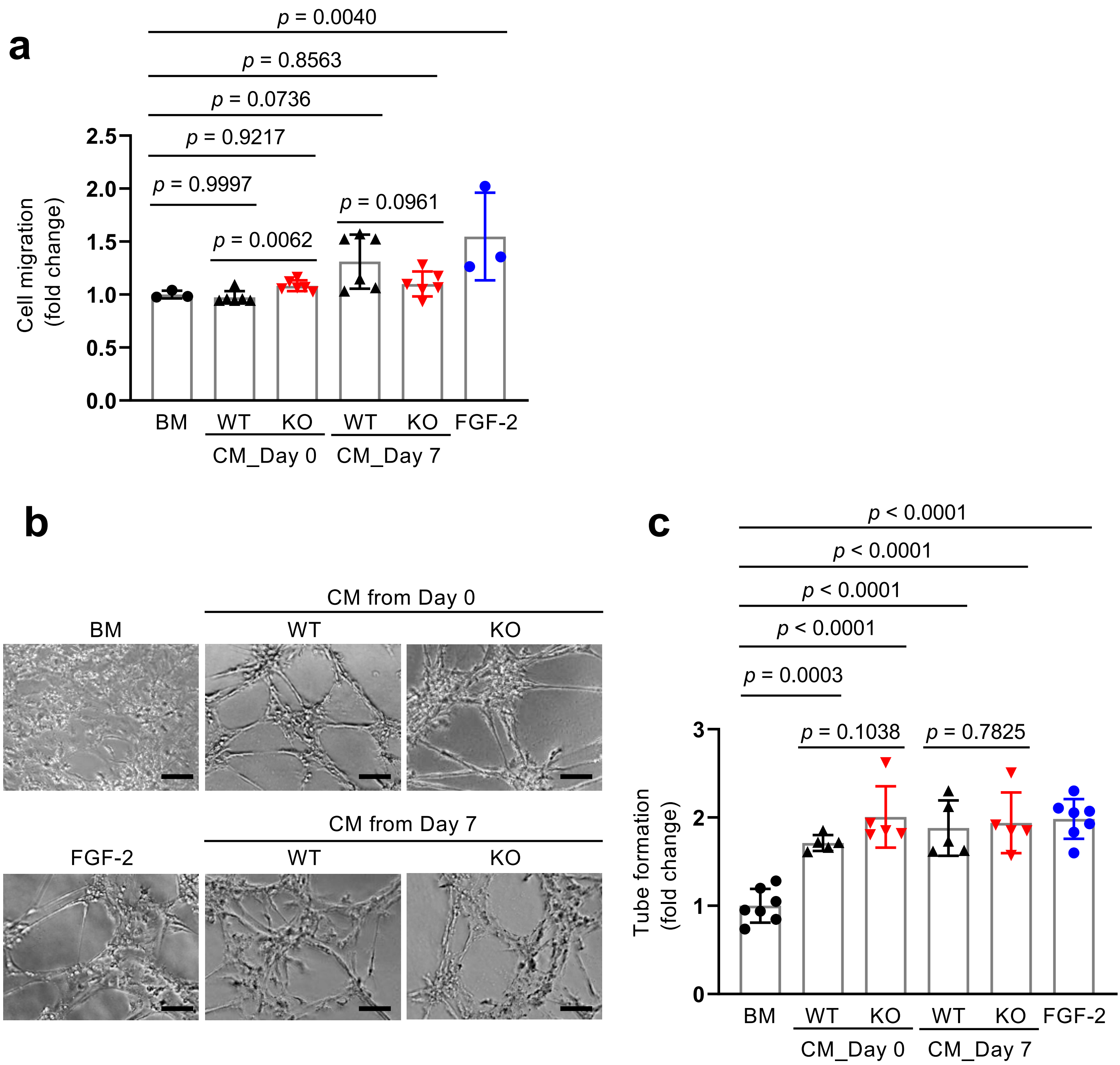

Supplementary Fig. 19: *Csnk2b*-deficiency in osteoblasts does not affect angiogenesis.

(**a**) A transwell migration assay was performed using mouse bone marrow-derived endothelial progenitor outgrowth cells (EPOCs). Conditioned medium (CM) were harvested from WT and *Csnk2b* KO COBs cultured under undifferentiated (Day 0) or osteogenic conditions (Day 7). EPOCs on the transwell plate were incubated with either basal medium (BM), conditioned medium (CM), or FGF-2 (20 ng/ml) for 6 hours and numbers of migrated cells were quantified (n = 3 (BM, FGF-2), or 6 (CM)). (**b**, **c**) A matrigel tube formation assay was performed using mouse EPOCs. EPOCs were seeded in a matrigel and incubated with BM, CM or FGF-2 (20 ng/ml) for 24 hours. Representative images (**b**) and relative quantification (**c**) of EPOCs-induced total tube length are displayed. Scale bar, 100 μm (**b**). (n = 5 (CM) or 7 (BM, FGF-2)). Data are representative of three independent experiments. A two-tailed unpaired Student’s t-test for comparing two groups (WT CM vs. KO CM) and ordinary one-way ANOVA with Dunnett's multiple comparisons test (BM vs.CM or FGF-2). Error bars, SD of biological replicates.

Fig. 4c

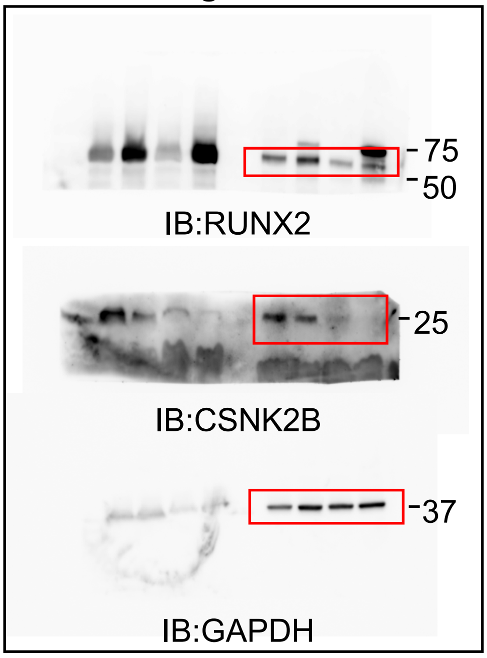

Fig. 4d

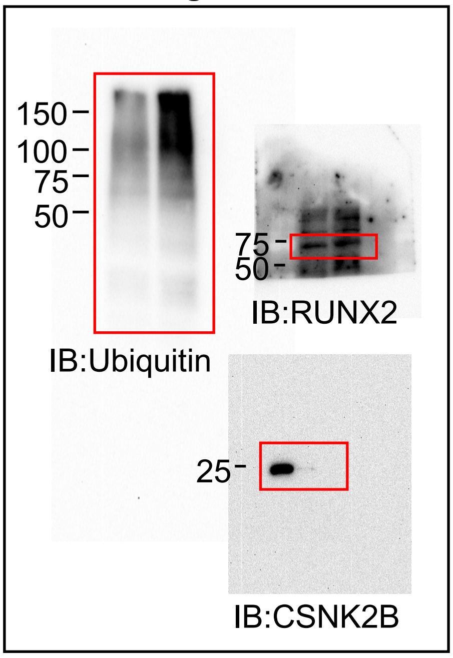

Fig. 4e (top)

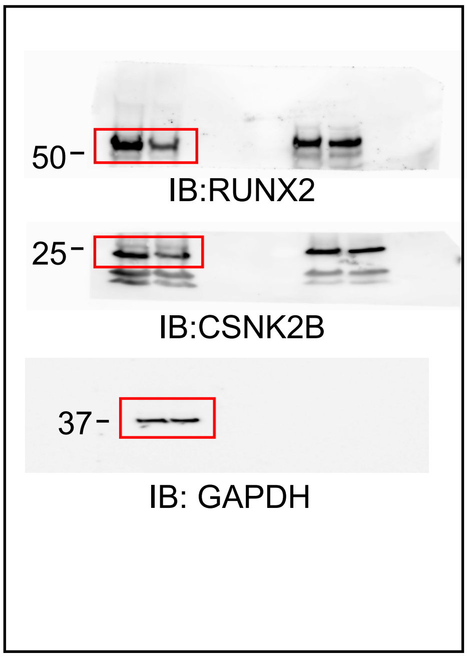

Fig. 4e (bottom)

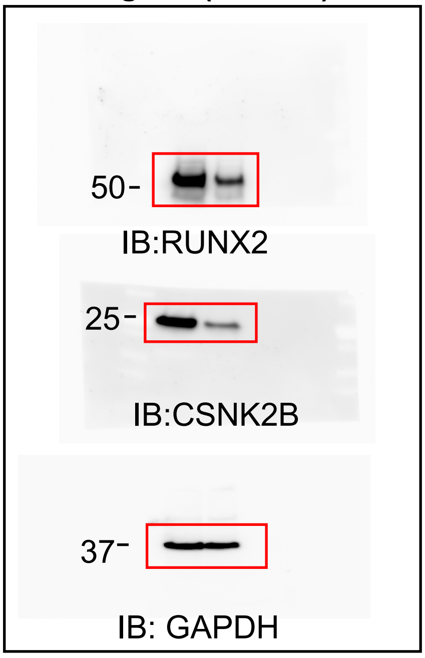

Fig. 4g

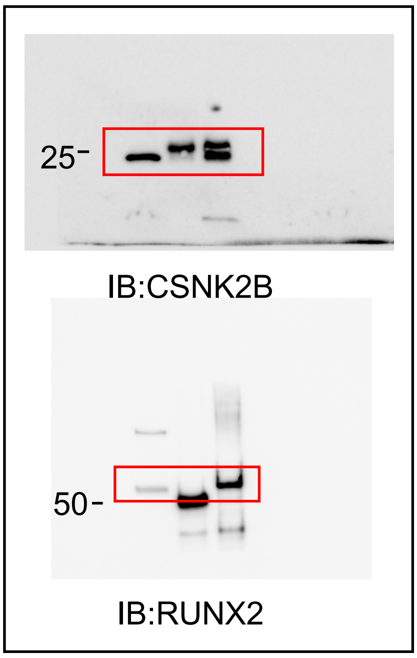

Fig. 4h

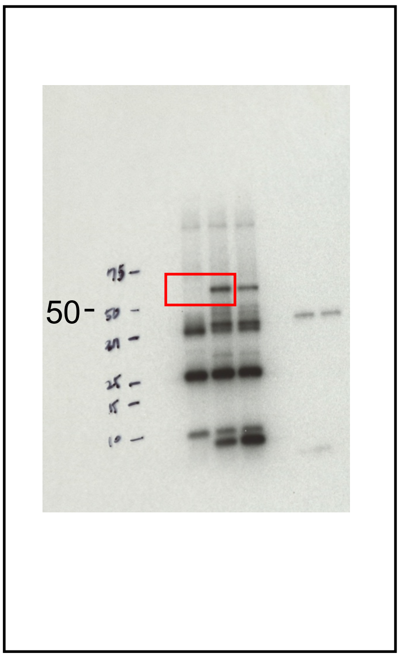

Fig. 4l

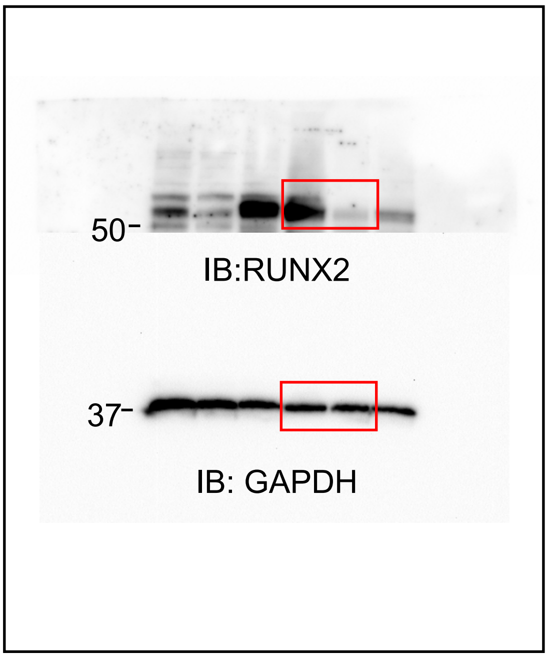

Fig. 4n

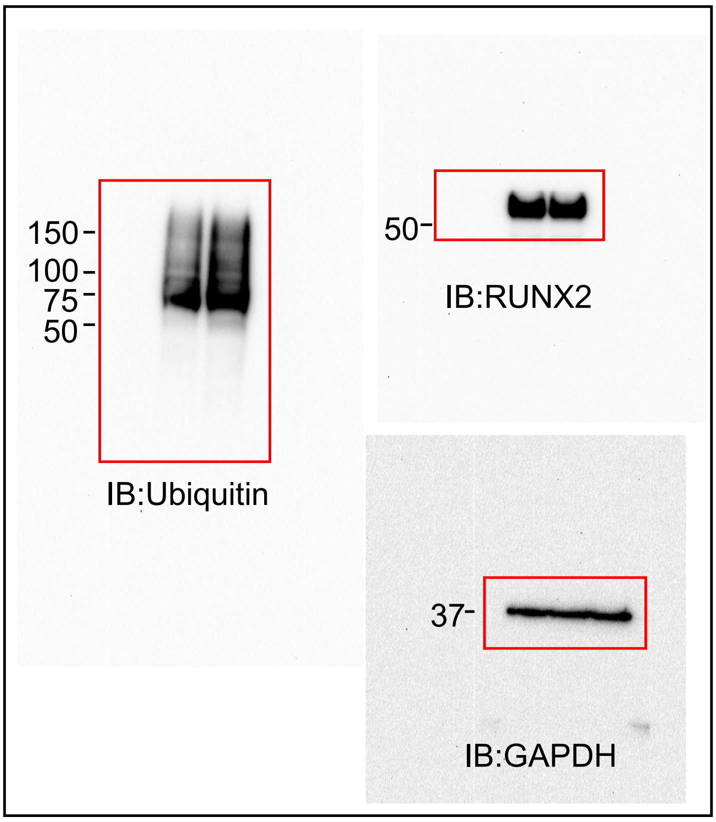

Supplementary Fig. 20

Fig. 5e

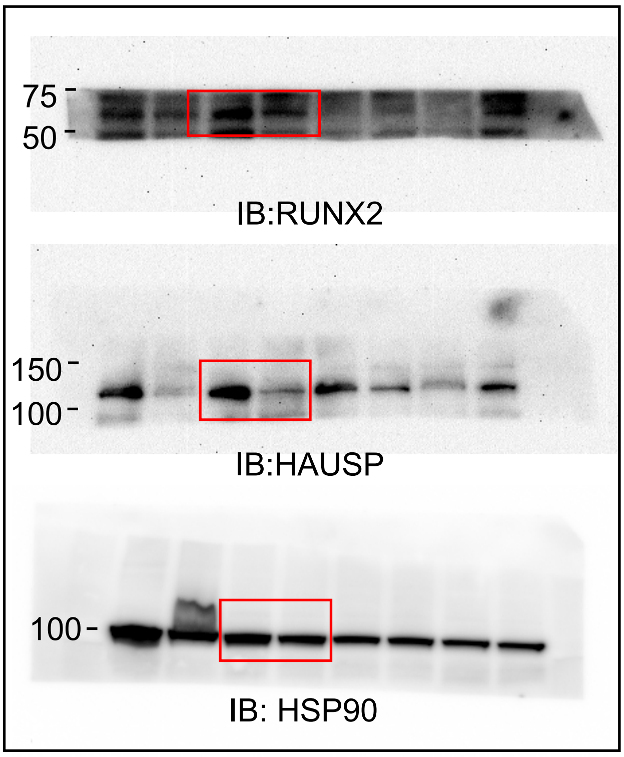

Fig. 5g

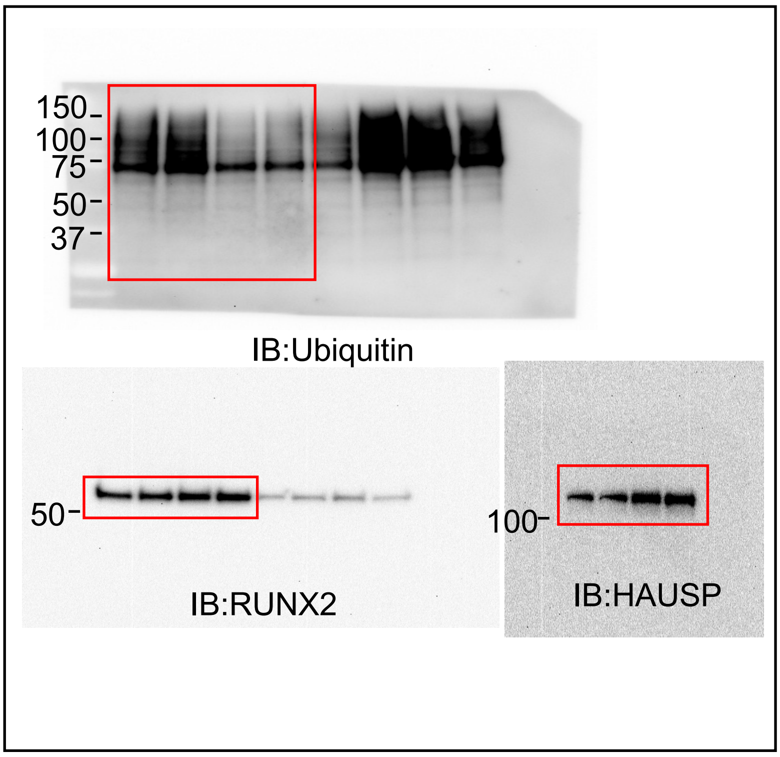

Fig. 5h

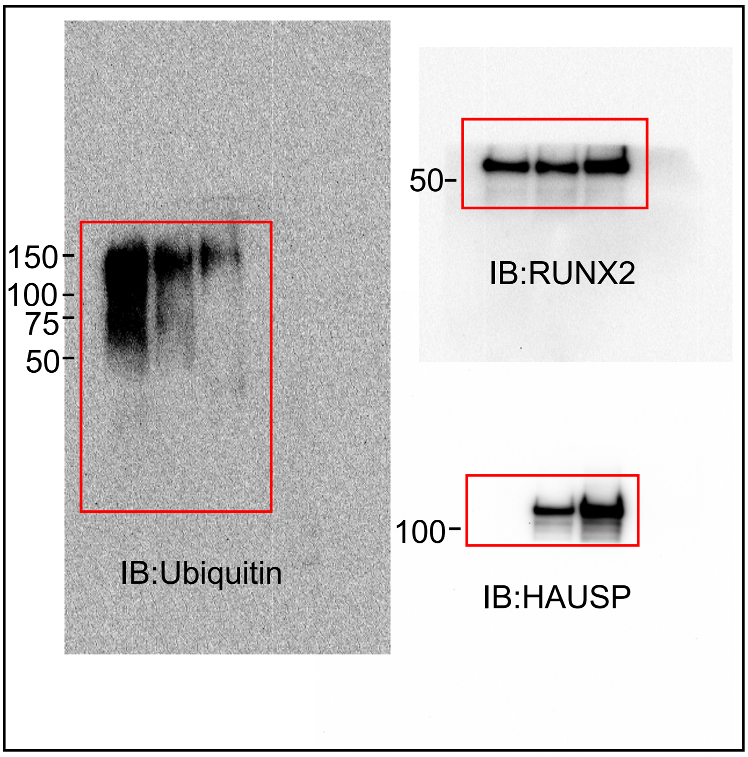

Fig. 5i

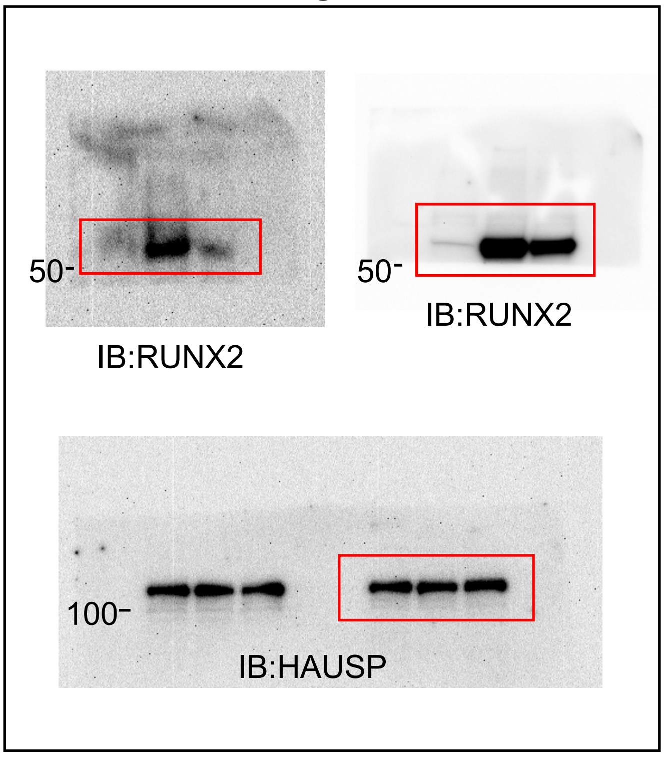

Fig. 5j

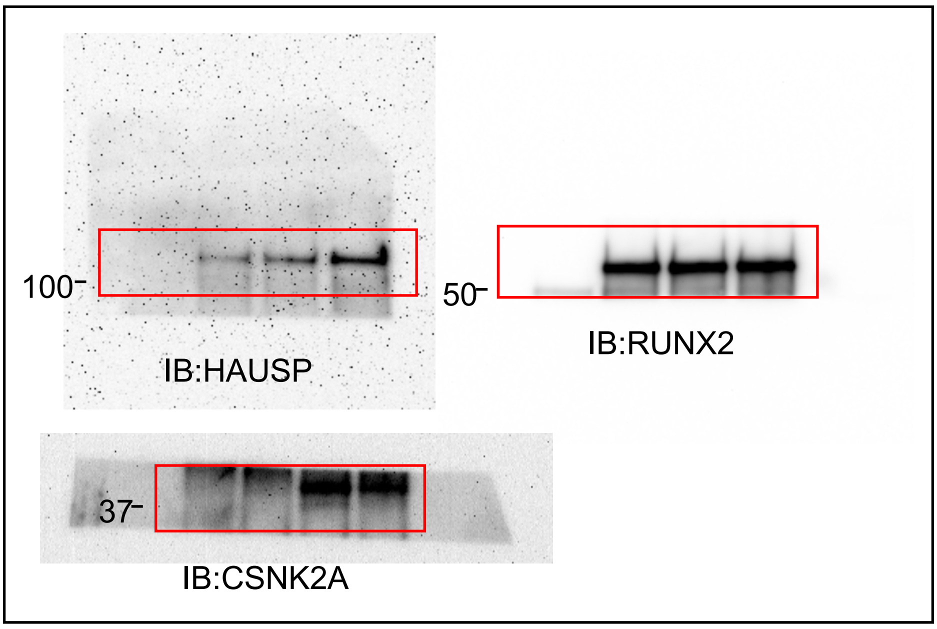

Supplementary Fig. 21

Fig. 5k

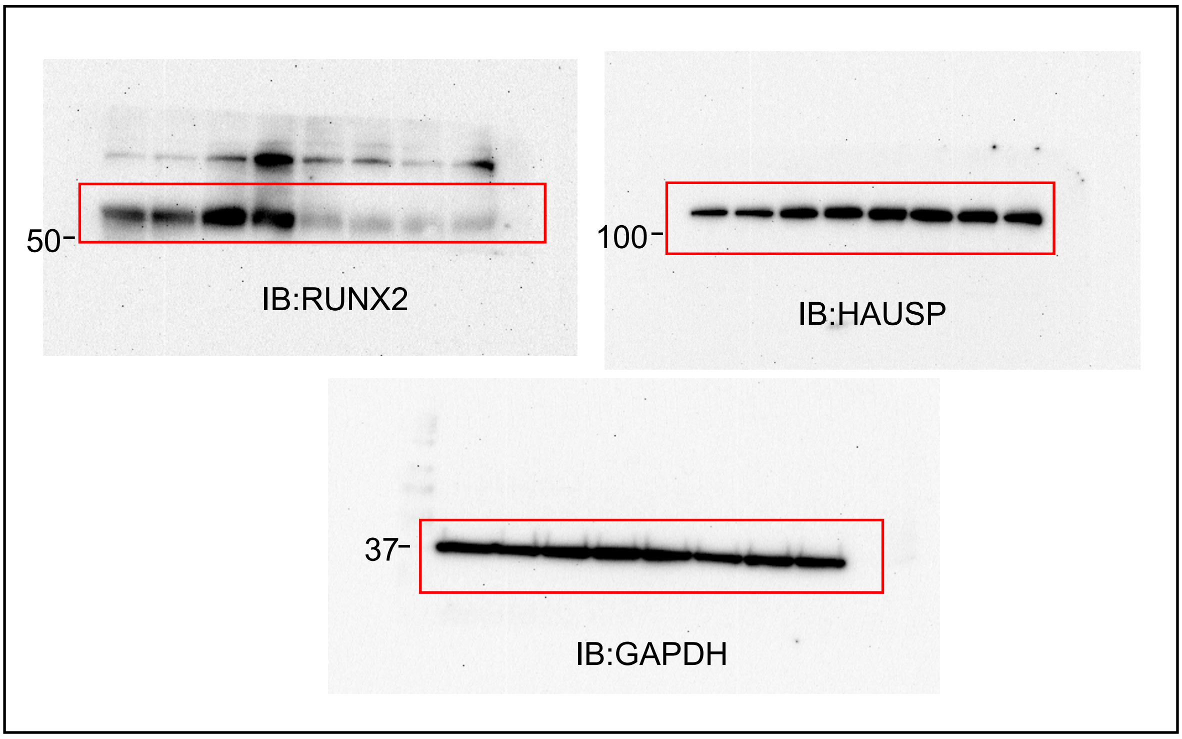

Fig. 6d

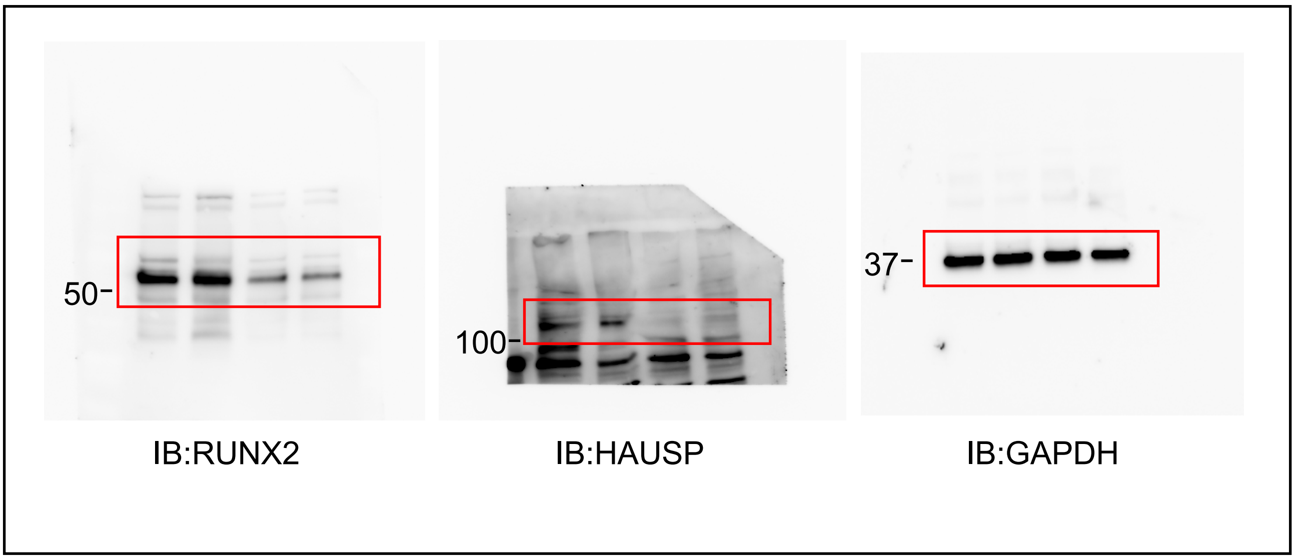

Supplementary Fig. 22

Supplementary Fig. 2c

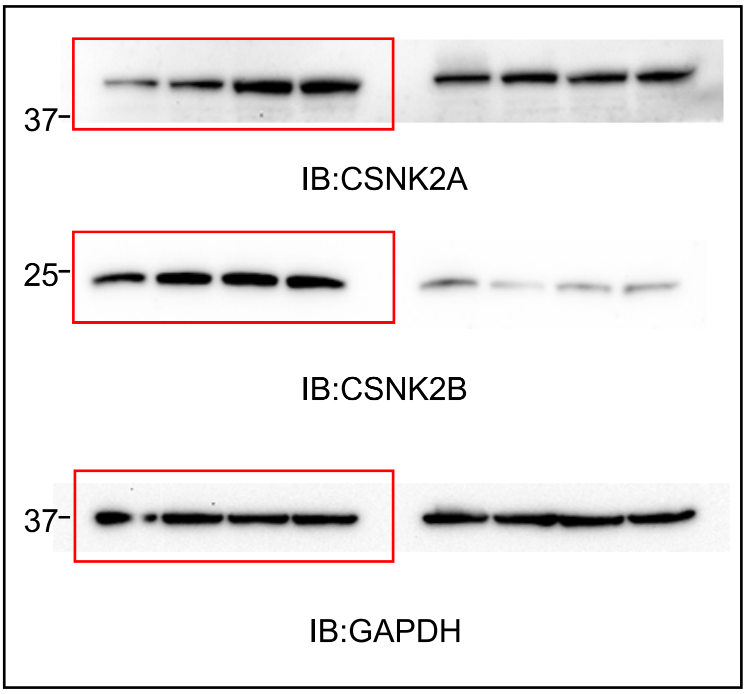

Supplementary Fig. 11a

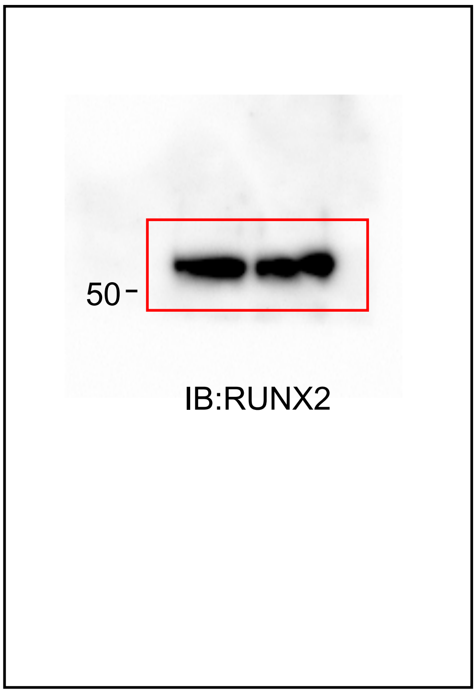

Supplementary Fig. 12a

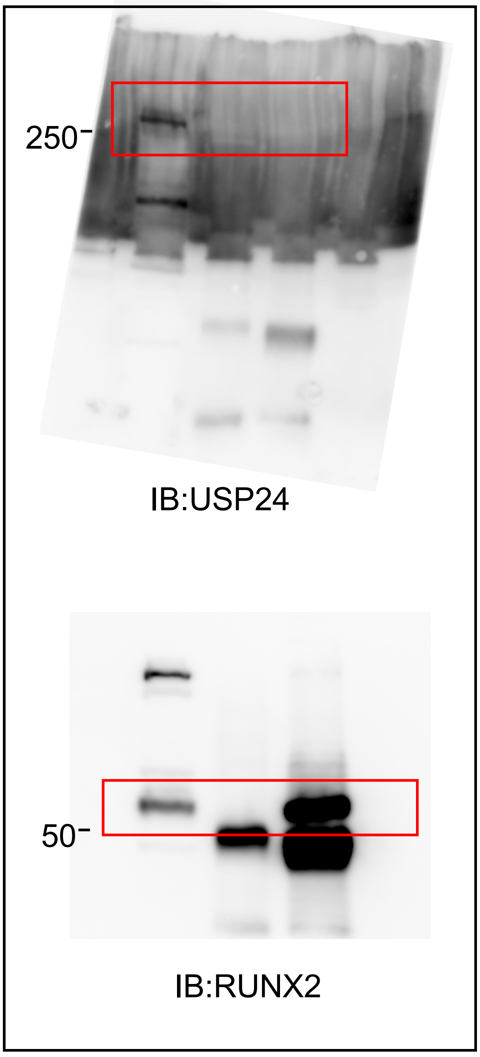

Supplementary Fig. 12b

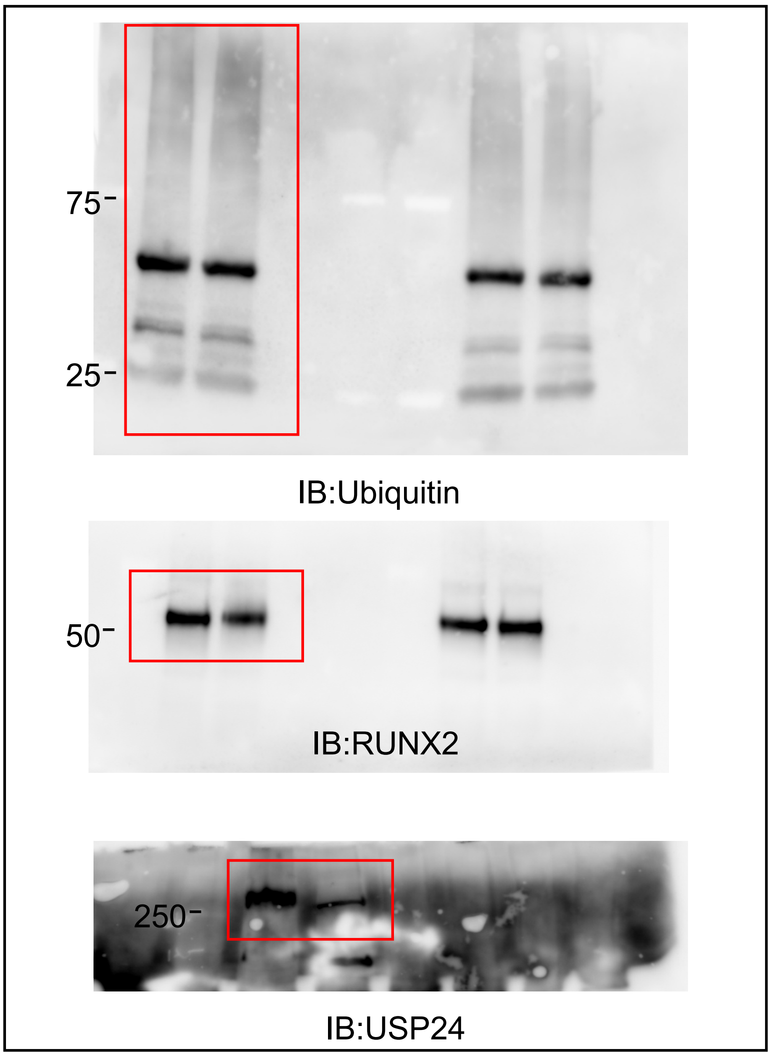

Supplementary Fig. 12b

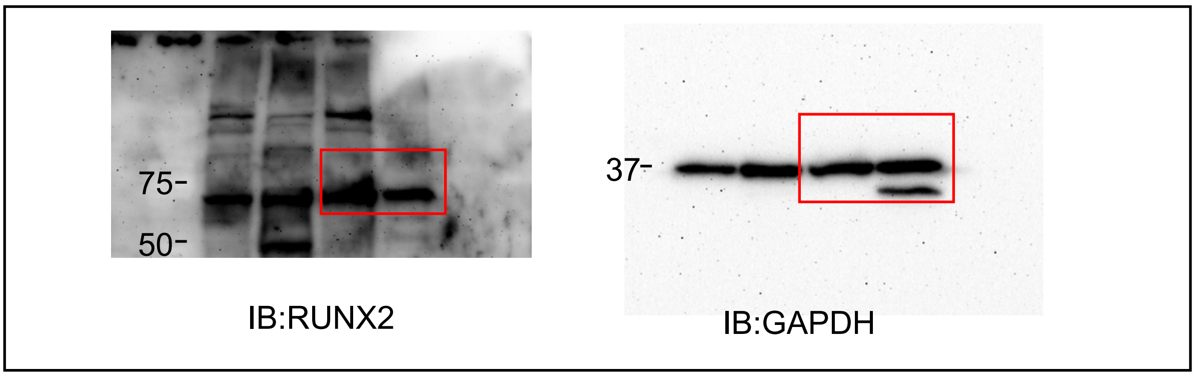

Supplementary Fig. 23

Supplementary Table 1

Primer sequences

Gene	Forward	Reverse
Human *CSNK2A1*	CGACTAATAGACTGGGGTTTGG	TCATACTTGCCAGCATACAACC
Human *CSNK2A2*	AATGTTCGTGTAGCCTCAAGGT	CTGGTCATAGTTGTCCTGTCCA
Human *CSNK2B*	GCCTGATGAAGAACTGGAAGAC	TAACCAAAGTCTCCTTGCTGGT
Human *ALPL*	TAACATCAGGGACATTGACG	TGCTTGTATCTCGGTTTGAA
Human *BGLAP2*	AGAGTCCAGCAAAGGTGCAG	TCAGCCAACTCGTCACAGTC
Human *RUNX2*	TTACTTACACCCCGCCAGTC	CACTCTGGCTTTGGGAAGAG
Human *SP7*	TTACAAGCACTAATGGGCTCCT	GTAGACACTGGGCAGACAGTCA
Human *IBSP*	CAACAGCACAGAGGCAGAAA	TTGTGGTGGGGTTGTAGGTT
Human *COL1A1*	GTACATCAGCAAGAACCCCAAG	TTGCAGTGGTAGGTGATGTTCT
Human *SOX9*	ATCAAGACGGAGCAGCTGAG	TGGTGGTCGGTGTAGTCGTA
Human *ACAN*	GAATCAACTGCTGCAGACCA	ATGCTGCTCAGGTGTGACTG
Human *COL2A1*	GTCTACCCCAATCCAGCAAA	GTTGGGAGCCAGATTGTCAT
Human *PPARG*	ATGGAGTCCACGAGATCATT	CGCAGGCTCTTTAGAAACTC
Human *CEBPA*	CAAGAAGTCGGTGGACAAGAAC	ATTGTCACTGGTCAGCTCCAG
Human *FABP4*	AACCTTAGATGGGGGTGTCCTG	TCGTGGAAGTGACGCCTTTC
Human *HAUSP*	TGTGATCAATGGGAATGTGG	CGCTCCACAGTGAACTGAAA
Human *USP9X*	AGGATGTGGGTCGTTACAGC	CTTCCAATGCTTCACAGCAA
Human *USP10*	GGAAAAACTCCCTCCTGTCC	AACCCCTGGAGAAAGCAGTT
Human *USP24*	GATGGAGTTCAGCACAAGCA	GAGCAATGCTAATGGGTGGT
Human *HPRT*	GCTATAAATTCTTTGCTGACCTGCTG	AATTACTTTTATGTCCCCTGTTGACTGG
Mouse *Csnk2a1*	TATGTGGAGCTTGGGTTGTATG	CAAGATATCGTTGAAACGTGGA
Mouse *Csnk2a2*	CAACAATGAGAGGGTGGTTGTA	TGACACAGGGTCCTTTACAGTG
Mouse *Csnk2b*	TCTTCTGTGAGGTGGATGAAGA	CTCTTCATCAGGTTCCAGGTCT
Mouse *Alpl*	CACAATATCAAGGATATCGACGTGA	ACATCAGTTCTGTTCTTCGGGTACA
Mouse *Runx2*	TACAAACCATACCCAGTCCCTGTTT	AGTGCTCTAACCACAGTCCATGCA
Mouse *Ibsp*	CAGGGAGGCAGTGACTCTTC	AGTGTGGAAAGTGTGGCGTT
Mouse *Sp7*	ATGGCGTCCTCTCTGCTTGA	GAAGGGTGGGTAGTCATTTG
Mouse *Bglap2*	GCAGCACAGGTCCTAAATAG	GGGCAATAAGGTAGTGAACAG
Mouse *Col1a1*	ACTGTCCCAACCCCCAAAG	ACGTATTCTTCCGGGCAGAA
Mouse *Hausp*	ACGATTGCAAGAAGAGAAAAGG	TCCTCGTCGTACATGTCATTTC
Mouse *Vegfa*	AAACTTTTCGTCCAACTTCTGG	TTCTCTTCCTCCCCCTCTCTTCT
Mouse *Hif1a*	CCTTCATCGGAAACTCCAAA	TGGGGCATGGTAAAAGAAAG
Mouse *Epo*	CCACCCTGCTGCTTTTACTC	GCCTCCTTGGCCTCTAAGAT
Mouse *Park7*	TATCTGAGTCGCTGGTGAA	CCTTGCATCCAAAACCTACTTC
Mouse *Slit3*	TCCAGTGTTCCTGAAGGCTCCT	TGGCAATGCCAGGCTCCTTGTA
Mouse *Cxcl9*	ACGGAGATCAAACCTGCCTA	TTTCCCCCTCTTTTGCTTTT
Mouse *Hprt*	CTGGTGAAAAGGACCTCTCGAAG	CCAGTTTCACTAATGACACAAACG

Supplementary Table 2

Group1 : Green, 1544 genes

Term	Count	%	PValue	Genes
immune system process	65	0.0733	5.93E-27	APOBEC3, ZC3HAV1, TLR2, TLR3, LY9, CD1D1, C1QC, ISG20, CFP, NLRC5, CASP4, SMPDL3B, OASL2,MX1, MX2, CD300C2, H2-K1, PIK3CG, ADGRE1, GBP5, BST2, HERC6, CLEC4N, CD84, C1QA, C1QB, OAS1B,EIF2AK2, IFIH1, OAS3, RSAD2, SP110, OAS2, CD74, PTK2B, IIGP1, NRROS, INPP5D, DHX58, CSF1R, ZBP1, IRGM1, LGALS3, HCK, TLR13, IDO2, MYO1G, TRIM25, H2-Q7, FCGR1, CD180, PSMB8, PSMB9, DDX58, PIRB, IFIT3, IFIT2, CD55, IFIT1, IRF5, CD300A, IRF7, H2-T23, THEMIS2, ADAR
response to virus	30	0.0338	4.77E-22	IFIH1, ZC3HAV1, CLU, OAS3, RSAD2, TLR3, OAS2, CCL5, CXCL10, ISG20, OASL2, DDX60,MX1, MX2, DHX58, HYAL1, BST2, TLR13, IFIT3, DDX58, IFIT2, IFIT1, IFI27L2A, OAS1B, HSPB1, TGTP1, TGTP2, OAS1A, EIF2AK2, ADAR
innate immune response	56	0.0632	3.25E-19	APOBEC3, ZC3HAV1, TLR2, TLR3, LY9, CD1D1, C1QC, ISG20, CFP, NLRC5, CASP4,<br>SMPDL3B, OASL2, MX1, MX2, BST2, HERC6, CLEC4N, CD84, C1QA, C1QB, OAS1B,<br>OAS1A, TREM2, EIF2AK2, IFIH1, TRIM14, OAS3, RSAD2, SP110, OAS2, PTK2B, FCER1G, IIGP1, NRROS, DHX58, CSF1R, ZBP1, TYROBP, IRGM1, LGALS3, HCK, TLR13, TRIM25, FCGR1, CD180, DDX58, IFIT3, IFIT2, CD55, IFIT1, CYBB, IRF5, IRF7, CLEC7A, ADAR
cellular response to interferon-beta	21	0.0237	3.38E-19	IRGM1, GBP6, IRGM2, IFI47, TLR3, STAT1, IFIT3, IFIT1, IGTP, F830016B08RIK, GM4841, IIGP1, TGTP1, IFI209, TGTP2, IFI203, GBP3, GM4951, GBP2, IFI205, IFI204
defense response to virus	35	0.0395	1.49E-17	APOBEC3, A230050P20RIK, IFIH1, APOBEC1, ZC3HAV1, OAS3, SLFN8, RSAD2, TLR3, OAS2, ISG20, CXCL10, NLRC5, OASL2, DDX60, MX1, MX2, DHX58, ZBP1, PTPRC, IL6, BST2, TRIM34A, TRIM25, IFIT3B, STAT2, IFIT3, DDX58, IFIT2, IFIT1, IRF5, OAS1B, OAS1A, EIF2AK2, ADAR
inflammatory response	39	0.044	1.24E-10	C3AR1, CCL3, CCL2, LXN, TLR2, CCL8, TLR3, CCL5, CCL4, CCL7, MMP25, CXCL10, SLC11A1, CASP4, SMPDL3B, NRROS, FAS, CSF1R, PIK3CG, HYAL1, IL6, GBP5, C5AR1, NCF1, HCK, TLR13, EPHX2, CD180, CYBB, LTB4R1, CCR5, STAB1, LTB4R2, PLA2G7, ALOX5, CLEC7A, NGFR, THEMIS2, PTAFR
neutrophil chemotaxis	17	0.0192	8.03E-10	CCL3, CCL2, C5AR1, LGALS3, PREX1, CCL8, NCKAP1L, ITGB2, CCL5, CCL4, VAV1, CCL7, ITGAM, TGFB2, FCGR3, PPBP, FCER1G
immune response	32	0.0361	3.07E-09	CCL3, CCL2, ENPP2, TNFSF15, OAS3, TLR2, CCL8, OAS2, CCL5, CCL4, CD24A, CCL7, CD74, CXCL10, OASL2, NRROS, FAS, FYB, H2-K1, IL6, CTSS, VAV1, TNFSF11, CCR5, PPBP, FCGR2B, CD274, OAS1B, TGTP1, TGTP2, NGFR, LCP2
negative regulation of viral genome replication	12	0.0135	3.85E-09	A230050P20RIK, APOBEC3, BST2, ZC3HAV1, OAS1B, OAS3, RSAD2, CCL5, EIF2AK2, MX2,ISG20, ADAR
cellular response to interferon-gamma	16	0.018	5.70E-09	MRC1, GBP7, GBP6, CCL3, CCL2, GBP5, GBP9, EDN1, TLR3, CCL8, CCL5, CCL4, H2-Q7, CCL7, GBP3, GBP2
positive regulation of cell migration	25	0.0282	9.31E-08	C3AR1, CCL3, PDGFB, FERMT3, PREX1, EDN1, CCL5, AQP1, MMP3, CXCL10, TGFB2, PTK2B, FOXF1, GPNMB, CSF1R, NTF3, MYO1F, MCAM, EPHA1, KDR, CORO1A, SEMA4F, CD274, LAMC2, SEMA4D
defense response to protozoan	10	0.0113	3.94E-07	SLC11A1, GBP7, IL6, CD37, GBP6, IRGM2, GBP9, IIGP1, GBP3, GBP2
defense response to Gram-positive bacterium	16	0.018	4.62E-07	GBP7, LYZ2, GBP6, HIST1H2BC, C5AR1, NCF1, HCK, HIST1H2BG, GBP9, TLR2, MYO1F, ACP5, CHGA, H2-T23, GBP3, GBP2
cell adhesion	39	0.044	1.29E-06	CADM3, CADM4, ATP1B2, CLSTN3, FERMT3, ITGB4, DSCAML1, BCAM, ITGB2, LY9, CD24A, ITGAM, DCHS1, CGREF1, LGALS3BP, LPXN, CD93, PTK2B, TGFBI, ESAM, GPNMB, DPP4, DPT, ICAM1, F11R, EGFL6, EFS, CELSR2, MCAM, EPHA1, CD84, SIGLEC1, LAMA1, LAMA5, STAB1, CDON, ITGA7, CNTN2, LAMC2

Group2 : Blue, 385 genes

Term	Count	%	PValue	Genes
negative regulation of cell proliferation	49	0.0283	1.19E-07	PTGS2, CPEB1, FNTB, CDKN2A, CDKN2B, PTGES, KIFAP3, GATA3, FOSL1, PTPRK, GTPBP4, CLMN, WNK2, ESR2, PIM2, PTPRU, SSTR4, DDR1, MSX1, EREG, BTG2, PTPRV, TGIF1, ROR2, WNT9A, HIST1H2AB, HIST1H2AC, HIST1H2AD, SOX7, TIMP2, ITGB1, LIF, MSX2, BCL11B, AXIN2, CHD5, TES, IL1RL1, BECN1, SMAD6, TAX1BP3, SH3BP4, CDKN1A, PKP2, RPS6KA2, GDF11, BMP7, TOB1, F2R
nervous system development	45	0.026	2.59E-06	ENAH, RITA1, SPG7, PLXNA4, GRIP1, IMPACT, BRSK1, TTLL7, ZIC1, ITM2C, SLC7A5, GDNF, ZIC3, ZIC2, FOS, BDNF, APOB, CRMP1, SEMA7A, GATA3, NGRN, SEMA3E, GPSM1, LMTK2, AVIL, POU3F3, ROBO3, CHD5, OLFM1, INA, RBFOX1, GLRB, TRNP1, CHAC1, FMR1, HES6, MAFK, SLIT1, CDK5, FZD6, NBL1, NME1, ITGA8, ZIC5, CHN1

Group3: Red, 1214 genes

Term	Count	%	PValue	Genes
cell cycle	122	0.0537	1.60E-24	PRC1, DBF4, AURKA, AURKB, FOXO4, CDCA8, OIP5, CDKN2C, INCENP, TLK1, CCNA2, CDCA5, CDCA3, LIG1, RBL1, PPP1CC, NCAPD3, ESCO2, FOXN3, NCAPD2, UHRF1, MAD2L1, TIMELESS, SPAG5, MCMBP, CAMK1, PMP22, NEK6, DSCC1, TICRR, NEK2, STK10, ANAPC15, ANLN, CNTRL, MYBL2, SPC24, NCAPG2, FBXO5, SKA3, CKAP2, MKI67, IKZF1, NUF2, BRCA2, GAS2, NDC80, GAS1, TET2, REEP3, BRCA1, SUV39H2, CDKN1C, RGS2, PLK1, CHAF1A, CHAF1B, STEAP3, KIF23, CLSPN, E2F7, E2F8, CDT1, KIF2C, CDC45, FANCI, MASTL, CDK14, CABLES1, CDC7, CDC6, CDK1, KIF11, CCNF, TPX2, NUSAP1, CDK6, MCM2, UBE2C, MCM3, MCM4, ECT2, MCM5, CDK2, MCM6, FANCD2, NSL1, BUB1B, MELK, FOXM1, HCFC1, CEP55, NCAPH, MDC1, BUB1, USP37, TXNIP, KAT2B, NASP, DLGAP5, GMNN, KIF18B, CENPE, BIRC5, STRADB, KNSTRN, RACGAP1, CDC25C, SMC2, SMC4, CDC25B, CCNB1, RASSF4, RPS6KA3, CCNB2, KNL1, RASSF2, KIF20B, CKS2, CENPW, MIS18BP1, SMPD3
DNA replication	39	0.0172	7.79E-15	BLM, TICRR, DBF4, POLA1, MCM10, CDT1, RPA2, CDC45, POLE2, POLQ, GINS1, GINS2, CDC6, DTL, PIF1, NASP, LIG1, FAM111A, POLE, MCM2, MCM3, MCM4, BRCA1, MCM5, MCM6, POLD3, RFC3, HELB, POLD1, RRM1, PCNA, MCMBP, NFIC, CHAF1A, CHAF1B, NFIA, DSCC1, REV3L, NFIB
inflammatory response	69	0.0303	3.21E-14	RARRES2, AIF1, IL18, LY86, TLR1, PPARG, TLR4, IL15, CXCR3, TLR6, CXCL12, TLR7, TGFB1, TLR8, TLR9, S1PR3, NLRC4, PTGIR, AGTR2, IL1RAP, TICAM2, REG3G, C5AR2, LYN, C4B, PIK3CD, TNFRSF14, NLRP3, CD163, AGTR1B, AGTR1A, CCR3, TNFAIP3, CAMK1D, HMGB2, CYSLTR1, CXCL5, C3, CCR1, CCL9, NLRP1A, AFAP1L2, PF4, NLRP1B, MAPKAPK2, IL34, CCL6, NAIP6, TNFRSF1B, NAIP2, NAIP5, CNR2, PYCARD, HAVCR2, PLP1, IL1RL2, TRIL, CELA1, PTGFR, AIM2, CCL11, CCL12, GGT5, CYBA, P2RX7, TBXA2R, HDAC9, IGFBP4, CD14
mitotic nuclear division	60	0.0264	5.61E-14	KIF23, AURKA, AURKB, KIF2C, CDCA8, OIP5, INCENP, MASTL, CCNA2, CDCA5, CDCA3, CDC6, CDK1, KIF11, CCNF, TPX2, NUSAP1, ESPL1, UBE2C, CDK2, NCAPD3, NCAPD2, MAD2L1, TIMELESS, SPAG5, NSL1, MCMBP, BUB1B, DYNLT1A, NEK6, NEK2, ANAPC15, ANLN, CEP55, SPC24, NCAPH, NCAPG2, BUB1, USP37, SKA3, FBXO5, NUF2, KIF18B, CENPE, NDC80, BIRC5, CDC25C, KNSTRN, SMC2, REEP3, SMC4, CDC25B, CCNB1, SRSF2, CCNB2, PLK1, KNL1, KIF20B, CENPW, MIS18BP1
cell division	72	0.0317	7.22E-14	KIF23, PRC1, AURKA, AURKB, KIF2C, CDC45, CDCA8, OIP5, INCENP, MASTL, CCNA2, CDCA5, CABLES1, CDK14, CDCA3, CDC7, KIF14, CDC6, CDK1, KIF11, LIG1, CCNF, TPX2, NUSAP1, CDK6, UBE2C, PPP1CC, ECT2, MCM5, CDK2, NCAPD3, NCAPD2, MAD2L1, TIMELESS, SPAG5, NSL1, MCMBP, BUB1B, DYNLT1A, NEK6, NEK2, ANAPC15, ANLN, CNTRL, CEP55, SPC24, NCAPH, NCAPG2, BUB1, USP37, SKA3, FBXO5, NUF2, KIF18B, CENPE, BIRC5, NDC80, RACGAP1, CDC25C, KNSTRN, SMC2, REEP3, SMC4, CDC25B, CCNB1, CCNB2, PLK1, KNL1, KIF20B, CKS2, CENPW, MIS18BP1
immune system process	70	0.0308	2.16E-12	CADM1, LY86, TLR1, TLR4, HP, TLR6, TLR7, TLR8, TLR9, BTK, C1RA, NLRC4, MAP3K5, TMEM173, NOD1, SH2D1B1, IL1RAP, TICAM2, CFH, ERAP1, CLEC4A2, LBP, CSK, CFD, SPON2, PAG1, SYK, ICOSL, LYN, PIK3CD, SERPING1, NLRP3, RUBCN, PRKCB, DCLRE1C, CD86, SERPINA3G, LRMP, CD300LF, CLEC5A, TNFAIP8L2, MFSD6, GPR183, HMGB2, LST1, IFITM1, IFITM2, C3, FCNA, IL4RA, TFEB, UNC93B1, NLRP1B, IL34, SEC14L1, NAIP6, NAIP2, NAIP5, PYCARD, CD4, HAVCR2, CARD9, IL1RL2, TNFRSF13B, SAMHD1, TRIL, AIM2, MARCH1, C1S1, CD14
protein phosphorylation	85	0.0374	7.93E-10	RNASEL, ACVRL1, TTK, AURKA, FER, AURKB, FES, TGFB1, PRKX, DMPK, BTK, CAMKK2, ST3GAL1, ACVR1B, MAP3K5, SLK, AAK1, TLK1, MASTL, PRKACB, CSK, CDK14, MAP2K6, SYK, CDC7, CDK1, TNIK, LYN, SGK3, LIMK1, PRKCH, PKN1, CDK6, PBK, STK4, CDK2, PRKCB, ACVR2A, HIPK2, ROR1, BMP2K, PDGFRA, BUB1B, CAMK1, NRK, NEK6, MELK, CAMK1D, MAP3K11, NEK2, STK10, MKNK2, STK17B, MKNK1, MAPKAPK2, MAP3K1, TEK, BUB1, DYRK2, PIK3R1, AATK, PDK1, TGFBR1, CBL, MET, TGFBR2, BMX, BIRC5, STRADB, FNIP2, CDC25B, CCNB1, NTRK3, P2RX7, RPS6KA3, RPS6KA1, PLK1, ULK2, MAPK14, NTRK2, GRK2, MERTK, IGFBP3, ABL2, MYLK
angiogenesis	46	0.0202	3.62E-09	FGF18, NRP1, ACVRL1, IL18, FGF10, PDE3B, ANPEP, CXCR3, TNFSF12, ENPEP, PRKX, NRCAM, TAL1, S1PR1, HEY1, RSPO3, FAP, TEK, PIK3R6, CALCRL, TMEM100, COL8A1, PLXND1, RAMP1, SCG2, SYK, BMP4, RAMP2, FZD8, VAV3, EPAS1, TGFBR1, VAV2, NDNF, VEGFB, CCL12, VEGFC, NOTCH1, VEGFD, MEOX2, SRPX2, MAPK14, ECSCR, RBPJ, TNFAIP2, PLAU
signal transduction	147	0.0647	8.54E-09	FGF18, GNA15, MPZL1, STAT5A, STAT5B, GNA12, TLR1, CDKN2AIPNL, PLPPR4, IQGAP2, PDE3B, TLR4, IL15, TLR6, TLR7, TLR8, IQGAP1, RGL1, TLR9, CD48, S1PR2, S1PR3, AGTR2, PTGIR, S1PR1, GAB1, STARD8, DEPDC1A, GNG2, HCAR1, C5AR2, ICOSL, RREB1, PLXNB3, MRGPRF, STK4, HHEX, GAPVD1, AGTR1B, LPAR5, ARRB2, ARRB1, AGTR1A, CD34, CCR3, CX3CR1, CAMK1, OPHN1, TGFBRAP1, IL1R1, PLXNC1, CYSLTR1, NPY6R, P2RY6, KRAS, P2RY2, CNR2, RASAL3, PLXND1, GPR157, TGFBR1, IL1RL2, BMX, P2RX4, PDE7B, GNGT2, GPR34, CDC42SE2, RGS3, ULK2, PLCG2, TBXA2R, HIVEP3, RAP1A, RAP1B, GRK2, GPR162, ACVRL1, GPR160, PPARG, GJA1, ARHGAP18, RASSF8, ARHGAP17, NFKB1, CXCR3, FER, ARHGAP15, ARHGAP12, ARHGAP21, EDNRA, ARHGAP4, ACVR1B, EDNRB, ARHGAP6, ARHGAP20, CXCR4, IL1RAP, GUCY1A2, TICAM2, CALCRL, ARHGAP11A, PLCB2, ARHGAP9, RAP2A, PTGER3, PTGER4, LYN, CMKLR1, ARHGAP28, PKN1, ARHGAP29, ARRDC3, ARHGAP25, CD83, ADRB1, INPP4A, SRGAP2, RIN3, GPR183, PPP1R12B, CCR1, SLC39A12, GPR65, GPRC5B, ARHGAP39, PLCL2, PLCL1, PDE1A, SH2B3, ZFP217, RASA4, PIK3R1, FZD8, NPY1R, PTGFR, RACGAP1, FZD4, P2RY12, RASSF4, P2RY13, RASSF2, CHN2, EPOR, ADGRL3, SMPD3, ARAP1
intracellular signal transduction	63	0.0277	1.38E-08	ADCY3, ADCY7, PRKAG2, GNA12, ITSN1, BTK, AGTR2, NOD1, GAB2, GUCY1A2, RAPGEF5, DEPDC1A, TLK1, MASTL, CSK, PLCB2, PAG1, SYK, ARHGEF3, TNIK, SGK3, SOCS2, LYN, ARHGEF6, PRKCH, PKN1, ARHGAP29, STK4, ECT2, PRKCB, TNS3, TNS1, SDCBP, MKNK2, STK17B, MKNK1, AKAP13, ASB13, PLCL2, PLCL1, DGKD, ASB10, TEK, SH2B3, RASA4, SCG2, BLNK, PDK1, GPR155, VAV3, TGFBR1, BMX, RACGAP1, VAV2, RPS6KA3, RPS6KA1, ADCY9, MAPK14, PLCG2, DGKZ, DEPTOR, TGFBR3, CHN2
phosphorylation	83	0.0365	6.25E-08	SEPHS2, ACVRL1, TTK, AURKA, FER, AURKB, FES, PRKX, DMPK, BTK, CAMKK2, ACVR1B, MAP3K5, SLK, TLK1, MASTL, PRKACB, CSK, IPMK, CDK14, MAP2K6, SYK, CDC7, CDK1, PDXK, TNIK, LYN, SGK3, LIMK1, PIK3CD, PRKCH, PKN1, CDK6, PBK, STK4, CDK2, PRKCB, ACVR2A, HIPK2, BMP2K, PDGFRA, BUB1B, CAMK1, NRK, NEK6, PRPS2, MELK, CAMK1D, MAP3K11, PFKFB4, NEK2, STK10, MKNK2, STK17B, MKNK1, MAPKAPK2, TK1, MAP3K1, TEK, BUB1, DYRK2, CERK, AATK, PDK1, NADK2, TGFBR1, MET, TGFBR2, BMX, NTRK3, KHK, RPS6KA3, RPS6KA1, PLK1, ULK2, MAPK14, NTRK2, DGKZ, GRK2, PIP4K2A, MERTK, ABL2, MYLK
innate immune response	60	0.0264	1.82E-07	ELF4, LY86, TLR1, TLR4, NFKB1, FER, TLR6, FES, TLR7, TLR8, TLR9, BTK, C1RA, NLRC4, MAP3K5, TMEM173, NOD1, SH2D1B1, IL1RAP, CFH, TICAM2, CLEC4A2, LBP, CSK, CFD, SPON2, SYK, LYN, C4B, PIK3CD, SERPING1, NLRP3, CLEC5A, TNFAIP8L2, HMGB2, IFITM1, IFITM2, C3, FCNA, UNC93B1, NLRP1A, NLRP1B, IL34, SEC14L1, NAIP6, NAIP2, NAIP5, PYCARD, HAVCR2, CARD9, IL1RL2, BMX, SAMHD1, TRIL, AIM2, MID2, CYBA, C1S1, ABL2, CD14
mitotic sister chromatid segregation	12	0.0053	1.84E-07	CDCA8, MAD2L1, PLK1, NEK2, CENPA, SPAG5, NSL1, KIF18B, NUSAP1, NDC80, ESPL1, KNSTRN
response to lipopolysaccharide	37	0.0163	2.81E-07	HMGB2, CXCL5, STAT5B, FGF10, TLR4, PF4, MAPKAPK2, COMT, FER, IL12RB2, EDNRA, VCAM1, EDNRB, PTGIR, TNFRSF1B, TNFRSF11A, NLRC3, CASP9, FMO1, IL10RA, CNR2, TRPV4, LBP, SPON2, PTGER3, PTGER4, MAOB, TNFRSF14, PTGFR, NOTCH1, RPS6KA3, P2RX7, PENK, KCNJ8, MAPK14, PLCG2, TBXA2R
endocytosis	35	0.0154	3.05E-07	SNX6, SNX5, AP2S1, SNX8, SNX2, ITSN2, ITSN1, DAB2, RINL, AAK1, NECAP2, WIPF1, FCHO2, HIP1, GHR, CLN3, DENND1A, DPYSL2, RUBCN, MARCH3, EPS15, AP2A2, NPC1, FNBP1, GAPVD1, ARRB2, TFRC, ARRB1, LRP12, LRP6, FKBP15, OPHN1, SORT1, SNX32, SCARA5
chemotaxis	27	0.0119	3.08E-07	HMGB2, RARRES2, IL16, CYSLTR1, CXCL5, CCR1, CCL9, FGF10, PF4, CXCR3, FER, FES, CXCL12, CCL6, S1PR1, CXCR4, ROBO2, C5AR2, LYN, CMKLR1, PIK3CD, CCL11, CCL12, CCR3, ECSCR, CX3CR1, PDGFRA
positive regulation of protein phosphorylation	35	0.0154	4.57E-07	RARRES2, C3, AIF1, IQGAP3, IL34, GPRC5B, TGFB1, EDNRA, EDNRB, RGMA, MAP3K5, DAB2, KRAS, CXCR4, TEK, PELI2, PIK3R1, MAP2K6, BMP4, WDFY2, CLN3, FZD8, RAP2A, PTGER3, TNIK, PTGER4, LRRN3, PRR5L, ADIPOQ, STK4, ACVR2A, P2RX7, ARRB2, ARRB1, HIPK2
toll-like receptor signaling pathway	10	0.0044	8.30E-07	RPS6KA3, CD86, TLR1, UNC93B1, TLR4, MAPKAPK2, TLR6, TLR7, TLR8, TLR9
chromosome segregation	22	0.0097	1.27E-06	KIF11, NEK2, SLC25A5, CENPF, CENPE, ESPL1, NDC80, BIRC5, KNSTRN, BRCA1, ESCO2, KIF2C, OIP5, KNL1, SPAG5, INCENP, NSL1, BUB1, CENPW, SKA3, TOP2A, NEK6

Group4: Black, 707 genes

Term	Count	%	PValue	Genes
cholesterol biosynthetic process	7	0.0127	1.35E-05	TM7SF2, MVD, HMGCR, HMGCS1, LSS, IDI1, DHCR24
cell adhesion	23	0.0416	2.61E-05	FLRT3, ATP1B1, CADM2, ICAM5, NID2, TINAGL1, COL16A1, THY1, VCL, PCDH18, LAMA2, SORBS3, OMD, FAT4, SORBS2, COMP, SULF1, COL6A2, COL6A1, HAS2, NEGR1, COL8A2, CDH11

Supplementary Table 3

Day 0: Csnk2b KO vs. WT. Summary of the fold changes

GO category	Min	1st Quartile	Median	3rd Quartile	Max
angiogenesis	0.416	0.459	0.461	0.578	0.631
response to hypoxia	0.273	0.445	0.551	0.595	0.665
vasculogenesis	0.416	0.518	0.559	0.647	0.665
blood vessel development	0.273	0.503	0.531	0.648	0.665
retinoic acid metabolic process	0.349	0.459	0.461	0.578	0.631
response to ethanol	0.349	0.479	0.562	0.524	0.586
cell adhesion	0.318	0.507	0.563	0.604	0.657
negative regulation of viral entry into host cell	0.586	0.595	0.656	0.659	0.667
kidney development	0.273	0.418	0.552	0.616	0.665
nervous system development	0.386	0.507	0.596	0.628	0.646

Day 7: Csnk2b KO vs. WT. Summary of the fold changes

GO category	Min	1st quartile	Median	3rd quartile	Max
immune system process	0.032	0.199	0.252	0.382	0.496
cell cycle	0.127	0.274	0.324	0.4	0.499
inflammatory response	0.07	0.172	0.247	0.236	0.364
mitotic nuclear division	0.127	0.268	0.318	0.384	0.489
cell division	0.127	0.269	0.337	0.391	0.489
innate immune response	0.039	0.214	0.266	0.368	0.496
chemotaxis	0.074	0.174	0.255	0.354	0.479
DNA replication	0.16	0.28	0.336	0.399	0.484
cell adhesion	0.032	0.176	0.296	0.374	0.488
mitotic sister chromatid segregation	0.242	0.277	0.335	0.367	0.462
signal transduction	0.058	0.202	0.296	0.396	0.499
toll-like receptor signaling pathway	0.05	0.149	0.241	0.432	0.496
positive regulation of protein phosphorylation	0.031	0.211	0.324	0.368	0.486
chromosome segregation	0.188	0.252	0.29	0.361	0.417
adaptive immune response	0.032	0.214	0.287	0.361	0.479
angiogenesis	0.101	0.274	0.337	0.414	0.475
positive regulation of cell migration	0.074	0.231	0.323	0.367	0.489

Supplementary Fig. 14a: RT-PCR analysis (fold)

None	HO
0.796258	2.909357
0.998937	3.165003
0.977874	3.403715
1.226911	3.702725

Supplementary Fig. 14b: RT-PCR analysis (fold)

Csnk2b		Runx2	
Csnk2b$^{fl/fl}$	Csnk2b^{Osx}	Csnk2b$^{fl/fl}$	Csnk2b^{Osx}
1.1496865	0.4666751	0.9206341	1.7141449
1.1300844	0.4000301	0.9049373	1.4693512
0.8675105	0.1746171	1.0965599	1.4902766
0.8527194	0.1496804	1.0778636	1.2774531

Supplementary Fig. 14d: Number of RUNX2+ cells/bone area (mm^2)

Csnk2b$^{fl/fl}$	Csnk2b^{Osx}
819.39653	396.53586
978.2362	719.91279
864.28226	732.65441
1122.639	218.94294
1128.9655	376.71706
754.09233	173.93321

Supplementary Fig. 14e: RT-PCR analysis (fold)

Csnk2b		Runx2	
Csnk2b$^{fl/fl}$	Csnk2b^{Osx}	Csnk2b$^{fl/fl}$	Csnk2b^{Osx}
1.2272333	0.0004422	1.3007272	0.74264
0.8596341	0.0005855	0.9111139	0.9833495
1.1250642	0.0260897	1.0515693	0.8439409
0.7880682	0.0345461	0.7365876	1.1174847

Supplementary Fig. 14g: Number of RUNX2+ cells/bone area (mm^2)

Csnk2b$^{fl/fl}$	Csnk2b^{Osx}
1491.3545	298.94026
763.88583	667.87642
807.48898	630.16452
908.98095	705.08934
918.15101	630.2521
830.89729	711.59183

Supplementary Fig. 15a: Alkaline phosphatase activity (fold)

0 uM	0.01 uM	0.1 uM	1 uM
0.99631	0.793358	0.811808	0.387454
1.125461	1.051661	0.664207	0.516605
0.940959	0.793358	0.756458	0.479705
0.97786	0.867159	0.627306	0.516605
0.940959	0.867159	0.535055	0.498155
1.01476	0.922509	0.645756	0.479705

Supplementary Fig. 15b: Alizarin Red staining (fold)

0 uM	0.01 uM	0.1 uM	1 uM
1.286058	0.427205	0.215085	0.06972
0.795725	0.460776	0.225096	0.069567
1.010695	0.731676	0.16734	0.070984
1.137915	0.542015	0.214829	0.069554
0.851615	0.493975	0.225313	0.067638
0.917988	0.521175	0.19565	0.066566

Supplementary Fig. 15c: OG2-luciferase activity (fold)

-	Runx2 WT+ i-CK2		
-	0	0.1 uM	1 uM
0.84	3.28	1.44	0.88
1.4	2.68	2.56	1.16
0.76	3	1.72	0.68

Supplementary Fig. 15d: Alkaline phosphatase activity (fold)

0 uM	0.3 uM	1 uM	3 uM	10 uM
1.028674	0.84767	0.844086	0.81362	0.650538
1.001792	0.858423	1.062724	0.784946	0.682796
1.030466	0.946237	1.030466	0.752688	0.729391
0.937276	0.973118	1.05914	0.61828	0.598566

Supplementary Fig. 15e: Alizarin Red staining (fold)

0 uM	0.3 uM	1 uM	3 uM	10 uM
1.01281	0.952034	0.921506	0.665485	0.660641
0.981268	0.904028	0.856766	0.681375	0.587631
1.019213	1.046455	0.965213	0.692385	0.573233
0.978151	1.039137	0.859138	0.64282	0.68937
1.009628	0.97497	0.826987	0.691877	0.666603
0.998919	1.050619	0.839319	0.668738	0.601352

Supplementary Fig. 15f: RT-PCR analysis (fold)

Vehicle	i-HAUSP
0.993788	1.521453
1.00768	1.544378
0.992339	1.4385
1.006203	1.460167

Supplementary Fig. 16a: microCT analysis

Tb.BV/TV (1)		Tb.N (mm^{-1})		Tb.Th (mm)		C.Th (mm)	
Csnk2b$^{fl/fl}$	Csnk2b^{Ctsk}	Csnk2b$^{fl/fl}$	Csnk2b^{Ctsk}	Csnk2b$^{fl/fl}$	Csnk2b^{Ctsk}	Csnk2b$^{fl/fl}$	Csnk2b^{Ctsk}
0.1943	0.1824	5.1735	5.3371	0.0507	0.0464	0.157	0.1824
0.1206	0.189	5.7694	5.2616	0.0387	0.049	0.1565	0.189
0.2169	0.1505	5.2536	4.8905	0.0463	0.0484	0.1792	0.1505
0.1836	0.2268	5.0269	5.6297	0.049	0.0481	0.1897	0.2268
0.1295	0.1608	5.5258	4.8273	0.0364	0.0514	0.1452	0.1608

Supplementary Fig. 16b: RT-PCR analysis (fold)

	Pre-osteoclast				Mature osteoclast			
Csnk2a1	1.060232	0.939771	1.060232	0.939771	0.897751	0.91724	0.901484	0.921065
Csnk2a2	1.058766	0.938471	1.061705	0.941076	0.892171	0.911536	0.919156	0.939132
Csnk2b	1.041491	0.923159	1.078967	0.956377	0.914243	0.934101	0.828543	0.84654

Supplementary Fig. 18a: Cell migration (fold)

BM	WT_0d	KO_0d	WT_7d	KO_7d	FGF-2
0.982521	0.945138	1.067258	1.062614	1.173916	1.264524
0.976407	0.960186	1.056032	1.524555	1.048205	2.02274
1.041072	0.943805	1.062972	1.147659	1.279605	1.355215
	0.960186	1.121931	1.572707	0.934755	
	0.943805	1.026674	1.031874	1.065032	
	1.093008	1.163919	1.524555	1.09791	

Supplementary Fig. 18c: Tube formation (fold)

BM	WT_0d	KO_0d	WT_7d	KO_7d	FGF-2
1.049202	1.660184	1.805256	1.732449	1.852096	1.83385
0.950846	1.715619	1.818612	1.616153	2.506407	1.597916
1.28018	1.85183	2.621546	2.124403	1.850037	2.053579
1.198562	1.611986	1.936608	2.301334	1.572437	2.301991
0.84704	1.719302	1.850441	1.625866	1.916201	1.929029
0.735509					2.070068
0.938661					2.105313

Supplementary Fig. 19: RT-PCR analysis (fold)

Csnk2b		Hif1a		Vegfa		Epo		Slit3		Cxcl9	
WT	KO	WT	KO	WT	KO	WT	KO	WT	KO	WT	KO
0.970589	0.414701	0.983953	1.162378	0.951272	1.117445	0.995212	0.629986	1.029472	0.885157	0.945596	0.190607
1.064009	0.431329	0.995193	1.125089	0.962139	1.081598	1.091003	0.655247	0.876595	0.797364	1.036611	0.19825
0.937587	0.408842	1.149954	1.053733	1.038269	0.859299	0.957145	0.965066	1.029472	0.885157	0.962568	0.171477
1.027831	0.425235	1.16309	1.01993	1.050129	0.831733	0.957145	0.965066	1.064684	0.996768	1.055216	0.178353
