# Supplementary material for: A RUNX2 stabilization pathway mediates physiologic and pathologic bone formation
Source: Nat Commun. 2020 May 8;11:2289. doi: 10.1038/s41467-020-16038-6 (PMC7210266; doi:10.1038/s41467-020-16038-6)
Supplement: Supplementary file 2 — Reporting Summary [file 41467_2020_16038_MOESM2_ESM.pdf]

nature research

Corresponding author(s): Jae-Hyuck Shim

Last updated by author(s): Mar 26, 2020

Reporting Summary

Nature Research wishes to improve the reproducibility of the work that we publish. This form provides structure for consistency and transparency in reporting. For further information on Nature Research policies, see Authors & Referees and the Editorial Policy Checklist.

Statistics

For all statistical analyses, confirm that the following items are present in the figure legend, table legend, main text, or Methods section.

n/a	Confirmed	
☐	☒	The exact sample size (*n*) for each experimental group/condition, given as a discrete number and unit of measurement
☐	☒	A statement on whether measurements were taken from distinct samples or whether the same sample was measured repeatedly
☐	☒	The statistical test(s) used AND whether they are one- or two-sided<br>*Only common tests should be described solely by name; describe more complex techniques in the Methods section.*
☐	☒	A description of all covariates tested
☐	☒	A description of any assumptions or corrections, such as tests of normality and adjustment for multiple comparisons
☐	☒	A full description of the statistical parameters including central tendency (e.g. means) or other basic estimates (e.g. regression coefficient) AND variation (e.g. standard deviation) or associated estimates of uncertainty (e.g. confidence intervals)
☐	☒	For null hypothesis testing, the test statistic (e.g. *F*, *t*, *r*) with confidence intervals, effect sizes, degrees of freedom and *P* value noted<br>*Give P values as exact values whenever suitable.*
☒	☐	For Bayesian analysis, information on the choice of priors and Markov chain Monte Carlo settings
☒	☐	For hierarchical and complex designs, identification of the appropriate level for tests and full reporting of outcomes
☐	☒	Estimates of effect sizes (e.g. Cohen's *d*, Pearson's *r*), indicating how they were calculated

Our web collection on statistics for biologists contains articles on many of the points above.

Software and code

Policy information about availability of computer code

Data collection	Microsoft Office Excel, GraphPad Prism, FlowJo, Powerpoint
Data analysis	The experimental results were analyzed using GraphPad Prism (ver.8.3.0). All flow cytometry data were analyzed on FlowJo Software. IPA (ver.01-10) was used for pathway analysis from mass spectrometry data. For RNA sequencing, reads are aligned to mouse genome GRCm38 by using STAR aligner (ver.2.3.0e) and mapped reads were indexed using SAMTools (ver.1.9). Gene counts were obtained by HTSeq-Count (ver.0.11.2) to sorted bam files. DESeq2 (ver. 1.4.5) was employed for differential gene expression analysis from RNA sequencing data and functional enrichment analysis was performed with DAVID (ver. 6.7).

For manuscripts utilizing custom algorithms or software that are central to the research but not yet described in published literature, software must be made available to editors/reviewers. We strongly encourage code deposition in a community repository (e.g. GitHub). See the Nature Research guidelines for submitting code & software for further information.

Data

Policy information about availability of data

All manuscripts must include a data availability statement. This statement should provide the following information, where applicable:

- Accession codes, unique identifiers, or web links for publicly available datasets
- A list of figures that have associated raw data
- A description of any restrictions on data availability

All data supporting the findings of this study are presented in the Figures and Supplementary Information files. The source data that was used to generate the figures is provided as a Source Data file.

Field-specific reporting

Please select the one below that is the best fit for your research. If you are not sure, read the appropriate sections before making your selection.

☒ Life sciences ☐ Behavioural & social sciences ☐ Ecological, evolutionary & environmental sciences

For a reference copy of the document with all sections, see nature.com/documents/nr-reporting-summary-flat.pdf

Life sciences study design

All studies must disclose on these points even when the disclosure is negative.

Sample size	Generally, sample sizes were calculated on the assumption that a 30% difference in the parameters measured would be considered biologically significant with an estimate of sigma of 10-20% of the expected mean. Alpha and Beta were set to the standard values of .05 and 0.8, respectively.
Data exclusions	No exclusions.
Replication	All experiments were individually at least 2-3 times in order to confirm reproducibility and findings were consistent.
Randomization	In general, the samples/cells were randomized into different groups prior to treatment. For animal study, genetic knockout mice were randomly chosen for skeletal analysis. For CK2/HAUSP inhibitor treatment for mouse HO models, all the animals were randomly grouped for experiments.
Blinding	Yes, microCT analysis, histology and histomorphometry were performed by individuals (Yeon-Suk Yang, Ren Xu, Na Li) who were blinded to the nature of the mice under analysis (both what specific mouse strains or treatment groups were in the experiment and whether any individual mouse belonged to control versus experimental groups). For other experiments, no blinding was employed as the researcher performing the treatment was also responsible for the analysis.

Reporting for specific materials, systems and methods

We require information from authors about some types of materials, experimental systems and methods used in many studies. Here, indicate whether each material, system or method listed is relevant to your study. If you are not sure if a list item applies to your research, read the appropriate section before selecting a response.

Materials & experimental systems

n/a	Involved in the study	
☐	☒	Antibodies
☐	☒	Eukaryotic cell lines
☒	☐	Palaeontology
☐	☒	Animals and other organisms
☐	☒	Human research participants
☒	☐	Clinical data

Methods

n/a	Involved in the study	
☒	☐	ChIP-seq
☐	☒	Flow cytometry
☒	☐	MRI-based neuroimaging

Antibodies

Antibodies used

For immunoblotting, the following primary antibodies were used: HAUSP (1:1000, Santa Cruz, sc-30164), GAPDH (1:1000, Santa Cruz, sc-25778), HSP90 (1:1000, Santa Cruz, sc-7947), Ubiquitin (1:1000, Santa Cruz, sc-8017), CSNK2A (1:1000, Cell signaling, 2656), CSNK2B (1:1000, abcam, ab133576), RUNX2 (1:1000, Calbiochem, PC287 or 1:1000, Cell signaling, 12556), USP24 (1:500, Proteintech, 13126-1-AP).
For immunohistochemistry, the following primary antibodies were used: HAUSP (1:100, ABclonal A13564), RUNX2 (1:100, MBL, D130-3), CSNK2B (1:100, Abcam, ab133576), CSNK2A (1:100, Abcam, ab70774)
For immunofluorescence, the following antibodies were used: CSNK2B (1:100, Abcam, ab76025), RUNX2 (1:50, Santa Cruz, sc-390351), HAUSP (1:100, Abclonal, A13564), COL1A1 (1:100, Abclonal, A1352), Alexa Fluor 488 (1:400, Thermo A21206) and Alexa Fluor 594 (1:400, Thermo, A11032)

For flow cytometry for human cells, the following antibodies were used: CD31 (1:100, BD biosciences, 560984), CD45 (1:100, BD biosciences, 560976), CD235a (1:100, BD biosciences, 561017), CD90 (1:100, BD biosciences, 566219), CD200 (1:100, BD biosciences, 564114), CD105 (1:100, BioLegend, 323217).

For flow cytometry for mouse cells, the following antibodies were used: Biotin-conjugated CD45 (1:200, ebioscience, 13-0451-81), Tie2 (1:200, ebioscience, 13-5987-81) and Ter119 (1:200, ebioscience, 13-5921-81) with BV421-conjuated streptavidin (1:500, BioLegend, 405226), PE-conjugated CD51 (1:200,ebioscience,12-0512-81), BV605-conjugated Thy1.1-2 (1:200, BioLegend, 202537/140317), APC-conjugated CD200 (1:100, BioLegend, 123809), FITC-conjugated LY51 (1:50,Biolegend,

108305) and PE/Cy7-conjugated CD105 (1:200, BioLegend, 120409).

Validation

All antibodies used for immunoblotting were validated by provider and have been validated in human or mouse cells.
HAUSP, https://www.scbt.com/p/hausp-antibody-h-200
GAPDH, https://www.scbt.com/p/gapdh-antibody-fl-335
HSP90, https://www.scbt.com/p/hsp-90alpha-beta-antibody-h-114
Ubiquitin, https://datasheets.scbt.com/sc-8017.pdf
CSNK2A, https://www.cellsignal.com/products/primary-antibodies/ck2a-antibody/2656
CSNK2B, https://www.abcam.com/casein-kinase-2-beta-antibody-epr1994-ab133576.html
RUNX2, https://www.labome.com/product/EMD-Millipore/PC287-100UG.html, https://www.cellsignal.com/products/primary-antibodies/runx2-d1l7f-rabbit-mab/12556
USP24, https://www.ptglab.com/products/USP24-Antibody-13126-1-AP.htm

All antibodies used for immunohistochemistry were verified by supplier have been tested in human or mouse bone tissue.
HAUSP, https://abclonal.com/catalog-antibodies/HAUSPUSP7PolyclonalAntibody/A13564
RUNX2, https://ruo.mbl.co.jp/bio/e/dtl/A/?pcd=D130-3
CSNK2B, https://www.abcam.com/casein-kinase-2-beta-antibody-epr1994-ab133576.html
CSNK2A, https://www.abcam.com/csnk2a1-antibody-8e5-ab70774.html

All antibodies used for immunofluorescence were validated by provider and have been tested in human tissues or mouse cells.
CSNK2B, https://www.abcam.com/casein-kinase-2-beta-antibody-ep1995y-ab76025.html
RUNX2, https://www.scbt.com/p/runx2-antibody-f-2
HAUSP, https://abclonal.com/catalog-antibodies/HAUSPUSP7PolyclonalAntibody/A13564
COL1A1, https://abclonal.com/catalog-antibodies/COL1A1PolyclonalAntibody/A1352
Alexa Fluor 488, https://www.thermofisher.com/antibody/product/Donkey-anti-Rabbit-IgG-H-L-Highly-Cross-Adsorbed-Secondary-Antibody-Polyclonal/A-21206
Alexa Fluor 594, https://www.thermofisher.com/antibody/product/Goat-anti-Mouse-IgG-H-L-Highly-Cross-Adsorbed-Secondary-Antibody-Polyclonal/A-11032

All antibodies used for flow cytometry of human stem cells were validated by supplier and have been verified using isotype control.
CD31, https://www.bdbiosciences.com/us/applications/research/stem-cell-research/cancer-research/human/fitc-mouse-anti-human-cd31-wm59-also-known-as-wm-59/p/560984
CD45, https://www.bdbiosciences.com/eu/applications/research/stem-cell-research/cancer-research/human/fitc-mouse-anti-human-cd45-hi30/p/560976
CD235a, https://www.bdbiosciences.com/us/reagents/research/antibodies-buffers/immunology-reagents/anti-human-antibodies/cell-surface-antigens/fitc-mouse-anti-human-cd235a-ga-r2-hir2/p/561017
CD90, https://www.bdbiosciences.com/ca/applications/research/stem-cell-research/cancer-research/human/buv395-mouse-anti-human-cd90-5e10/p/566219
CD200, https://www.bdbiosciences.com/us/reagents/research/antibodies-buffers/immunology-reagents/anti-human-antibodies/cell-surface-antigens/bv421-mouse-anti-human-cd200-mrc-ox-104/p/564114
CD105, https://www.biolegend.com/fr-fr/products/pe-cy7-anti-human-cd105-antibody-7167

All antibodies used for flow cytometry of mouse stem cells were validated by supplier and have been verified using isotype control.
CD45, https://www.thermofisher.com/antibody/product/CD45-Monoclonal-Antibody-30-F11-Biotin-eBioscience/13-0451-81
Tie2, https://www.thermofisher.com/antibody/product/CD202b-TIE2-Antibody-clone-TEK4-Monoclonal/13-5987-81
Ter119, https://www.thermofisher.com/antibody/product/TER-119-Antibody-clone-TER-119-Monoclonal/13-5921-81
BV421-SA, https://www.biolegend.com/en-us/products/brilliant-violet-421-streptavidin-7297
CD51, https://www.thermofisher.com/antibody/product/CD51-Integrin-alpha-V-Antibody-clone-RMV-7-Monoclonal/12-0512-81
Thy1.1, https://www.biolegend.com/fr-fr/products/brilliant-violet-605-anti-rat-cd90-mouse-cd90-1-thy-1-1-antibody-8952
Thy1.2, https://www.biolegend.com/fr-fr/products/brilliant-violet-605-anti-mouse-cd90-2-thy-1-2-antibody-7866
CD200, https://www.biolegend.com/fr-fr/products/apc-anti-mouse-cd200-ox2-antibody-7338
LY51, https://www.biolegend.com/fr-fr/products/fitc-anti-mouse-ly-51-antibody-177
CD105, https://www.biolegend.com/fr-fr/products/pe-cy7-anti-mouse-cd105-antibody-4573

Eukaryotic cell lines

Policy information about cell lines

Cell line source(s)

Cell lines (C3H10T1/2 and HEK293) were purchased from ATCC. Human bone marrow-derived stromal cells (hBMSCs) were purchased from Cyagen Biosciences.

Authentication

Cell authentication by the vendor.

Mycoplasma contamination

All of our cell lines were tested for mycoplasma contamination in a regular basis (4 times/year) and they were all confirmed to be negative.

Commonly misidentified lines
(See ICLAC register)

No misidentified cell lines used in the study.

Animals and other organisms

Policy information about studies involving animals; ARRIVE guidelines recommended for reporting animal research

Laboratory animals	Csnk2b floxed mice (Csnk2bfl/fl) mice were generated as previously described (Nat Immunol 16, 267-275, 2015). Hausp floxed mice (Hauspfl/fl) mice were generated as previously described (Cell Death Differ 18, 1366-1375, 2011; Nat Med 22, 1180-1186, 2016) and maintained on a mixed background of 129Sv and C57BL/6J. Runx2 fl/fl mice were previously reported (Development 143, 211-218, 2016). Transgenic mice expressing Cre recombinase under control of the prx1 promoter (Prx1-Cre), osterix promoter (Osx-Cre), cathepsin K promoter (Ctsk-Cre) were mated with Csnk2b floxed mice or Hausp floxed mice to obtain various conditional KO mice. For skeletal stem cell preparation, embryonic day 17.5 (E17.5) embryos were used. Skeletal preparation was applied for E16.5 embryos or postnatal day 10 (P10) pups. For microCT, histology and histomorphometry, 2-month-aged male or female mice were used and analyzed.
Wild animals	No wild animals used.
Field-collected samples	No field samples collected.
Ethics oversight	All animals were used in accordance with the NIH Guide for the Care and Use of Laboratory Animals and were handled according to protocols approved by the Weill Cornell Medical College subcommittee and the University of Massachusetts Medical School on animal care (IACUC).

Note that full information on the approval of the study protocol must also be provided in the manuscript.

Human research participants

Policy information about studies involving human research participants

Population characteristics	We note that only histology data (H&E, immunohistochemistry and immunofluorescence for expression of type 1 collagen, RUNX2, CSNK2 and HAUSP) is currently provided for the analysis of the human heterotopic ossification (HO) tissues.<br>The individuals included seven patients (3 male and 4 females, previously healthy, nonsmoking individuals; age ranging from 29 to 67 years) with pain or decreased hip movement.
Recruitment	Patients with diagnosis of heterotopic ossification underwent exclusion surgery. After surgery, heterotopic bone samples for this study were stored and analyzed in Yonsei University Severance Hospital, Korea.
Ethics oversight	The samples were obtained from human patients in Yonsei University Severance Hospital, Korea under institutional review board approval (IRB No.4-2017-1223).

Note that full information on the approval of the study protocol must also be provided in the manuscript.

Flow Cytometry

Plots

Confirm that:

☒ The axis labels state the marker and fluorochrome used (e.g. CD4-FITC).

☒ The axis scales are clearly visible. Include numbers along axes only for bottom left plot of group (a 'group' is an analysis of identical markers).

☒ All plots are contour plots with outliers or pseudocolor plots.

☒ A numerical value for number of cells or percentage (with statistics) is provided.

Methodology

Sample preparation	For human skeletal stem cell preparation, human bone marrow aspirate (BMA) purchased from StemExpress (BMEDT010F) was incubated for 10 min at room temperature with BD Pharm Lyse hypotonic lysis buffer (BD Biosciences, 555899) for RBC lysis. Cells were washed with cold FACS buffer twice.<br>For mouse skeletal stem cell preparation, E17.5 Csnk2bfl/fl and Csnk2bPrx1 embryonic limbs were dissociated by mechanical and enzymatic digestion (1mg/ml of Collagenase P (Roche, 11213857), 2 mg/ml of Dispase II (Roche, 10165859001), 1 mg/ml of Hyaluronidase (Sigma, H3506) and 10000 unit/ml of DNase I (Roche, 4716728001)) for 1 hour at 37°C under gentle agitation. After digestion, cells were passed through 40 um cell strainer and washed with cold PBS (pH 7.2) containing 0.5% BSA (Fraction V) and 1 mM EDTA.
Instrument	LSRII flow cytometer (BD Biosciences) for analysis and FACS Aria II SORP cell sorter (Becton Dickinson) for cell sorting
Software	FlowJo software (TreeStar)
Cell population abundance	In general, flow cytometry was limited to analysis and not sorting. It was not feasible to run a purity check on the post-sort population of cells.

Gating strategy

Please see detailed description in Supplementary Figure 3 and 6.

☒ Tick this box to confirm that a figure exemplifying the gating strategy is provided in the Supplementary Information.
